# Direct reprogramming of human umbilical vein- and peripheral blood-derived endothelial cells into hepatic progenitor cells

**DOI:** 10.1038/s41467-020-19041-z

**Published:** 2020-10-21

**Authors:** Hiroki Inada, Miyako Udono, Kanae Matsuda-Ito, Kenichi Horisawa, Yasuyuki Ohkawa, Shizuka Miura, Takeshi Goya, Junpei Yamamoto, Masao Nagasaki, Kazuko Ueno, Daisuke Saitou, Mikita Suyama, Yoshihiko Maehara, Wataru Kumamaru, Yoshihiro Ogawa, Sayaka Sekiya, Atsushi Suzuki

**Affiliations:** 1 Division of Organogenesis and Regeneration, Medical Institute of Bioregulation, Kyushu University, Fukuoka, 812-8582 Japan; 2 Department of Medicine and Bioregulatory Science, Graduate School of Medical Sciences, Kyushu University, Fukuoka, 812-8582 Japan; 3 Division of Transcriptomics, Medical Institute of Bioregulation, Kyushu University, Fukuoka, 812-8582 Japan; 4 Center for Genomic Medicine, Kyoto University Graduate School of Medicine, Kyoto, 606-8507 Japan; 5 Human Biosciences Unit for the Top Global Course, Center for the Promotion of Interdisciplinary Education and Research, Kyoto University, Kyoto, 606-8507 Japan; 6 Genome Medical Science Project, National Center for Global Health and Medicine, Tokyo, 162-8655 Japan; 7 Division of Bioinformatics, Medical Institute of Bioregulation, Kyushu University, Fukuoka, 812-8582 Japan; 8 Department of Surgery and Science, Graduate School of Medical Sciences, Kyushu University, Fukuoka, 812-8582 Japan; 9 Department of Oral and Maxillofacial Surgery, Graduate School of Dental Science, Kyushu University, Fukuoka, 812-8582 Japan

**Keywords:** Regenerative medicine, Differentiation, Reprogramming, Stem cells, Regeneration

## Abstract

Recent advances have enabled the direct induction of human tissue-specific stem and progenitor cells from differentiated somatic cells. However, it is not known whether human hepatic progenitor cells (hHepPCs) can be generated from other cell types by direct lineage reprogramming with defined transcription factors. Here, we show that a set of three transcription factors, FOXA3, HNF1A, and HNF6, can induce human umbilical vein endothelial cells to directly acquire the properties of hHepPCs. These induced hHepPCs (hiHepPCs) propagate in long-term monolayer culture and differentiate into functional hepatocytes and cholangiocytes by forming cell aggregates and cystic epithelial spheroids, respectively, under three-dimensional culture conditions. After transplantation, hiHepPC-derived hepatocytes and cholangiocytes reconstitute damaged liver tissues and support hepatic function. The defined transcription factors also induce hiHepPCs from endothelial cells circulating in adult human peripheral blood. These expandable and bipotential hiHepPCs may be useful in the study and treatment of human liver diseases.

## Abstract (web summary)

The conditions to induce human hepatic progenitor cells from other cell types are unclear. Here, the authors reprogram human endothelial cells to hepatic progenitor cells by expressing FOXA3, HNF1A and HNF6, capable of giving rise to hepatocytes and cholangiocytes that reconstitute damaged liver tissues on transplantation.

## Introduction

Hepatocytes are a valuable cell source for investigating and treating liver diseases. However, it is not easy to obtain an adequate quantity of hepatocytes from living patients and organ donors, and it is also difficult to maintain the hepatic function and proliferative potential of hepatocytes in culture after isolation from the liver. Recent studies have demonstrated that hepatocyte-like cells derived from human pluripotent stem cells (hPSCs) can be used as an alternative to hepatocytes^1^. Because hPSCs are expandable in culture, abundant hepatocyte-like cells can be theoretically obtained by inducing the differentiation of propagated hPSCs. However, controlling the differentiation of hPSCs into hepatic lineages requires complicated processes that imitate embryonic liver development, and currently, there are many different protocols for the hepatic differentiation of hPSCs^2^, which may be confusing for researchers. Moreover, hPSC-derived cells can potentially develop into tumors if their differentiation is not properly regulated.

As another strategy for generating an alternative to hepatocytes, direct reprogramming technology can be used to convert mouse and human fibroblasts into cells resembling hepatocytes without passing through a pluripotent state^3–6^. These induced hepatocyte-like cells (iHepCs) have the morphological and functional properties of hepatocytes and reconstitute hepatic tissues after transplantation into injured mouse livers. Thus human iHepCs (hiHepCs) are useful as an alternative to hepatocytes for examining the pharmacological effects of drugs and developing therapeutic strategies for liver diseases, including improvement of hepatocyte transplantation therapy and artificial-organ hepatic support. However, a large number of hiHepCs are required for medical applications, and the growth of hiHepCs is arrested and requires additional oncogene-dependent cell-growth activation^5,6^. Thus, to facilitate the medical use of reprogrammed hepatocytes, human induced hepatic progenitor cells (hiHepPCs) that can potentially propagate in long-term culture and continuously produce hepatocytes as a descendant may be more valuable than growth-arrested hiHepCs. Recent studies have shown that direct reprogramming technology can be used to induce the conversion of differentiated human somatic cells into various types of tissue-specific stem and progenitor cells, including neural stem cells^7^, neural crest cells^8^, hematopoietic progenitor cells^9^, and intestinal progenitor cells^10^. However, it is not known whether human hepatic progenitor cells (hHepPCs) can be generated from other somatic cells by direct lineage reprogramming with defined transcription factors (TFs).

In this study, we identify a specific combination of TFs that can directly induce the conversion of human umbilical vein endothelial cells (HUVECs) into hiHepPCs, which can maintain their own cell population in long-term monolayer culture and give rise to functional hepatocytes after formation of cell aggregates. In addition to hepatocyte differentiation, hiHepPCs are also capable of differentiating into cells that form cystic epithelial spheroids under three-dimensional (3D) culture conditions, which closely resemble the spheroids formed by human cholangiocytes. These hiHepPC-derived hepatocytes and cholangiocytes can reconstitute injured liver tissues and ameliorate hepatic function after transplantation. Moreover, enforced expression of the defined TFs also enables generation of hiHepPCs from human peripheral blood-derived endothelial cells (HPBECs). The generation of expandable and bipotential hiHepPCs will provide an ideal source of a large number of functional hepatocytes and cholangiocytes, which may contribute to the development of basic research and medical applications for patients with liver diseases.

## Results

### A specific set of three TFs induces hiHepPCs from HUVECs

**Fig. 1:**
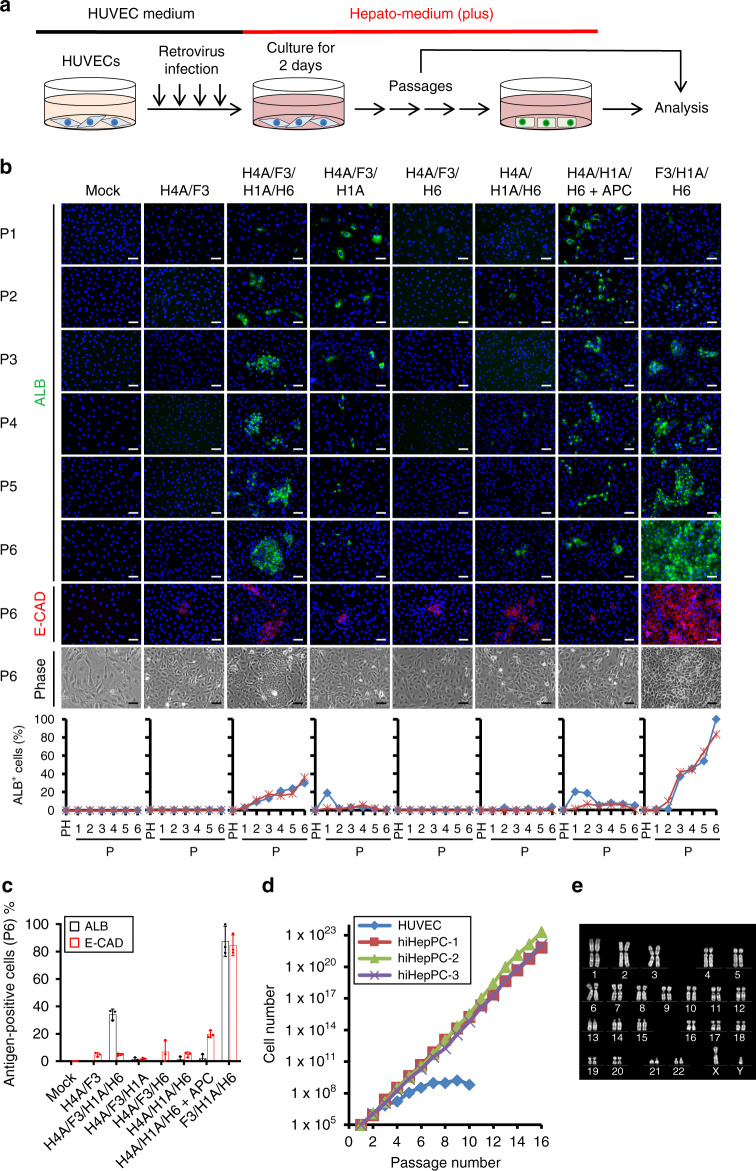
Generation of expandable hiHepPCs from HUVECs. **a** Schematic diagram of the experimental procedure. **b** Immunofluorescence staining of ALB or E-CAD was conducted for mock-infected HUVECs and HUVECs transduced with the indicated factors at the indicated passage numbers (P). HNF4A, FOXA3, HNF1A, and HNF6 are abbreviated as H4A, F3, H1A, and H6, respectively. Representative fluorescence images and morphologies of mock-infected and transduced HUVECs are shown. DNA was stained with DAPI. Scale bars, 50 µm. The graphs show the percentages of ALB^+^ cells observed in individual cultures during passaging. The data obtained from two independent experiments are indicated by red and blue lines in the graphs, respectively. PH parental HUVEC. **c** The percentages of cells immunoreactive for ALB or E-CAD among mock-infected HUVECs and HUVECs transduced with the indicated factors at passage (P) 6. Data represent the mean ± SD (*n* = 3 independent experiments). **d** Growth curves of mock-infected HUVECs and three different hiHepPCs generated by transducing HUVECs with *FOXA3*, *HNF1A*, and *HNF6* in three independent experiments. Cells (1 × 10^5^) were passaged every 7 days in wells of 6-well plates. **e** A representative image of a karyotype of a hiHepPC at passage 12. Note that the numbers of chromosomes in all 20 hiHepPCs analyzed in this study were normal. Source data are provided as a Source Data file.

For the direct reprogramming of HUVECs into hiHepPCs, we first selected a set of two genes, *HNF4A* and *FOXA3*, which we have previously identified as the essential factors for inducing the conversion of mouse fibroblasts into iHepCs^4^. To examine the potential of these two genes, HUVECs were infected with retroviruses expressing each gene and subjected to serial passages to determine whether HUVECs could be converted to albumin^+^ (ALB^+^) hepatic lineage cells (Fig. 1a). However, the combinatorial expression of these two genes was not sufficient to induce either hiHepCs or hiHepPCs from HUVECs (Fig. 1b). Thus we then referred to previous studies in which two sets of genes, *HNF4A*, *FOXA3*, and *HNF1A* or *HNF4A*, *HNF1A*, and *HNF6*, were used to induce hiHepCs from fibroblasts^5,6^. The results from these two studies indicate that HNF4A, FOXA3, HNF1A, and HNF6 are potential candidates for core regulators of inducing the hepatic fate in non-hepatic cells. Thus we selected these four TFs and determined whether the forced expression of *HNF4A*, *FOXA3*, *HNF1A*, and *HNF6* in HUVECs induced conversion to hiHepPCs. Our data showed that ALB^+^ cells appeared in HUVEC cultures after transduction with the four genes, and the percentage of ALB^+^ cells gradually increased during passaging, suggesting the generation of hiHepPCs (Fig. 1b). However, the percentage of ALB^+^ cells remaining at 30–40% at passage 6 after transduction suggested the presence of unnecessary factors among the four. Thus we determined the essential factors among the four by examining the effects of withdrawing individual factors from the pool of the four factors. Moreover, we added *ATF5*, *PROX1*, and *Cebpa* (designated *APC*) to the set of *HNF4A*, *HNF1A*, and *HNF6* for promoting hepatocyte differentiation, as described previously^5^. Our data showed that, in the absence of *HNF1A*, no ALB^+^ cells were induced from HUVECs, indicating that *HNF1A* is critical for inducing the hepatic program in HUVECs (Fig. 1b). Consistent with previous studies^5,6^, ALB^+^ cells induced from HUVECs after transduction with three sets of genes, *HNF4A*, *FOXA3*, and *HNF1A*; *HNF4A*, *HNF1A*, and *HNF6*; and *HNF4A*, *HNF1A*, and *HNF6* plus *APC*, did not proliferate during passaging (Fig. 1b). However, the proliferation of ALB^+^ cells was only observed when the viral pool lacked *HNF4A*, and the percentage of ALB^+^ cells gradually increased during passaging and reached 80–100% at passage 6 after transduction (Fig. 1b). Further withdrawal of individual factors from the pool of three defined factors had no effect on the induction of hiHepPCs (Supplementary Fig. 1a, b). Moreover, two sets of two TFs, FOXA3 with HNF1B or HNF1A, previously reported as defined factors for inducing hepatic stem/progenitor cells from mouse fibroblasts^11,12^, did not induce hiHepPCs from HUVECs (Supplementary Fig. 1a, b).

hiHepPCs generated by transducing HUVECs with *FOXA3*, *HNF1A*, and *HNF6* had the typical morphology of epithelial cells and expressed the epithelial cell marker E-cadherin (E-CAD), in addition to ALB (Fig. 1b, c). The expression of endodermal progenitor cell markers, such as *SOX17* and *C-X-C chemokine receptor type 4* (*CXCR4*), were downregulated following the conversion of HUVECs into hiHepPCs, suggesting that hiHepPCs are distinct from endodermal progenitor cells (Supplementary Fig. 1c). hiHepPCs were maintained in long-term culture with proliferation and showed normal karyotypes (Fig. 1d, e). Comparative genomic hybridization (CGH) analyses revealed that three of four different hiHepPCs have no chromosomal aberrations compared with the parental HUVECs, while only a deletion was found on chromosome 3 in one of the four hiHepPCs (Supplementary Fig. 1d). Although hiHepPCs can propagate in culture, they do not express *telomerase reverse transcriptase* (*TERT*) and show senescence-like phenotypes and reduced growth activity around passage 24 (Supplementary Fig. 1e–h). These characteristics are different from those of human hepatocellular carcinoma cells. Although *HNF4A* was not included in the reprogramming cocktail, endogenous *HNF4A* expression was induced in hiHepPCs (Supplementary Fig. 1i). Taken together, we identified a specific combination of three TFs, FOXA3, HNF1A, and HNF6, that enable the generation of expandable and genome-stable hiHepPCs from HUVECs.

### hiHepPCs expand in culture with hepatocyte production

**Fig. 2:**
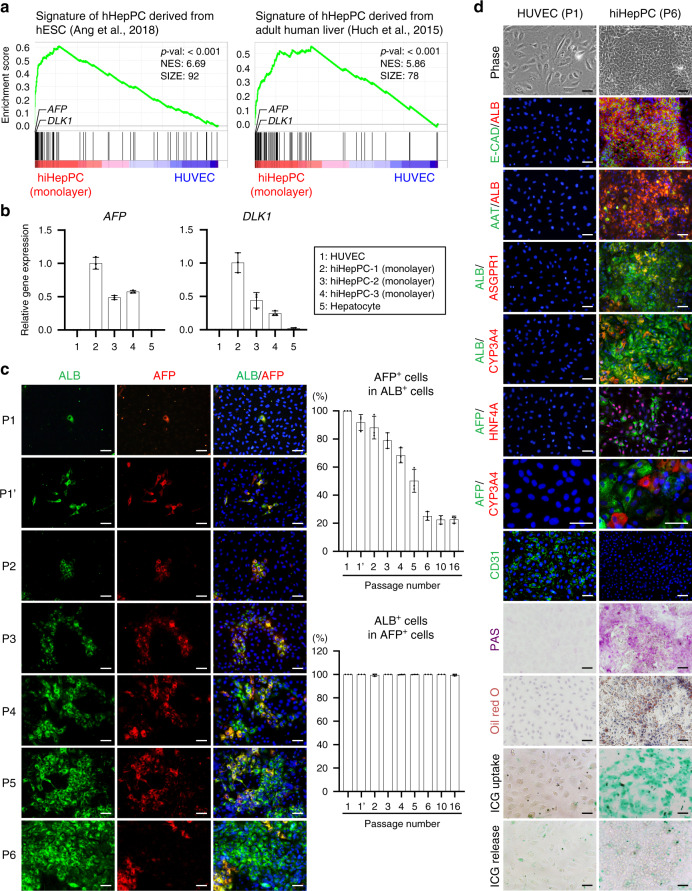
hiHepPCs are directly induced from HUVECs and differentiate into hepatocytes. **a** GSEA of CEL-seq2 data for hiHepPC monolayer cultures at passage 8 and HUVECs was performed using the set of top 100 genes specifically upregulated in hHepPCs derived from hESCs^13^ or adult human livers^14^. **b** qPCR analyses of hHepPC marker genes were performed on total RNA obtained from HUVECs, three different hiHepPCs at passage 8 in monolayer culture, and human hepatocytes. All data were normalized with the values for hiHepPC-1 (monolayer), and the fold differences are shown. Data represent the mean ± SD (*n* = 3 independent assays). **c** Co-immunofluorescence staining of ALB with AFP was conducted for hiHepPCs induced from HUVECs at the indicated passage numbers (P). P1 and P1’ designate days 4 and 7, respectively, after the initial passage of transduced HUVECs. The upper and lower right graphs show the percentages of AFP^+^ cells in ALB^+^ cells and ALB^+^ cells in AFP^+^ cells, respectively, which were observed in individual cultures of hiHepPCs during passaging. Data represent the mean ± SD (*n* = 3 independent experiments). **d** Co-immunofluorescence staining of ALB with E-CAD, AAT, ASGPR1, or CYP3A4 and of AFP with HNF4A or CYP3A4 and immunofluorescence staining of CD31 were conducted for mock-infected HUVECs at passage (P) 1 and hiHepPC monolayer cultures at P6. Periodic acid–Schiff (PAS) staining and oil red O staining were also conducted for mock-infected HUVECs and hiHepPC monolayer cultures to visualize glycogen stores and lipid synthesis, respectively, in hiHepPC-derived hepatocytes. Additionally, ICG uptake and subsequent release by hiHepPC-derived hepatocytes are shown. DNA was stained with DAPI. Scale bars, 50 µm. Source data are provided as a Source Data file.

To assess similarities between hiHepPCs and hHepPCs, we investigated the global gene expression profiles in hiHepPCs using CEL-seq2, a method of RNA sequencing (RNA-seq), and performed gene set enrichment analysis (GSEA) using a publicized set of genes specifically upregulated in hHepPCs derived from human embryonic stem cells^13^ or adult human livers^14^. The data showed that the expression of hHepPC-specific genes, including *α-fetoprotein* (*AFP*) and *delta-like 1 homolog* (*DLK1*), was highly enriched in hiHepPC monolayer cultures (Fig. 2a and Supplementary Fig. 2a). Quantitative polymerase chain reaction (qPCR) analyses also revealed that, unlike HUVECs and adult human hepatocytes, hiHepPCs expressed *AFP* and *DLK1* (Fig. 2b). Moreover, in the monolayer hiHepPC cultures, AFP^+^ cells had a higher proliferative activity than AFP^−^ cells (Supplementary Fig. 2b). These results suggest that hiHepPCs with the features of hHepPCs are directly induced from HUVECs after transduction with *FOXA3*, *HNF1A*, and *HNF6* and subsequently propagate in culture. To investigate details, we conducted co-immunofluorescence staining of ALB with AFP or DLK1 for HUVEC cultures after transduction with the three genes. After the initial passage of transduced HUVECs, ALB^+^ cells were first observed at day 4 (Fig. 2c). At that time, 100% of ALB^+^ cells co-expressed AFP and DLK1, and the percentages of AFP^+^ cells and DLK1^+^ cells in ALB^+^ cells gradually decreased during passaging (Fig. 2c and Supplementary Fig. 2c). In the cultures of transduced HUVECs, the percentages of Ki67^+^ proliferating cells in the reprogrammed ALB^+^ cells were significantly higher than those in the non-reprogrammed ALB^−^ cells (Supplementary Fig. 2d). These results demonstrate that HUVECs can be directly converted into AFP^+^ DLK1^+^ ALB^+^ hiHepPCs after transduction and that hiHepPCs subsequently proliferate and give rise to AFP^−^DLK1^−^ALB^+^ hepatocytes as a descendant. Moreover, because almost all AFP^+^ cells co-express ALB (Fig. 2c), hiHepPC cultures are composed of at least two distinct types of hepatic lineage cells, such as AFP^+^ ALB^+^ hiHepPCs and AFP^−^ALB^+^ hepatocytes derived from hiHepPCs. The percentage of AFP^+^ ALB^+^ hiHepPCs became 25% at passage 6 after transduction, which was maintained even in late passages (Fig. 2c). Spontaneous differentiation of hiHepPCs to hepatocytes suggested that our culture condition was not suitable for stable expansion of hiHepPCs. As reported^15^, the inhibitors of transforming growth factor (TGF)-β signaling pathway, which are contained in our culture medium, may promote hepatocyte differentiation from hepatic progenitor cells. However, our data demonstrated that the TGF-β inhibitors did not affect the percentage of AFP^+^ ALB^+^ hiHepPCs in culture but were effective in the induction of hiHepPCs from HUVECs (Supplementary Fig. 2e, f).

A part of the hiHepPC-derived hepatocytes expresses not only ALB and E-CAD but also markers of adult hepatocytes, such as α1-antitrypsin (AAT), asialoglycoprotein receptor 1 (ASGPR1), and cytochrome P450 (CYP) 3A4 (Fig. 2d and Supplementary Fig. 2g). In addition, hiHepPC-derived hepatocytes can synthesize and store abundant glycogen and lipids in their cytoplasm and incorporate and excrete indocyanine green (ICG) (Fig. 2d). After transduction of HUVECs and subsequent generation of hiHepPCs, the endothelial cell marker CD31, which is expressed in HUVECs, was not detected in hiHepPCs (Fig. 2d). Gene ontology enrichment analysis (GOEA) also revealed that the expression of vascular endothelial cell-related genes was significantly downregulated after the reprogramming of HUVECs to hiHepPCs (Supplementary Fig. 2h). AFP^+^ cells maintained in hiHepPC cultures can be distinguished from CYP3A4^+^ hepatocytes (Fig. 2d). These CYP3A4^+^ hepatocytes first appeared at passage 4 in hiHepPC cultures and co-expressed ALB (Supplementary Fig. 3a, b). Mathematically, 75% of cells in hiHepPC cultures at passage 6 are characterized as AFP^−^ALB^+^ hepatocytes (Fig. 2c). However, AFP^−^ALB^+^ CYP3A4^+^ hepatocytes comprise only 15% under the same conditions (Supplementary Fig. 3b). Thus 60% of hiHepPC cultures likely consist of AFP^−^ALB^+^ CYP3A4^−^ differentiating hepatocytes, in addition to hiHepPCs and hiHepPC-derived hepatocytes. In mouse livers, Afp^−^Alb^+^ cells that do not express Cyp3a11 and Cyp3a13 (mouse homologs to human CYP3A4) are found in late-stage liver development (Supplementary Fig. 4), suggesting that hiHepPC-derived AFP^−^ALB^+^ CYP3A4^−^ cells can be considered as cells with features intermediate between hiHepPCs and hiHepPC-derived hepatocytes.

### Functional hepatocytes are generated in hiHepPC aggregates

**Fig. 3:**
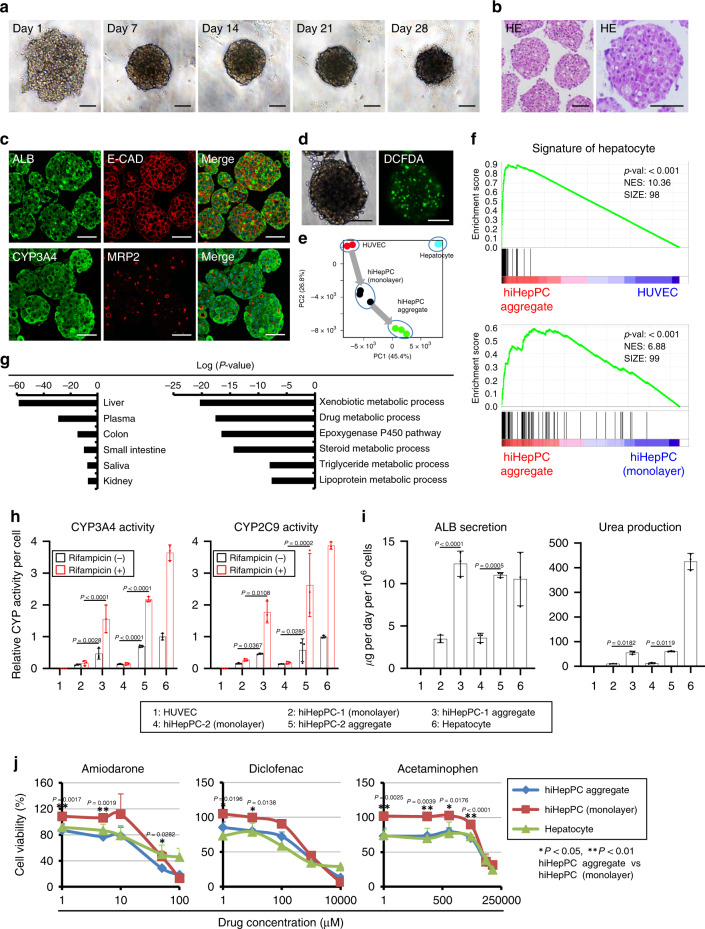
Promotion of hepatocyte differentiation in hiHepPC aggregates. **a** Representative morphologies of hiHepPC aggregates at days 1, 7, 14, 21, and 28 after initiation of 3D culture. **b** Representative images of hematoxylin and eosin (HE)-stained hiHepPC aggregates at day 7 after initiation of 3D culture. **c** Co-immunofluorescence staining of ALB with E-CAD and of CYP3A4 with MRP2 were conducted for hiHepPC aggregates at day 7 after initiation of 3D culture. DNA was stained with DAPI. **d** Representative bright-field and fluorescence images of a living hiHepPC aggregate at day 7 after initiation of 3D culture stained with 5- and 6-carboxy-2′,7′-dichlorofluorescein diacetate (carboxy-DCFDA). Green fluorescence shows functional MRP2 transporter activity in hiHepPC aggregates. **e** PCA was performed using CEL-seq2 data for HUVECs, hiHepPCs in monolayer and cell aggregation cultures, and human hepatocytes. **f** GSEA of CEL-seq2 data for hiHepPC aggregates and HUVECs and of those for hiHepPC aggregates and hiHepPC monolayer cultures were performed using the set of top 100 genes specifically upregulated in human hepatocytes. **g** GOEA was performed for genes with expression levels higher in hiHepPC aggregates than in hiHepPC monolayer cultures (left graph). The liver-enriched genes shown in the left graph were related to hepatic functions associated with xenobiotic metabolism, drug metabolism, and lipid metabolism (right graph). **h**, **i** CYP3A4 and CYP2C9 activities (**h**) and the amounts of ALB and urea in the culture media (**i**) were measured after culture of HUVECs, two different hiHepPCs, cell aggregates derived from these hiHepPCs, and human hepatocytes. All data shown in **h** were normalized with the values for hepatocytes in culture without rifampicin, and the fold differences are shown. **j** The viability of hiHepPCs in monolayer and cell aggregation cultures and primary human hepatocytes was measured after culture of cells with the indicated hepatotoxins. Statistical difference was determined by one-way analysis of variance followed by Tukey–Kramer test. Data represent the mean ± SD (*n* = 3 (**h**, **i**) or *n* = 4 (**j**) independent assays). Scale bars, 50 µm. Source data are provided as a Source Data file.

To induce further the differentiation of hiHepPCs into hepatocytes, we conducted cell aggregation culture of hiHepPCs. It is known that cell-aggregate formation facilitates the maturation of hPSC-derived hepatocyte-like cells^16^ and mouse iHepCs^17^. Under 3D culture conditions, about 95% of the plated hiHepPCs contributed to the formation of uniform cell aggregates, and these aggregates were maintained in culture without active induction of apoptosis for at least 4 weeks (Fig. 3a, b and Supplementary Fig. 5a, b). hiHepPC aggregates were composed of cells expressing ALB, E-CAD, CYP3A4, transferrin, AAT, and HNF4A (Fig. 3c and Supplementary Fig. 5c), and bile canaliculi-like structures were formed by cells expressing the canalicular membrane protein multidrug resistance-associated protein 2 (MRP2) and having functional MRP2 transporter activity in the aggregates (Fig. 3c, d). Cell counting and flow cytometric analyses revealed that ALB, CYP3A4, and ASGPR1 were expressed by more than 95% of cells in hiHepPC aggregates (Supplementary Fig. 5d, e). These data suggested that the differentiation of hiHepPCs into mature hepatocytes was promoted after cell-aggregate formation. Thus, to assess the hepatic maturation of hiHepPCs in cell aggregation culture, we investigated the global gene expression profiles in hiHepPC aggregates and performed principal component analysis (PCA), GSEA, and GOEA. PCA revealed that the gene expression signatures of hiHepPC monolayer cultures and hiHepPC aggregates were stepwisely close to that of human hepatocytes, indicating that hepatic maturation of hiHepPCs was promoted by forming cell aggregates (Fig. 3e). Indeed, GSEA showed that the expression of human hepatocyte-specific genes was significantly upregulated in hiHepPC aggregates compared with HUVECs and hiHepPC monolayer cultures (Fig. 3f and Supplementary Fig. 6a). Moreover, GOEA revealed that the extracted genes whose expression levels were higher in hiHepPC aggregates than in hiHepPC monolayer cultures were significantly enriched in genes expressed in the liver, and these liver-enriched genes were related to hepatic functions associated with xenobiotic metabolism, drug metabolism, and lipid metabolism (Fig. 3g). Meanwhile, genes with lower expression levels in hiHepPC aggregates than in hiHepPC monolayer cultures were enriched in genes encoding cell cycle-related proteins, which is consistent with the data showing a decrease in the number of Ki67^+^ proliferating cells in hiHepPC aggregates (Supplementary Fig. 6b, c). These findings demonstrate that cell-aggregate formation facilitates differentiation of hiHepPCs into growth-arrested functional hepatocytes. However, a part of genes contained in the GO terms associated with hepatic functions, including the lipoprotein and cholesterol metabolic processes, was not significantly upregulated in hiHepPC aggregates compared with human hepatocytes (Supplementary Fig. 6d). In contrast, there are also genes expressed higher in cells composing the hiHepPC aggregates than in human hepatocytes (Supplementary Fig. 6e). Thus it is suggested that additional stimuli are required for inducing more appropriate differentiation of hiHepPCs.

qPCR analyses showed that the expression of genes associated with hepatic functions, such as *CYP1A2*, *CYP2B6*, *CYP2C9*, *CYP2C19*, *CYP2E1*, *CYP3A4*, *uridine diphosphate glucuronosyltransferase* (*UGT*) *1A1*, *UGT1A6*, *UGT2B4*, *UGT2B15*, *ATP-binding cassette* (*ABC*) *B1*, *ABCC2*, and *ABCG2*, and hepatic immature phenotype, such as *AFP* and *DLK1*, were upregulated and downregulated, respectively, after the formation of hiHepPC aggregates (Supplementary Fig. 7a, b). The expression levels of cholangiocyte markers, such as *cytokeratin* (*CK*) *19*, *epithelial cell adhesion molecule* (*EpCAM*), and *cystic fibrosis transmembrane conductance regulator* (*CFTR*), that were low in the monolayer hiHepPC cultures slightly decreased in hiHepPC aggregates (Supplementary Fig. 7b). *HNF4A* expression induced in hiHepPCs was upregulated after cell-aggregate formation, suggesting a correlation between the expression level of *HNF4A* and differentiation of hiHepPCs into hepatocytes (Supplementary Fig. 7c). Meanwhile, there was little or no expression of intestinal epithelial cell marker genes, including a gene encoding the intestinal master TF CDX2, in both hiHepPC monolayer cultures and hiHepPC aggregates (Supplementary Fig. 7d–f), which excludes a possibility that intestinal epithelial cells are differentiated from hiHepPCs and express genes encoding enzymes produced in both the liver and intestine.

In cell aggregation culture, the activities of CYP3A4 and CYP2C9 in hiHepPCs were markedly increased compared with those in the monolayer hiHepPC cultures, and hiHepPC-derived hepatocytes in the aggregates could efficiently respond to rifampicin and potentiate the CYP activities, similar to primary human hepatocytes (Fig. 3h). Cell-aggregate formation also increased the levels of ALB secretion and urea production by hiHepPC-derived hepatocytes (Fig. 3i). Moreover, hepatotoxins, such as amiodarone, diclofenac, and acetaminophen, affect hiHepPC-derived hepatocytes in the aggregates and induce cell death more effectively and more similarly to primary human hepatocytes than hiHepPC monolayer cultures (Fig. 3j). Thus hiHepPC-derived hepatocytes composing cell aggregates have the specific functional features of mature hepatocytes. To compare the gene expression signature of hiHepPC aggregates with those of previously reported hiHepCs, we conducted GSEA using sets of genes specifically upregulated in hiHepCs generated by Du and colleagues^5^, designated hiHepC-Du, and hiHepCs and hiHepCs expressing SV40 large T antigen (hiHepC-LT) generated by Huang and colleagues^6^, designated hiHepC/hiHepC-LT-Huang. The data showed that the expression of hiHepC-Du-specific genes and hiHepC/hiHepC-LT-Huang-specific genes were highly and poorly enriched in hiHepPC aggregates, respectively (Supplementary Fig. 8a). In addition, the expression of genes specifically upregulated in hiHepPC aggregates and hiHepC-Du were significantly enriched in human hepatocytes (Supplementary Fig. 8b). Thus the gene expression signature of hiHepPC aggregates is relatively similar to those of hiHepC-Du and human hepatocytes, rather than those of hiHepC/hiHepC-LT-Huang. Taken together, our findings demonstrate that cell-aggregate formation can induce hiHepPCs to differentiate into functional hepatocytes and undergo growth arrest.

### hiHepPCs differentiate into cholangiocytes in 3D culture

**Fig. 4:**
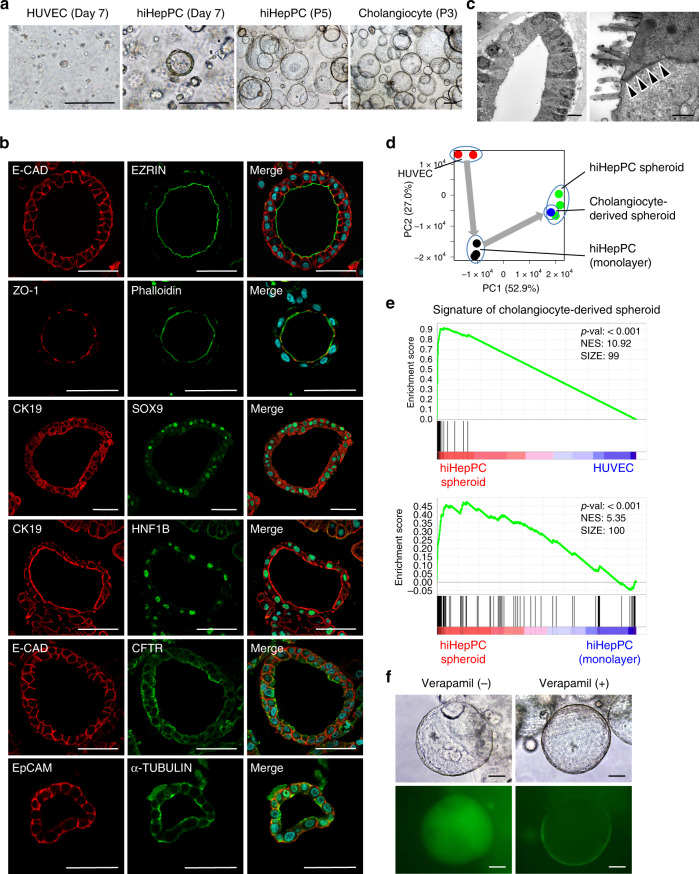
Cystic spheroid formation from hiHepPC-derived cholangiocytes. **a** Representative images of 3D cultures of HUVECs and hiHepPCs at day 7 and passage (P) 5 and those of adult human liver-derived cholangiocytes at P3 after initiation of 3D culture with Matrigel. Scale bars, 100 µm. **b** Co-immunofluorescence staining of E-CAD with EZRIN or CFTR, of CK19 with SOX9 or HNF1B, and of EpCAM with α-TUBULIN and immunofluorescence staining of ZO-1 with phalloidin staining of F-actin were conducted for hiHepPC spheroids. DNA was stained with DAPI. Scale bars, 50 µm. **c** Representative ultrastructural images of hiHepPC spheroids (*n* = 10 spheroids). Black arrowheads indicate intracellular tight junctional complexes. Scale bars, 10 µm (left panel) and 500 nm (right panel). **d** PCA was performed using CEL-seq2 data for HUVECs, hiHepPC monolayer cultures, hiHepPC spheroids, and human fetal cholangiocyte-derived spheroids. **e** GSEA of CEL-seq2 data for hiHepPC spheroids and HUVECs and of those for hiHepPC spheroids and hiHepPC monolayer cultures were performed using the set of top 100 genes specifically upregulated in human fetal cholangiocyte-derived spheroids. **f** Representative bright-field and fluorescence images showing uptake of rhodamine 123 (green) into the luminal space of hiHepPC spheroids and inhibition of rhodamine 123 transport after treating the spheroids with verapamil before the addition of rhodamine 123. Scale bars, 50 µm.

hHepPCs are generally defined as cells capable of differentiating into both hepatocytes and cholangiocytes. Thus we next determined whether hiHepPCs could differentiate into cholangiocytes, in addition to hepatocytes, by inoculating hiHepPCs into Matrigel and subjecting to 3D culture. At day 7 after initiation of 3D culture, about 0.5% of hiHepPCs gave rise to cells that formed spheroids, while HUVECs did not form any spheroids (Fig. 4a). These hiHepPC spheroids could stably expand and be maintained in long-term 3D culture by serial passaging, similar to human fetal cholangiocyte-derived spheroids, without karyotypic aberrations (Fig. 4a and Supplementary Fig. 9a). Immunofluorescence analyses revealed that the cells composing the hiHepPC spheroids consistently expressed E-CAD at the basolateral membrane, EZRIN at the apical surface, and the tight junction marker ZO-1 at the intercellular contact sites close to the apical surface, indicating that these spheroids are formed by epithelial cells maintaining distinctive apicobasal cell polarity (Fig. 4b). Additionally, apical actin accumulation shown by phalloidin staining indicated the epithelial cell polarity of spheroid-forming cells (Fig. 4b). Moreover, transmission electron microscopy revealed that hiHepPC spheroids consisted of cells with epithelial morphology and polarity that lined the central lumen, attached to adjacent cells by intracellular tight junctional complexes, and were decorated with microvilli (Fig. 4c). These spheroid-forming epithelial cells expressed cholangiocyte markers, such as CK19, SOX9, HNF1B, CFTR, EpCAM, and α-TUBULIN (Fig. 4b). qPCR analyses also revealed that the expression levels of cholangiocyte markers (*CK19*, *HNF1B*, *SOX9*, *CFTR*, *secretin receptor* (*SCTR*), and *EpCAM*) and hHepPC markers (*ALB* and *AFP*) increased and decreased, respectively, in the hiHepPC spheroids compared with those in the monolayer hiHepPC cultures (Supplementary Fig. 9b). In addition, PCA showed that hiHepPC spheroids and human fetal cholangiocyte-derived spheroids occupy a similar dimensional space, indicating a close resemblance between the gene expression signatures of two different types of spheroids (Fig. 4d). In fact, GSEA revealed that the expression of genes specifically upregulated in human fetal cholangiocyte-derived spheroids was highly enriched in the hiHepPC spheroids compared with HUVECs and hiHepPC monolayer cultures (Fig. 4e and Supplementary Fig. 9c). However, GOEA revealed that there are also genes expressed differentially between hiHepPC spheroids and human fetal cholangiocyte-derived spheroids (Supplementary Fig. 9d, e).

To determine whether epithelial cells composing hiHepPC spheroids have functional features of cholangiocytes, we evaluated the potential for incorporating rhodamine 123 into the luminal space of the spheroids. Rhodamine 123 is generally used to determine the transporter activity of multidrug resistance 1 (MDR1) in cholangiocytes. Our data showed that rhodamine 123 was successfully transported into the luminal space of the spheroids, and this transporter activity was blocked by addition of the MDR1 inhibitor verapamil (Fig. 4f). Thus epithelial cells constituting hiHepPC spheroids can transport rhodamine 123 through MDR1, similar to cholangiocytes. Moreover, we also conducted a forskolin (FSK)-induced swelling assay to determine whether CFTR functioned in hiHepPC spheroids. Because FSK was usually present in our culture medium, we first removed FSK from the culture medium and subsequently added it for analyses. Our data showed that addition of FSK with or without the CFTR inhibitor CFTRinh-172 to the culture media blocked or induced the swelling of hiHepPC spheroids, respectively (Supplementary Fig. 10). Thus epithelial cells forming hiHepPC spheroids can respond to FSK in a CFTR-dependent manner and increase the size of the spheroids, which is a functional feature of cholangiocytes. Taken together, our findings demonstrate that hiHepPCs can differentiate into cells with the morphological and functional properties of cholangiocytes that can form cystic epithelial spheroids resembling those formed by cholangiocytes under 3D culture conditions.

### Clonality and stable expansion of hiHepPCs

To ascertain the bi-lineage differentiation potential of hiHepPCs, we conducted clonal analyses of hiHepPCs. The cells in hiHepPC cultures generated in three different experiments were trypsinized, subjected to clone sorting by flow cytometry, and cultured under single-cell culture conditions (Supplementary Fig. 11a). About 10 and 2% of the sorted cells could form colonies and continuously proliferate over a prolonged culture period, respectively (Supplementary Fig. 11b). The hiHepPC clones propagating in monolayer culture maintained the expression of ALB, E-CAD, and CYP3A4 (Supplementary Fig. 11c) and formed cell aggregates and cystic epithelial spheroids composed of hepatocytes and cholangiocytes, respectively, in 3D culture (Supplementary Fig. 11d, e), similar to bulk cultures of hiHepPCs. Thus hiHepPCs can be defined as bipotential cells capable of giving rise to both hepatocytes and cholangiocytes from single cells.

The global gene expression profile in hiHepPCs did not change significantly after serial passages, and hiHepPCs propagating in late passages were able to produce hepatocytes and cholangiocytes under 3D culture conditions, indicating stability of hiHepPCs in long-term monolayer culture (Supplementary Fig. 12a, b). Exogenous gene expression of *FOXA3*, *HNF1A*, and *HNF6* were downregulated after the formation of hiHepPC aggregates and spheroids, suggesting promotion of retroviral gene silencing during differentiation of hiHepPCs in 3D culture (Supplementary Fig. 12c). Interestingly, hiHepPCs could be generated from HUVECs when we used FOXA1 or FOXA2, instead of FOXA3, with HNF1A and HNF6 as the reprogramming factors and differentiate into functional hepatocytes and epithelial cells forming cystic spheroids, indicating redundant functions of the FOXA family of TFs for induction of hiHepPCs (Supplementary Fig. 13).

### Liver tissue reconstitution by hiHepPC-derived cells

**Fig. 5:**
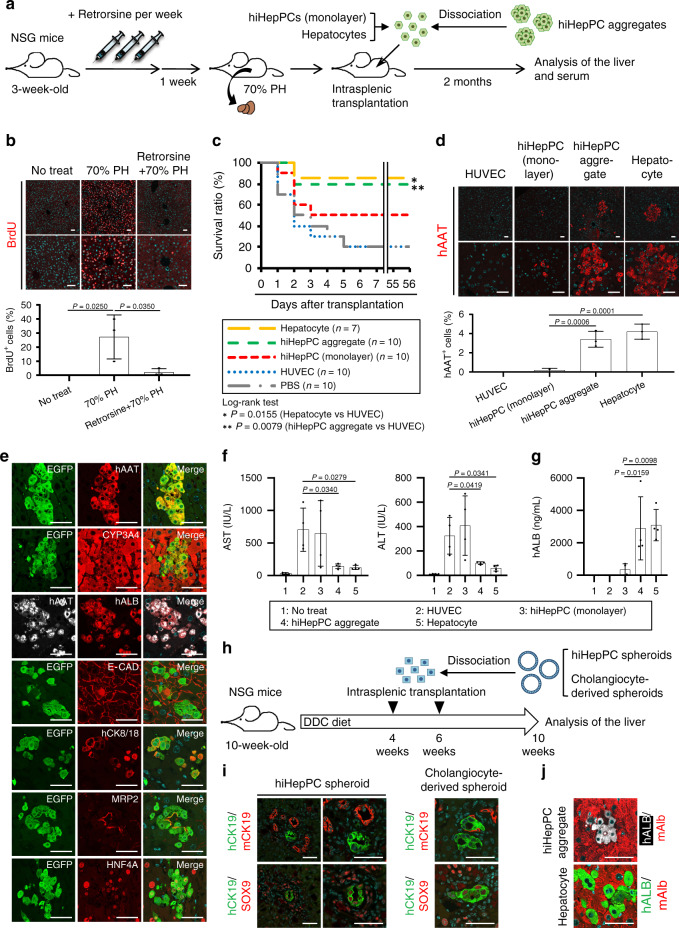
hiHepPC-derived cells functionally reconstitute hepatic tissues in vivo. **a** Schematic diagram of the experimental procedure. hiHepPCs were marked by infection with a virus expressing *enhanced green fluorescent protein* (*EGFP*) before transplantation. PH partial hepatectomy. **b** Immunofluorescence staining of the thymidine analog 5-bromo-2’-deoxyuridine (BrdU) was conducted for the livers of control mice (No treat) and retrorsine-treated and untreated mice at day 2 after PH. The graph shows the percentages of BrdU^+^ cells observed in individual mouse livers. **c** Kaplan–Meier survival curves of the recipient retrorsine-treated hepatectomized mice after intrasplenic injection of human hepatocytes, cells dissociated from hiHepPC aggregates and hiHepPC monolayer cultures, HUVECs, or only PBS into the liver. Statistical analyses using the log-rank test revealed significant differences between the curves for HUVECs and hiHepPC aggregates or hepatocytes but not between those for hiHepPC aggregates and hepatocytes (*P* = 0.309). **d** Immunofluorescence staining of human AAT (hAAT) was conducted for recipient mouse livers 2 months after transplantation. The graph shows the percentages of hAAT^+^ cells observed in individual mouse livers. **e** Co-immunofluorescence staining of EGFP with hAAT, CYP3A4, E-CAD, human CK8/18 (hCK8/18), MRP2, or HNF4A and of hAAT with human ALB (hALB) were conducted for recipient mouse livers 2 months after transplantation of cells dissociated from hiHepPC aggregates. **f**, **g** The amounts of AST and ALT (**f**) and those of hALB (**g**) in the serum of recipient mice were measured 2 days and 2 months after transplantation, respectively. The sera of control mice (No treat) were measured as negative controls. **h** Schematic diagram of the experimental procedure. **i** Co-immunofluorescence staining of human CK19 (hCK19) with mouse CK19 (mCK19) or SOX9 was conducted for DDC-treated mouse livers 4 weeks after the last injection of cells dissociated from hiHepPC spheroids or human fetal cholangiocyte-derived spheroids. **j** Co-immunofluorescence staining of hALB with mouse Alb (mAlb) was conducted for retrorsine-treated hepatectomized mouse livers 2 months after transplantation of cells dissociated from hiHepPC aggregates or human hepatocytes. Statistical difference was determined by one-way analysis of variance followed by Tukey–Kramer test (**b**) or Dunnett’s test (**d**, **f**, **g**). Data represent the mean ± SD (*n* = 3 (**b**, **d**) or 4 (**f**, **g**) independent experiments). DNA was stained with DAPI. Scale bars, 50 µm. Source data are provided as a Source Data file.

In addition to in vitro analyses, we examined the in vivo tissue-reconstitution potential in hiHepPC-derived hepatocytes and cholangiocytes. To investigate the reconstitution of liver parenchyma by hiHepPC-derived hepatocytes, we intrasplenically injected cells dissociated from hiHepPC monolayer cultures and hiHepPC aggregates into the livers of retrorsine-treated immunodeficient NOD/SCID/gamma (NSG) mice after 70% partial hepatectomy (PH) (Fig. 5a). Retrorsine is a known inhibitor of hepatocyte proliferation in rats^18^ and mice^19,20^. Consistent with these previous studies, our present data showed that PH-induced hepatocyte proliferation was significantly inhibited by pretreating mice with retrorsine (Fig. 5b). Thus, in this retrorsine/PH-induced acute liver failure model, donor cells can competitively contribute to liver regeneration after transplantation into the injured liver. Because retrorsine-treated hepatocytes were unable to efficiently regenerate the liver parenchyma after PH, only 20% of the retrorsine-treated hepatectomized mice could survive for >50 days after intrasplenic injection of phosphate-buffered saline (PBS) or HUVECs (Fig. 5c). However, survival curves revealed that 80% of the recipient mice transplanted with cells dissociated from hiHepPC aggregates were surviving from the second day after transplantation, similar to the mice transplanted with human hepatocytes (Fig. 5c). Similarly, transplantation of cells obtained from hiHepPC monolayer cultures improved survival ratios of the recipient mice, and 50% of mice eventually survived after transplantation (Fig. 5c).

Immunofluorescence staining of human AAT revealed that about 4% of hepatocytes in the livers of recipient mice were repopulated by both hiHepPC-derived hepatocytes isolated from the aggregates and human liver-derived hepatocytes 2 months after transplantation (Fig. 5d). These hiHepPC-derived hepatocytes engrafted in the liver expressed hepatocyte markers, such as CYP3A4, ALB, E-CAD, CK8/18, MRP2, and HNF4A, in addition to AAT (Fig. 5e). Transplantation of hiHepPC-derived hepatocytes dissociated from the aggregates and human hepatocytes immediately and significantly ameliorated liver failure, such as increases in aspartate aminotransferase (AST) and alanine transaminase (ALT), at day 2 after transplantation (Fig. 5f), and relatively large amounts of human ALB were detected in the serum of the recipient mice 2 months after transplantation (Fig. 5g).

To examine the potential for biliary ductal tissue reconstitution in hiHepPC-derived cholangiocytes, we intrasplenically injected cells dissociated from hiHepPC spheroids into the livers of NSG mice treated with 3,5-diethoxycarbonyl-1,4-dihydrocollidine (DDC; Fig. 5h). Four weeks after the last injection of cells, hiHepPC-derived cholangiocytes became engrafted in the liver and formed ductal tissues composed of epithelial cells expressing CK19 and SOX9, similar to cells obtained from human fetal cholangiocyte-derived spheroids (Fig. 5i and Supplementary Fig. 14a). The bile pigment bilirubin was accumulated in the luminal space of ductal tissues formed by transplanted hiHepPC-derived cholangiocytes, indicating a functional property of biliary ductal tissues (Supplementary Fig. 14b). Co-immunofluorescence staining of human ALB with mouse Alb and that of human CK19 with mouse CK19 revealed that no donor-derived cells expressed mouse Alb and CK19 after transplantation of hiHepPC-derived hepatocytes and cholangiocytes, human hepatocytes, and human cholangiocytes (Fig. 5i, j). Thus hiHepPC-derived hepatocytes and cholangiocytes have the potential to functionally reconstitute a part of liver parenchyma and biliary ductal tissues, respectively, without fusion with recipient mouse hepatocytes and cholangiocytes, after transplantation.

### Generation of hiHepPCs from HPBECs

**Fig. 6:**
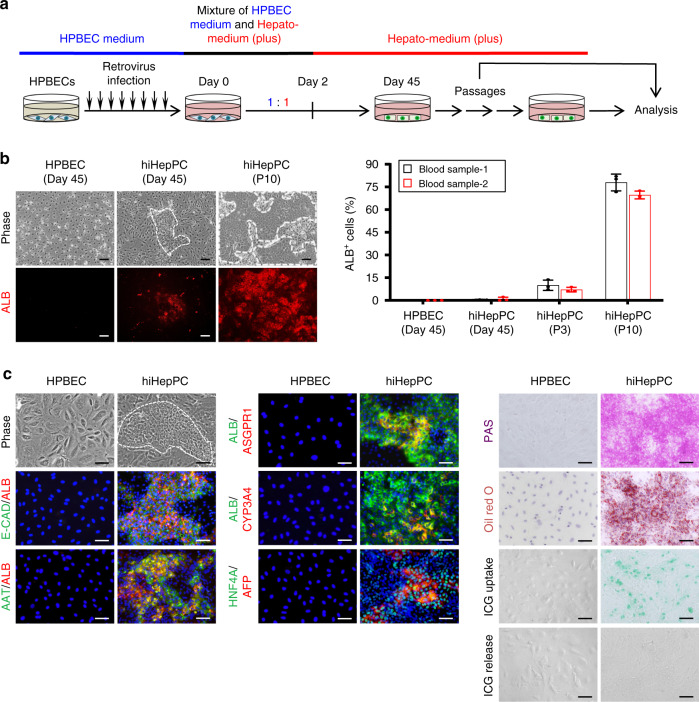
Direct conversion of HPBECs to hiHepPCs. **a** Schematic diagram of the experimental procedure. **b** Immunofluorescence staining of ALB was conducted for mock-infected HPBECs at day 45 and HPBEC-derived hiHepPCs at day 45 and passage (P) 10 after retrovirus infection. Representative morphologies and fluorescence images of these two types of cells are shown. White broken lines surround hiHepPC colonies. Scale bars, 100 µm. The graph shows the percentages of ALB^+^ cells observed in individual cultures of HPBECs at day 45 and hiHepPCs at day 45, P3, and P10 after retrovirus infection. The data obtained from two biologically independent experiments using different blood samples are shown in the graph. Data represent the mean ± SD (*n* = 3 independent assays). **c** Co-immunofluorescence staining of ALB with E-CAD, AAT, ASGPR1, or CYP3A4 and of AFP with HNF4A; PAS staining; and oil red O staining were conducted for HPBECs and HPBEC-derived hiHepPCs. Representative morphologies of these two types of cells are also shown. White broken line surrounds a hiHepPC colony. In addition, HPBEC-derived hiHepPCs incorporated and excreted ICG. DNA was stained with DAPI. Scale bars, 50 µm. Source data are provided as a Source Data file.

Finally, we sought to induce the conversion of HPBECs into hiHepPCs by forced expression of the defined TFs (Fig. 6a). Similar to the case of HUVECs described above, HPBECs infected with viruses expressing *FOXA3*, *HNF1A*, and *HNF6* could be converted into hiHepPCs (Fig. 6b). Although ALB^+^ cells in cultures of transduced HPBECs appeared and proliferated at a slower rate than those in cultures of transduced HUVECs, the percentage of ALB^+^ cells gradually increased during passaging and reached 65–75% at passage 10 after transduction (Fig. 6b). These HPBEC-derived hiHepPCs closely resembled HUVEC-derived hiHepPCs in terms of epithelial morphology and functional aspects, giving rise to cells expressing hepatocyte markers, such as AAT, ASGPR1, and CYP3A4, in addition to ALB and E-CAD, and possessing hepatocyte-specific functional features, including glycogen and lipid synthesis and ICG uptake and release (Fig. 6c). Moreover, similar to HUVEC-derived hiHepPCs, HPBEC-derived hiHepPCs expressed HNF4A and harbored a population of AFP^+^ cells during passaging in long-term monolayer culture (Fig. 6c).

**Fig. 7:**
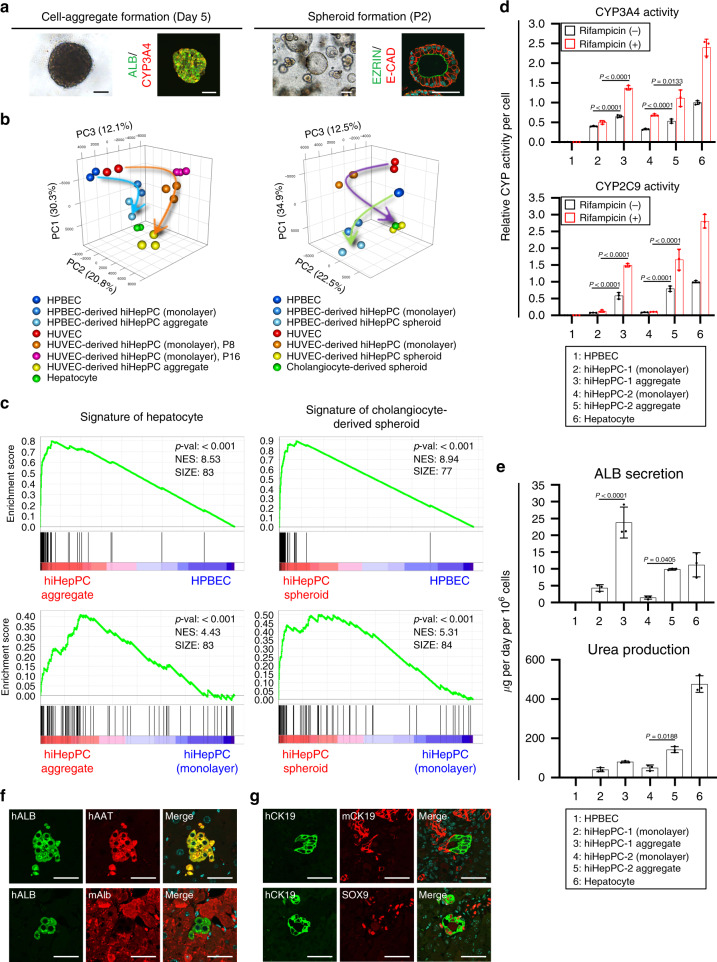
Bi-lineage differentiation potential in HPBEC-derived hiHepPCs. **a** HPBEC-derived hiHepPCs formed cell aggregates and cystic spheroids under 3D culture conditions. Co-immunofluorescence staining of ALB with CYP3A4 and of EZRIN with E-CAD were conducted for hiHepPC aggregates and spheroids, respectively. Representative morphologies and fluorescence images of hiHepPC aggregates at day 5 and hiHepPC spheroids at passage (P) 2 after initiation of 3D culture are shown. **b**, **c** We obtained CEL-seq2 data from HPBECs, HPBEC-derived hiHepPCs, and cell aggregates and spheroids derived from these hiHepPCs and performed PCA using CEL-seq2 data for the indicated cell types (**b**) and GSEA using the set of top 100 genes specifically upregulated in human hepatocytes or human fetal cholangiocyte-derived spheroids (**c**). P passage number of hiHepPCs in monolayer cultures. **d**, **e** CYP3A4 and CYP2C9 activities (**d**) and the amounts of ALB and urea in the culture media (**e**) were measured after culture of HPBECs, two different hiHepPCs induced from HPBECs, cell aggregates derived from these hiHepPCs, and human hepatocytes. All data shown in **d** were normalized with the values for hepatocytes in culture without rifampicin, and the fold differences are shown. Statistical difference was determined by one-way analysis of variance followed by Tukey–Kramer test. Data represent the mean ± SD (*n* = 3 independent assays). **f** Co-immunofluorescence staining of hALB with hAAT or mAlb was conducted for retrorsine-treated hepatectomized mouse livers 2 months after transplantation of cells dissociated from HPBEC-derived hiHepPC aggregates. **g** Co-immunofluorescence staining of hCK19 with mCK19 or SOX9 was conducted for DDC-treated mouse livers 4 weeks after the last injection of cells dissociated from HPBEC-derived hiHepPC spheroids. DNA was stained with DAPI. Scale bars, 50 µm. Source data are provided as a Source Data file.

Under 3D culture conditions, HPBEC-derived hiHepPCs were also able to form cell aggregates and cystic spheroids, in which hepatocytes and cholangiocytes were produced from hiHepPCs, respectively (Fig. 7a). PCA showed that, similar to HUVEC-derived hiHepPCs, HPBEC-derived hiHepPCs stepwisely acquired the gene expression signatures of human hepatocytes and cholangiocytes through differentiation in 3D culture (Fig. 7b). GSEA revealed that the expression of human hepatocyte- and cholangiocyte-specific genes was significantly upregulated in hiHepPC aggregates and spheroids, respectively, compared with HPBECs and hiHepPC monolayer cultures (Fig. 7c and Supplementary Fig. 15). Moreover, CYP3A4 and CYP2C9 activities and rifampicin responsiveness in the aggregates of HPBEC-derived hiHepPCs were much higher than those in the monolayer hiHepPC cultures (Fig. 7d), and hiHepPC-derived hepatocytes in the aggregates could efficiently secrete ALB and produce urea (Fig. 7e). In addition, HPBEC-derived hiHepPCs could give rise to hepatocytes and cholangiocytes capable of partially reconstituting the liver parenchyma and biliary ductal tissues, respectively, without cell fusion after transplantation into the injured mouse liver (Fig. 7f, g). Hepatocytes differentiated from HPBEC-derived hiHepPCs could repopulate about 2% of hepatocytes in the livers of recipient mice 2 months after transplantation, and 70% of mice eventually survived after transplantation. Thus, similar to HUVECs, HPBECs can be converted into expandable and bipotential hiHepPCs with a defined set of TFs.

The percentages of ALB^+^ cells in early passages of transduced HPBECs were much lower than those of transduced HUVECs. Thus, to increase the reprogramming efficiency, we used L-MYC in addition to the three defined factors. In mice, L-Myc is a factor capable of promoting the generation of induced PSCs with little transformation activity^21^. Our present data demonstrated that L-MYC was also effective in promoting the induction of ALB^+^ cells from both transduced HUVECs (Supplementary Fig. 16a–c, compared with Fig. 1b) and HPBECs (Supplementary Fig. 16d, compared with Fig. 6b), and the resulting HPBEC-derived hiHepPCs were designated MYC-hiHepPCs. These MYC-hiHepPCs have epithelial morphology, produce cells expressing hepatocyte markers, synthesizing and storing glycogen and lipids in their cytoplasm, and incorporating and excreting ICG, maintain AFP^+^ cells during passaging, and form cystic spheroids in 3D culture (Supplementary Fig. 16e). Clonal analyses of MYC-hiHepPCs cultured under single-cell culture conditions confirmed their bi-lineage differentiation potential into hepatocytes and cholangiocytes (Supplementary Fig. 17). Similar to HUVEC-derived hiHepPCs, HPBEC-derived MYC-hiHepPCs and hiHepPCs could also be maintained in long-term culture with proliferation and showed normal karyotypes (Supplementary Fig. 18a), while being negative for *TERT* expression and senescent around passage 24 (Supplementary Fig. 18b). Indeed, both types of HPBEC-derived hiHepPCs did not form any tumors after subcutaneous injection into immunodeficient NOD/SCID mice, as well as HUVEC-derived hiHepPCs (Supplementary Fig. 18c). Thus L-MYC can be used for efficient induction of hiHepPCs from HPBECs.

## Discussion

In this study, we showed that the combined expression of three TFs is sufficient to convert HUVECs and HPBECs into cells with the properties of hHepPCs. The resulting hiHepPCs can be maintained in long-term culture by propagating without chromosomal instability and give rise to both functional hepatocytes and cholangiocytes as descendants, demonstrating self-renewal capacity and bi-lineage differentiation potential in hiHepPCs. Moreover, hiHepPC-derived hepatocytes and cholangiocytes can functionally reconstitute liver tissues after transplantation into the injured mouse liver, similar to hepatocytes and cholangiocytes isolated from human livers. In addition, we identified L-MYC as a reprogramming-promoting factor for generating hiHepPCs. Overexpression of *L-MYC* with *FOXA3*, *HNF1A*, and *HNF6* in HUVECs and HPBECs increases the number of ALB^+^ cells in an early stage of the reprogramming, suggesting that L-MYC helps increase the efficiency of conversion of HUVECs and HPBECs into hiHepPCs, in addition to activating growth activity in hiHepPCs. Although HNF4A has been used as an essential factor for the induction of hiHepCs from fibroblasts^5,6^, the enforced expression of *HNF4A* was not required and even became harmful for the reprogramming of HUVECs into hiHepPCs. Because endogenous *HNF4A* expression was induced in hiHepPCs, a suitable expression level of *HNF4A* might be important for maintaining the features of hiHepPCs, while high expression of *HNF4A* might lead to further differentiation into growth-arrested hepatocytes. Indeed, *HNF4A* expression was upregulated after the induction of hiHepPC differentiation into growth-arrested functional hepatocytes in cell aggregation culture.

Hepatocyte differentiation is inefficiently but conventionally induced in hiHepPC monolayer cultures and strongly accelerated after formation of cell aggregates under 3D culture conditions. In contrast, cholangiocyte differentiation may be suppressed in the monolayer hiHepPC cultures, and spheroid-forming efficiency of hiHepPC-derived cholangiocytes is only 0.5%. Thus it is suggested that our culture condition is not suitable for cholangiocyte differentiation and/or spheroid formation from hiHepPCs. Indeed, similar to hiHepPCs, only 0.5–1% of human fetal liver-derived cholangiocytes that we obtained commercially and used in this study could form epithelial spheroids at the beginning of 3D culture. Thus, in future study, we should modify our culture condition to efficiently induce cholangiocyte differentiation and/or spheroid formation from hiHepPCs. A possible approach to induce differentiation of hiHepPCs into cholangiocytes is activation of Notch signaling in hiHepPCs, because hepatic stem/progenitor cells directly induced from mouse fibroblasts gave rise to cholangiocyte progenitor cells under stimulation of Notch signaling^12^. Nevertheless, our present data clearly demonstrated that hiHepPCs can give rise to both hepatocytes and cholangiocytes from single cells, indicating that hiHepPCs have bipotency.

Surprisingly, only 4% engraftment of transplanted hiHepPC-derived hepatocytes achieved 80% survival of recipient mice. Thus it is suggested that humoral factors secreted from hiHepPC-derived hepatocytes are effective to rescue the recipient mice after transplantation by activating cell-growth activity and protecting cells from cellular dysfunction. Meanwhile, similar to hiHepPC-derived hepatocytes, human liver-derived hepatocytes that we obtained commercially and used in this study also reconstituted only 4% of host livers after transplantation, and in both cases, 80% of recipient mice could survive. Thus it is suggested that, in the retrorsine/PH-induced acute liver failure model, many healthy hepatocytes are not required for rescuing recipient mice. Because retrorsine-treated hepatocytes in the recipient mouse liver do not completely stop proliferation, they could weakly contribute to liver regeneration after PH. Indeed, 20% of recipient mice could survive even after intrasplenic injection of control PBS or HUVECs. For the treatment of patients with acute liver failure, immediate therapeutic effects given by transplanted hepatocytes are required. Thus, to improve hepatic function of those patients immediately, functionally mature hepatocytes, rather than immature hepatocytes, should be transplanted into the liver of those patients. As shown in this study, hiHepPC-derived hepatocytes have functional properties similar to human liver-derived hepatocytes in vitro and in vivo. Thus a transplantation therapy using hiHepPC-derived hepatocytes would be developed to an effective method for treating patients with acute liver failure and other liver disorders.

The induction of hiHepPCs requires the overexpression of *FOXA3*, *HNF1A*, and *HNF6* in HUVECs and HPBECs, although *FOXA3* can be replaced with *FOXA1* or *FOXA2*. FOXA proteins are considered pioneer factors that can bind to condensed chromatin, enabling the relief of chromatin compaction and allowing access of other TFs to the enhancers of target genes to initiate tissue-specific gene expression^22–24^. Thus FOXA proteins allow binding of HNF1A and HNF6 to their specific target sites, leading to the induction of the program of hHepPCs in HUVECs and HPBECs. In mice, Hnf1α has been shown to play critical roles in the differentiation and function of hepatocytes^25^. However, Hnf6 has been shown to be essential for cholangiocyte differentiation and bile duct morphogenesis^26^. Thus, at least at present, it is difficult to explain why combined the expression of *FOXA3*, *HNF1A*, and *HNF6* can induce the fate of hHepPCs in endothelial cells. In a case of mice, Hnf6 is not required, but only two TFs, such as Foxa3 and Hnf1α, are sufficient for inducing hepatic stem/progenitor cells from fibroblasts^12^. Although it is not possible to induce hiHepPCs by using only FOXA3 and HNF1A, these two factors may have fundamental roles in the induction of the hepatic fate in non-hepatic cells, and thus the role of HNF6 in the induction of hiHepPCs is interesting and should be clarified in future study. Since biliary lineage cells in the injured adult mouse liver act as facultative stem/progenitor cells^27^, it is possible to speculate that the enforced expression of *HNF6* is involved in the acquisition of the immature phenotype in hiHepPCs.

Generating expandable and bipotential hiHepPCs using direct reprogramming technology may facilitate their application for drug development and therapeutic strategies for various liver diseases. Hepatocytes and cholangiocytes that can be continuously produced from hiHepPCs in long-term culture may be useful as an alternative to human liver-derived hepatocytes and cholangiocytes, respectively. In particular, because hiHepPCs can be generated from adult human peripheral blood, we could obtain hiHepPC-derived hepatocytes and cholangiocytes from, theoretically, many healthy humans, as well as many patients, by only collecting blood samples and use these cells for investigations of individual differences in the pharmacological effects and toxicities of drugs.

## Methods

### Mice

NSG (NOD.Cg-*Prkdc*^*scid*^*Il2rg*^*tm1Wjl*^/SzJ) male mice (3 and 10 weeks old), NOD/SCID female mice (10 weeks old) (both from Charles River Laboratories), C57BL/6 male mice (8 weeks old) (Clea), C57BL/6 neonatal mice (2 days old), and C57BL/6 mouse embryos (embryonic days 12.5 and 16.5) were used in this study. Mice were housed in groups of 2–4 per cage in a 12-h light/dark cycle (08:00–20:00 light; 20:00–08:00 dark), with controlled room temperature (22 ± 4 °C) and relative humidity (60%). The experiments were approved by the Kyushu University Animal Experiment Committee, and the care of the animals was in accordance with institutional guidelines.

### Cell lines

Plat-GP cells (Cell Biolabs) were cultured in Dulbecco’s modified Eagle’s medium (DMEM) (Nacalai Tesque) containing 10% fetal bovine serum (FBS) (GIBCO), 2 mM L-glutamine (Nacalai Tesque), and penicillin/streptomycin (Nacalai Tesque) and used to produce recombinant retroviruses^10^. The human hepatocellular carcinoma cell lines HepG2 (RCB1886) and HuH7 (RCB1942) were provided by the RIKEN BRC and cultured in DMEM containing 10% FBS, 2 mM L-glutamine, and penicillin/streptomycin.

### Cell culture

HUVECs (Takara) were cultured in HUVEC medium [1:1 mixture of Medium 200 (Thermo Fisher Scientific), supplemented with Low Serum Growth Supplement (Thermo Fisher Scientific), and FibroLife S2 Comp Kit (Kurabo)]. HPBECs were obtained from healthy donors as described previously^28,29^. Briefly, peripheral blood mononuclear cells were isolated from the blood samples of healthy donors using a Lymphoprep (STEMCELL Technologies) according to the manufacturer’s instructions, plated on type I collagen-coated 24-well plates (Iwaki), and cultured in HPBEC medium [EGM-2MV BulletKit (Lonza), supplemented with 10% FBS and 50% FibroLife S2 Comp Kit]. The collection of adult human peripheral blood was approved by the Kyushu University Ethics Committee and performed after obtaining informed consent. hiHepPCs were grown in hepato-medium (plus) composed of a 1:1 mixture of DMEM and F-12 (Nacalai Tesque), supplemented with 20% FibroLife S2 Comp Kit, 4% FBS, 1 µg mL^–1^ insulin (Wako), 10^–7^ M dexamethasone (Sigma-Aldrich), 10 mM nicotinamide (Sigma-Aldrich), 2 mM L-glutamine (Nacalai Tesque), 50 µM β-mercaptoethanol (Nacalai Tesque), 20 ng mL^–1^ recombinant human hepatocyte growth factor (rhHGF) (PeproTech), 1 µM A8301 (Tocris), 2 µM SB431542 (Tocris), 5 µM Y-27632 (Wako), and penicillin/streptomycin (Nacalai Tesque). hiHepPC aggregates were formed from 5 × 10^3^ hiHepPCs in each well of ultra-low attachment 96-well plates coated with poly 2-hydroxyethyl methacrylate (Sumitomo Bakelite) and maintained in medium composed of a 1:1 mixture of DMEM and F-12, supplemented with 1 µg mL^–1^ insulin, 10 mM nicotinamide, 2 mM L-glutamine, 50 µM β-mercaptoethanol, 20 ng mL^–1^ rhHGF, 1 µM FSK (Nacalai Tesque), 1 µM A8301, 20 µM Y-27632, and penicillin/streptomycin. For differentiation of hiHepPCs into cholangiocytes and for 3D culture of human fetal liver-derived cholangiocytes (ScienCell Research Laboratories), 1 × 10^5^ hiHepPCs and 1 × 10^5^ human cholangiocytes, respectively, were mixed with 50 µL of Matrigel (BD Biosciences) and plated on 24-well plates. After polymerization of the Matrigel at 37 °C, a medium for expansion of adult human bile duct-derived progenitor cells^14^ was added. Adult human liver-derived hepatocytes (Lonza) were cultured in medium (1:1 mixture of DMEM and F-12, supplemented with 4% FBS, 1 µg mL^–1^ insulin, 10^–7^ M dexamethasone, 10 mM nicotinamide, 2 mM L-glutamine, 50 µM β-mercaptoethanol, 20 ng mL^–1^ rhHGF, and penicillin/streptomycin). For single-cell culture analyses, cells identified by clone sorting using FACS Jazz (BD Biosciences) and BD FACS Sortware sorter software (BD Biosciences) were cultured in individual wells of type I collagen-coated 96-well plates, and the clonal colonies formed from each cell were expanded and analyzed.

### Retrovirus production and transduction of cells

Human *FOXA1*, *FOXA2*, *FOXA3*, *HNF1A*, *HNF1B*, *HNF4A*, *HNF6*, and *ATF5* cDNAs were obtained by reverse transcription-PCR, human *PROX1* cDNA was purchased from Dnaform, rat *Cebpa* cDNA was kindly provided by A. Iwama, and human *L-MYC* cDNA was obtained from pMXs-Hu-L-Myc (a gift from S. Yamanaka) (Addgene plasmid #26022)^21^. The cDNAs were subcloned into the retroviral vector pGCDNsam (a gift from M. Onodera). To produce recombinant retroviruses, plasmid DNA was transfected into Plat-GP cells using linear polyethylenimine (PEI) (Polysciences). At 3 days before transfection, Plat-GP cells (1.8 × 10^6^) were plated on poly-L-lysine-coated 10-cm dishes and cultured in DMEM containing 8% FBS, 2 mM L-glutamine, and penicillin/streptomycin. Meanwhile, 36 µL of 1 mg mL^–1^ PEI, 10 µg of retroviral plasmid DNA, and 2 µg of the VSV-G expression plasmid pCMV-VSV-G (a gift from H. Miyoshi) were diluted in 1 mL of DMEM and incubated for 15 min at room temperature. The mixture was then added to the plated Plat-GP cells in a drop-by-drop manner. After 6 h of incubation at 37 °C under 5% CO_2_, the medium was replaced with fresh medium, and the culture was continued. Supernatants from the transfected cells were collected 24 h after medium replacement, filtered through 0.2-µm cellulose acetate filters (Sartorius), and concentrated by centrifugation (10,000 × *g* for 16 h at 4 °C). The viral pellets were resuspended in Hanks’ balanced salt solution (Nissui) (1/140 of initial supernatant volume). HUVECs and HPBECs were grown on gelatin-coated 12-well plates and type I collagen-coated 48-well plates, respectively, until they reached 5–10% and 20–30% confluency, respectively. Then HUVECs and HPBECs were incubated in HUVEC medium and HPBEC medium, respectively, containing the concentrated viral supernatants and 5 µg mL^–1^ protamine sulfate (Nacalai Tesque) for 6 h. The viral infection was serially repeated four or eight times for HUVECs or HPBECs, respectively. A step-by-step protocol describing the generation of hiHepPCs and the induction of hiHepPC differentiation can be found at Protocol Exchange^30^.

### Gene expression analysis

qPCR analyses using TaqMan probes (Applied Biosystems) were conducted as described previously^4^. Briefly, we prepared total RNA using an ISOGEN II (Nippongene) and synthesized cDNA from total RNA using a SuperScript III Reverse Transcriptase (Thermo Fisher Scientific) according to the manufacturer’s instructions. After various dilutions of template cDNA, we optimized their concentration for each probe. In these concentrations, amplification by PCR did not reach plateau but could be used for quantitative analysis. The TaqMan probes for *CYP1A2* (Hs00167927_m1), *CYP2B6* (Hs03044634_m1), *CYP2C9* (Hs00426397_m1), *CYP2C19* (Hs00426380_m1), *CYP2E1* (Hs00559368_m1), *CYP3A4* (Hs00430021_m1), *UGT1A1* (Hs02511055_s1), *UGT1A6* (Hs01592477_m1), *UGT2B4* (Hs02383831_s1), *UGT2B15* (Hs00870076_s1), *ABCB1* (Hs00184500_m1), *ABCC2* (Hs00166123_m1), *ABCG2* (Hs01053790_m1), and *ASGPR1* (Hs01005019_m1) were used. TaqMan Gene Expression Assay IDs (Applied Biosystems) are shown in parentheses following the gene names. As a normalization control, Human GAPDH Endogenous Control (Applied Biosystems) was used. To examine the expression of other genes, qPCR was conducted using a THUNDERBIRD SYBR qPCR Mix (Toyobo) according to the manufacturer’s instructions. qPCR primers for *AFP*, *DLK1*, *SOX17*, *CXCR4*, *TERT*, *CK19*, *HNF1B*, *SOX9*, *CFTR*, *SCTR*, *EpCAM*, *ALB*, *AAT*, *HNF4A*, *CDX1*, *CDX2*, *olfactomedin 4* (*OLFM4*), *sucrase isomaltase* (*SI*), *trefoil factor 3* (*TFF3*), exogenous *FOXA3*, exogenous *HNF1A*, exogenous *HNF6*, and *GAPDH* are listed in Supplementary Table 1. The values for *GAPDH* were used as normalization controls. Adult human hepatocytes obtained immediately after the thawing of cryopreserved hepatocytes (Lonza) were used as controls for qPCR analyses. A recent study demonstrated that the gene expression pattern of cryopreserved hepatocytes is not very different from that of freshly isolated hepatocytes^31^.

### Immunostaining

Immunofluorescence staining for HUVECs, HPBECs, and hiHepPCs in monolayer cultures was conducted as described previously^4^. Paraffin sections of liver tissues, hiHepPC aggregates, and hiHepPC spheroids were prepared and analyzed by immunofluorescence and immunohistochemical staining as described previously^4,10^. Briefly, cultured cells were washed with PBS and sequentially fixed with 2% paraformaldehyde for 5 min at room temperature. The fixed cells were washed in PBS and treated with 0.2% Triton X-100 (Nacalai Tesque) for 1 h at room temperature. Liver tissues and hiHepPC aggregates and spheroids were fixed in a Zinc Formalin Fixative (Polysciences), dehydrated in ethanol and xylene, embedded in paraffin wax, and sectioned. After deparaffinization and rehydration of the sections, antigen retrieval was performed by microwaving in 0.01 M citrate buffer (pH 6.0). For immunohistochemistry, the sections were then incubated with 0.3% hydrogen peroxide in methanol for 20 min at room temperature to quench endogenous peroxidase activity. After washing with PBS and blocking with a Block Ace (DS Pharma Biomedical), the cultured cells and tissue sections were incubated with primary antibodies. Then the cultured cells and tissue sections were washed and incubated with secondary antibodies specific to the species of the primary antibodies with 4,6-diamidino-2-phenylindole for immunofluorescence staining. Primary and secondary antibodies used in this study are listed in Supplementary Table 2. Cellular F-actin was stained using Alexa 488-conjugated phalloidin (Thermo Fisher Scientific). For flow cytometric analyses of immunostained cells, hiHepPC aggregates were trypsinized, fixed using a Cytofix/Cytoperm Kit (BD Biosciences), and stained with appropriate primary and secondary antibodies. The immunostained cells were analyzed using the FACS Jazz and BD FACS Sortware sorter software. Gating strategy is shown in Supplementary Fig. 19.

### Analysis of hepatic function

Cell aggregates formed from HUVEC-derived and HPBEC-derived hiHepPCs were cultured for 14 and 7 days, respectively, after initiation of 3D culture. Then these aggregates were trypsinized, and 1 × 10^5^ cells were plated on each well of type I collagen-coated 96-well plates. Similarly, 1 × 10^5^ HUVECs, 1 × 10^5^ HPBECs, 1 × 10^5^ cells obtained from hiHepPC monolayer cultures, and 1 × 10^5^ human hepatocytes were plated on each well of type I collagen-coated 96-well plates. The amounts of urea or human ALB in the culture media were measured after culture of cells with or without 5 mM ammonium chloride (Nacalai Tesque), respectively, for 24 h. ALB and urea were detected using an Albuwell II (Exocell) and a QuantiChrom Urea Assay Kit (BioAssay Systems), respectively, according to the manufacturers’ instructions. The absorbance signals were measured with a Multiskan FC microplate reader (Thermo Fisher Scientific). CYP3A4 and CYP2C9 activities were measured after culture of cells with or without 25 µM rifampicin (Nacalai Tesque) for 48 h using a P450-Glo CYP3A4 Assay Kit (with Luciferin-IPA) and P450-Glo CYP2C9 Assay Kit (both from Promega), respectively, according to the manufacturer’s instructions. The luminescent signals were measured with a Luminescencer Octa (ATTO). Cell viability was measured with Cell Count Reagent SF (Nacalai Tesque) after treatment of 1 × 10^4^ cells plated on each well of type I collagen-coated 96-well plates with amiodarone (Sigma-Aldrich), diclofenac (Sigma-Aldrich), or acetaminophen (Sigma-Aldrich) for 24 h. The absorbance signals were measured with a Multiskan FC microplate reader. ICG uptake was evaluated after culture of HUVECs, HPBECs, and hiHepPCs with 1 mg mL^–1^ cardiogreen (Sigma-Aldrich) for 30 min, and ICG release was subsequently analyzed at 6 h after excluding cardiogreen from the medium. Oil red O staining was conducted as described previously^32^. Briefly, cultured cells were fixed in 10% formalin for 10 min at room temperature, washed with PBS, and treated with 60% isopropanol for 1 min at room temperature. The cells were then incubated with 60% Oil red O (Muto Pure Chemicals) in water for 20 min at room temperature. After washing with 60% isopropanol for 1 min and additional washing with PBS, the cells were incubated with hematoxylin (Muto Pure Chemicals) for 5 min. A functional feature of bile canaliculi was visualized by incubating hiHepPC aggregates with 5 μM carboxy-2′,7′-dichlorofluorescein diacetate (Molecular Probes) for 20 min at 37 °C under 5% CO_2_, followed by washing with PBS (Nacalai Tesque).

### Analysis of cholangiocyte function

MDR1 transporter activity in hiHepPC spheroids was analyzed as described previously^33^. Briefly, 3D cultures of hiHepPC spheroids were incubated with 100 µM rhodamine 123 (Sigma-Aldrich) for 10 min at 37 °C under 5% CO_2_. For inhibition of MDR1 transporter activity, rhodamine 123 was added to the culture medium after 30 min of incubation with 20 µM R(+)-verapamil (Sigma-Aldrich). The function of CFTR in the hiHepPC spheroids was evaluated as described previously^33^. Briefly, hiHepPC spheroids were cultured in human cholangiocyte culture medium without FSK for 24 h, and then 10 µM FSK or FSK plus 100 µM CFTRinh-172 (Sigma-Aldrich) were added to the medium. The diameter of 20 spheroids was measured before addition of FSK or FSK plus CFTRinh-172 to the medium, and the diameter of corresponding spheroids was also measured after culture with or without FSK and/or CFTRinh-172 for 24 h.

### Transcriptome analysis

Total RNA was obtained from the primary monolayer cultures of HUVECs and HPBECs, which were derived from two different donors, respectively; three different HUVEC-derived hiHepPCs at passage 8 and two different HPBEC-derived hiHepPCs at passage 12 in monolayer culture; cell aggregates and cystic spheroids formed from these HUVEC-derived hiHepPCs at day 21 and passage 9, respectively, and those from HPBEC-derived hiHepPCs at day 7 and passage 6, respectively, after initiation of 3D culture; three different HUVEC-derived hiHepPCs at passage 16 in monolayer culture; human fetal cholangiocyte-derived spheroids at passage 2 in 3D culture; and adult human hepatocytes obtained immediately after the thawing of cryopreserved hepatocytes (Lonza) that were derived from two different donors using a NucleoSpin RNA II kit (Macherey-Nagel) according to the manufacturers’ instructions and analyzed with CEL-seq2^34^. CEL-seq2 was originally developed as a single-cell RNA-seq method^34^, but it is also available as an RNA-seq method for cell populations^35^. Library preparation and sequencing were conducted as follows: (1) barcoding the samples with a set of primers including an unique molecular identifier (UMI), (2) converting mRNA to double-stranded DNA using a SuperScript II Double-Stranded cDNA Synthesis Kit (Thermo Fisher Scientific), (3) nucleic acid purification with AMPure XP beads (Beckman Coulter), and (4) converting antisense RNA to cDNA using random priming with a random hexamer containing an Illumina 3′ adaptor sequence^34,35^. All sequencing experiments were performed on an Illumina HiSeq 1500 system. Reads were mapped to the human reference genome (Ensemble GRCh38) using Bowtie2 (v2.3.0) and converted into UMI counts of each gene. Differentially expressed genes (DEGs) were extracted using the TCC (v1.12.0) (with an adjusted *q*-value of <0.05)^36^, DESeq2 (v1.26.0) (with an adjusted *q*-value of <0.1)^37^, or edgeR (v3.28.1) (with an adjusted *q*-value of <0.1)^38,39^ packages for R (v3.6.0). Functional enrichment analyses of the DEGs were performed using the Database for Annotation, Visualization, and Integrated Discovery (DAVID) (v6.8)^40,41^. GSEA for cross-platform comparison of the gene expression was performed using “data sets” and “gene sets” calculated from the present CEL-seq2 data sets or previously reported microarray and RNA-seq data sets as described previously^42^. Briefly, for preparation of the “data sets”, transcriptome-wide ranking lists of the gene expression fold change between sample pairs were calculated with GEO2R program in the Gene Expression Omnibus (GEO) for publicized microarray data (Accession Numbers: GEO: GSE42643, GSE63859, and GSE98324)^6,13,14^ and edgeR package^38,39^ on R software for the present CEL-seq2 data (Accession Number: GEO: GSE120732) and publicized RNA-seq data (Accession Number: GEO: GSE54066)^5^. Gene symbols and fold change score were sorted in descending order, and genes with no expression change between the paired samples were eliminated using the Microsoft Excel software (v16.16). For preparation of the “gene sets”, gene symbol lists of top 100, 200, and 500 genes for each “data set” were created for the following data pairs: Human ESC-derived Hepatic Progenitor Under Base Condition vs Undifferentiated Human ESCs (GSE98324); EM vs Tissue (GSE63859); iHep vs HEF (GSE54066); hiHep vs HFF (GSE42643); hiHepLT vs HFF (GSE42643); human-hepatocyte vs HUVEC or HPBEC (GSE120732); human-cholangiocyte-spheroid-culture vs HUVEC or HPBEC (GSE120732); and HUVEC-hiHepPC-aggregate vs HUVEC (GSE120732). Both “data sets” and “gene sets” data were processed with the GSEAPreranked program (v5) in GSEA software (v3.0) with “classic” enrichment statistic parameter^43,44^. PCA based on the whole-transcriptome data was performed using CEL-seq2 data sets after upper quantile normalization on R software with or without the rgl (v0.99.16) package for 3D or two-dimensional plots, respectively.

### Transplantation

Before transplantation of hepatocytes, the recipient NSG male mice (3 weeks old) were administered 40 mg kg^–1^ retrorsine (Sigma-Aldrich) by intraperitoneal injection and subsequently given 80 mg kg^–1^ retrorsine two times every other week. One week after the last injection of retrorsine, the recipient mice (6 weeks old) received 70% PH, followed by intrasplenic injection of PBS, 1 × 10^6^ HUVECs, 1 × 10^6^ cells obtained from hiHepPC monolayer cultures, 1 × 10^6^ cells dissociated from hiHepPC aggregates, and 1 × 10^6^ human hepatocytes obtained immediately after the thawing of cryopreserved hepatocytes into the liver. Cell aggregates formed from HUVEC-derived and HPBEC-derived hiHepPCs were cultured for 14 and 7 days, respectively, after initiation of 3D culture. Then these aggregates were trypsinized, and dissociated 1 × 10^6^ cells were used as donor cells for transplantation. Liver tissues and serum were obtained from the recipient mice 2 months after transplantation, and the amounts of human ALB in the mouse serum were measured using a Human ALB ELISA Kit (Bethyl) according to the manufacturer’s instructions. The amounts of AST and ALT in the mouse serum obtained at day 2 after transplantation were measured using a Transaminase CII-test Kit (Wako) according to the manufacturer’s instructions. Hepatocyte proliferation in the liver was evaluated by 5-bromo-2’-deoxyuridine (BrdU) incorporation into DNA for 1 h after intraperitoneal injection of 50 mg kg^–1^ BrdU (Nacalai Tesque) into mice. For cholangiocyte transplantation, the recipient NSG mice (10 weeks old) were fed a diet containing 0.1% DDC (Sigma-Aldrich) to induce chronic liver injury and primitive ductule formation for 10 weeks until the analysis of liver tissues. At 4 and 6 weeks after initiation of DDC treatment, 2 × 10^6^ cells dissociated from HUVEC-derived and HPBEC-derived hiHepPC spheroids at passage 9 and 6, respectively, and human fetal cholangiocyte-derived spheroids at passage 3 in 3D culture with Matrigel were intrasplenically injected into the DDC-treated recipient mouse livers. We prepared HUVEC-derived and HPBEC-derived hiHepPCs and cell aggregates and spheroids formed from these hiHepPCs in at least three independent experiments and transplanted them into more than three recipient mice. The hiHepPCs and their progenies prepared in all experiments were able to become engrafted and reconstitute liver tissues after transplantation.

### Tumorigenicity assay

In all, 5 × 10^6^ HuH7, 5 × 10^6^ HPBEC-derived MYC-hiHepPCs, 5 × 10^6^ HPBEC-derived hiHepPCs, and 5 × 10^6^ HUVEC-derived hiHepPCs were obtained from monolayer cultures, resuspended in 100 μL of Matrigel in PBS (1:1), and subcutaneously injected into the recipient NOD/SCID female mice (10 weeks old). HuH7 or hiHepPCs were allowed to grow for 1 or 2 months after injection, respectively, and the weights of tumors formed from these cells were measured.

### Karyotype assay

Karyotypes of HUVEC-derived hiHepPCs in monolayer culture were determined at the Chromocenter in Japan. Karyotype analyses for cells composing HUVEC-derived hiHepPC spheroids in 3D culture with Matrigel and HPBEC-derived MYC-hiHepPCs and hiHepPCs in monolayer culture were conducted as described previously^10^. Briefly, cells treated with 0.05 μg mL^–1^ colcemid solution (Nacalai Tesque) were processed using a standard karyotyping protocol.

### CGH analysis

hiHepPCs were generated from four different HUVECs derived from two male donors (Takara, lots: 425Z002 and 425Z007) and two female donors (Gibco, lot: 1814635 and Lonza, lot: 00022). Copy number variations between these hiHepPCs at passage 12 in monolayer culture and each parental HUVEC were determined by Takara in Japan using a SurePrint G3 Human CGH Microarray Kit (Agilent). The data were analyzed using an Agilent Genomic Workbench software (v7.0) and deposited in the GEO database (Accession Number: GEO: GSE118910).

### Cellular senescence assay

hiHepPCs in monolayer cultures were fixed with PBS containing 2% formaldehyde (Nacalai Tesque) and 0.2% glutaraldehyde (Nacalai Tesque), and senescence-associated β-galactosidase activities in hiHepPCs were analyzed as described previously^45^. Briefly, the fixed cells were washed in PBS and incubated for 16 h at 37 °C with a mixture of 0.5 mg mL^–1^ X-gal (Anatrace) and 40 mM citric acid/sodium phosphate buffer (pH 6.0) containing 5 mM potassium ferricyanide (Wako), 5 mM potassium ferrocyanide (Wako), and 2 mM magnesium chloride (Wako).

### Cell cycle analysis

hiHepPCs in monolayer cultures were trypsinized, fixed with cold 70% ethanol for 16 h at −30 °C, treated with 20 μg mL^–1^ RNase A (Thermo Fisher Scientific) for 30 min at 37 °C, and stained with 100 μg mL^–1^ propidium iodide (PI) for 10 min at room temperature. The PI-stained cells were analyzed using the FACS Jazz and BD FACS Sortware sorter software. Gating strategy is shown in Supplementary Fig. 19.

### Statistics and reproducibility

Statistical significance was analyzed using two-sided Student’s *t* test and one-way analysis of variance followed by Tukey–Kramer test or Dunnett’s test. Kaplan–Meier survival curves were statistically analyzed by log-rank test. A difference at *P* < 0.05 was considered statistically significant. The significance of an observed enrichment score in GSEA was assessed by comparing it with the set of null enrichment scores computed with randomly assigned phenotypes. Cell and tissue images presented in the figures and supplementary figures were obtained from at least three independent experiments, and representative images are shown.

### Reporting summary

Further information on research design is available in the Nature Research Reporting Summary linked to this article.

## Supplementary information

Supplementary Information

Reporting Summary

## Data Availability

All data sets were deposited in the GEO database under Accession Numbers GEO: GSE118910 and GEO: GSE120732. The publicly available data sets (Accession Numbers: GEO: GSE42643, GSE63859, GSE98324, and GEO: GSE54066) were also used in this study. All other data supporting the results presented herein are available within the article and Supplementary Information and from the corresponding author upon reasonable request. A reporting summary for this article is available as a Supplementary Information file. Source data are provided with this paper.

## Source data

Source Data

## References

[1] Szkolnicka D, Hay DC (2016). Concise review: advances in generating hepatocytes from pluripotent stem cells for translational medicine. Stem Cells.

[2] Palakkan AA, Nanda J, Ross JA (2017). Pluripotent stem cells to hepatocytes, the journey so far. Biomed. Rep..

[3] Huang P (2011). Induction of functional hepatocyte-like cells from mouse fibroblasts by defined factors. Nature.

[4] Sekiya S, Suzuki A (2011). Direct conversion of mouse fibroblasts to hepatocyte-like cells by defined factors. Nature.

[5] Du Y (2014). Human hepatocytes with drug metabolic function induced from fibroblasts by lineage reprogramming. Cell Stem Cell.

[6] Huang P (2014). Direct reprogramming of human fibroblasts to functional and expandable hepatocytes. Cell Stem Cell.

[7] Ring KL (2012). Direct reprogramming of mouse and human fibroblasts into multipotent neural stem cells with a single factor. Cell Stem Cell.

[8] Kim YJ (2014). Generation of multipotent induced neural crest by direct reprogramming of human postnatal fibroblasts with a single transcription factor. Cell Stem Cell.

[9] Sandler VM (2014). Reprogramming human endothelial cells to haematopoietic cells requires vascular induction. Nature.

[10] Miura S, Suzuki A (2017). Generation of mouse and human organoid-forming intestinal progenitor cells by direct lineage reprogramming. Cell Stem Cell.

[11] Yu B (2013). Reprogramming fibroblasts into bipotential hepatic stem cells by defined factors. Cell Stem Cell.

[12] Lim KT (2018). Direct conversion of mouse fibroblasts into cholangiocyte progenitor cells. Stem Cell Rep..

[13] Ang LT (2018). A roadmap for human liver differentiation from pluripotent stem cells. Cell Rep..

[14] Huch M (2015). Long-term culture of genome-stable bipotent stem cells from adult human liver. Cell.

[15] Ayabe H (2018). Optimal hypoxia regulates human iPSC-derived liver bud differentiation through intercellular TGFB signaling. Stem Cell Rep..

[16] Ogawa S (2013). Three-dimensional culture and cAMP signaling promote the maturation of human pluripotent stem cell-derived hepatocytes. Development.

[17] Yamamoto J, Udono M, Miura S, Sekiya S, Suzuki A (2018). Cell aggregation culture induces functional differentiation of induced hepatocyte-like cells through activation of Hippo signaling. Cell Rep..

[18] Laconi E (1998). Long-term, near-total liver replacement by transplantation of isolated hepatocytes in rats treated with retrorsine. Am. J. Pathol..

[19] Basma H (2009). Differentiation and transplantation of human embryonic stem cell-derived hepatocytes. Gastroenterology.

[20] Tanimizu N (2016). Liver progenitors isolated from adult healthy mouse liver efficiently differentiate to functional hepatocytes in vitro and repopulate liver tissue. Stem Cells.

[21] Nakagawa M, Takizawa N, Narita M, Ichisaka T, Yamanaka S (2010). Promotion of direct reprogramming by transformation-deficient Myc. Proc. Natl Acad. Sci. USA.

[22] Cirillo LA (2002). Opening of compacted chromatin by early developmental transcription factors HNF3 (FoxA) and GATA-4. Mol. Cell.

[23] Zaret KS, Carroll JS (2011). Pioneer transcription factors: establishing competence for gene expression. Genes Dev..

[24] Horisawa, K. et al. The dynamics of transcriptional activation by hepatic reprogramming factors. *Mol. Cell* **79**, 660–676 (2020). 10.1016/j.molcel.2020.07.012 32755593

[25] Costa RH, Kalinichenko VV, Holterman AX, Wang X (2003). Transcription factors in liver development, differentiation, and regeneration. Hepatology.

[26] Clotman F (2002). The onecut transcription factor HNF6 is required for normal development of the biliary tract. Development.

[27] Raven A (2017). Cholangiocytes act as facultative liver stem cells during impaired hepatocyte regeneration. Nature.

[28] Geti I (2012). A practical and efficient cellular substrate for the generation of induced pluripotent stem cells from adults: blood-derived endothelial progenitor cells. Stem Cells Transl. Med..

[29] Chang WY (2013). Feeder-independent derivation of induced-pluripotent stem cells from peripheral blood endothelial progenitor cells. Stem Cell Res..

[30] Inada, H., Udono, M. & Suzuki, A. Direct conversion of human endothelial cells to hepatic progenitor cells. *Protoc. Exch*. (in the press).

[31] Aizarani N (2019). A human liver cell atlas reveals heterogeneity and epithelial progenitors. Nature.

[32] Miura S, Suzuki A (2014). Acquisition of lipid metabolic capability in hepatocyte-like cells directly induced from mouse fibroblasts. Front. Cell Dev. Biol..

[33] Ogawa M (2015). Directed differentiation of cholangiocytes from human pluripotent stem cells. Nat. Biotechnol..

[34] Hashimshony T (2016). CEL-Seq2: sensitive highly-multiplexed single-cell RNA-Seq. Genome Biol..

[35] Hara M (2017). Interaction of reactive astrocytes with type I collagen induces astrocytic scar formation through the integrin-N-cadherin pathway after spinal cord injury. Nat. Med..

[36] Sun J, Nishiyama T, Shimizu K, Kadota K (2013). TCC: an R package for comparing tag count data with robust normalization strategies. BMC Bioinformatics.

[37] Love MI, Huber W, Anders S (2014). Moderated estimation of fold change and dispersion for RNA-seq data with DESeq2. Genome Biol..

[38] Robinson MD, McCarthy DJ, Smyth GK (2010). edgeR: a Bioconductor package for differential expression analysis of digital gene expression data. Bioinformatics.

[39] McCarthy DJ, Chen Y, Smyth GK (2012). Differential expression analysis of multifactor RNA-Seq experiments with respect to biological variation. Nucleic Acids Res..

[40] Huang W, Sherman BT, Lempicki RA (2009). Bioinformatics enrichment tools: Paths toward the comprehensive functional analysis of large gene lists. Nucleic Acids Res..

[41] Huang W, Sherman BT, Lempicki RA (2009). Systematic and integrative analysis of large gene lists using DAVID bioinformatics resources. Nat. Protoc..

[42] Takashima Y, Horisawa K, Udono M, Ohkawa Y, Suzuki A (2018). Prolonged inhibition of hepatocellular carcinoma cell proliferation by combinatorial expression of defined transcription factors. Cancer Sci..

[43] Mootha VK (2003). PGC-1α-responsive genes involved in oxidative phosphorylation are coordinately downregulated in human diabetes. Nat. Genet..

[44] Subramanian A (2005). Gene set enrichment analysis: a knowledge-based approach for interpreting genome-wide expression profiles. Proc. Natl Acad. Sci. USA.

[45] Debacq-Chainiaux F, Erusalimsky JD, Campisi J, Toussaint O (2009). Protocols to detect senescence-associated beta-galactosidase (SA-βgal) activity, a biomarker of senescent cells in culture and *in vivo*. Nat. Protoc..

