# Supplementary material for: Direct reprogramming of human umbilical vein- and peripheral blood-derived endothelial cells into hepatic progenitor cells
Source: Nat Commun. 2020 Oct 21;11:5292. doi: 10.1038/s41467-020-19041-z (PMC7578104; doi:10.1038/s41467-020-19041-z)
Supplement: Supplementary file 1 — Supplementary Information [file 41467_2020_19041_MOESM1_ESM.pdf]

SUPPLEMENTARY INFORMATION

Direct reprogramming of human umbilical vein- and peripheral blood-derived endothelial cells into hepatic progenitor cells

Inada et al.

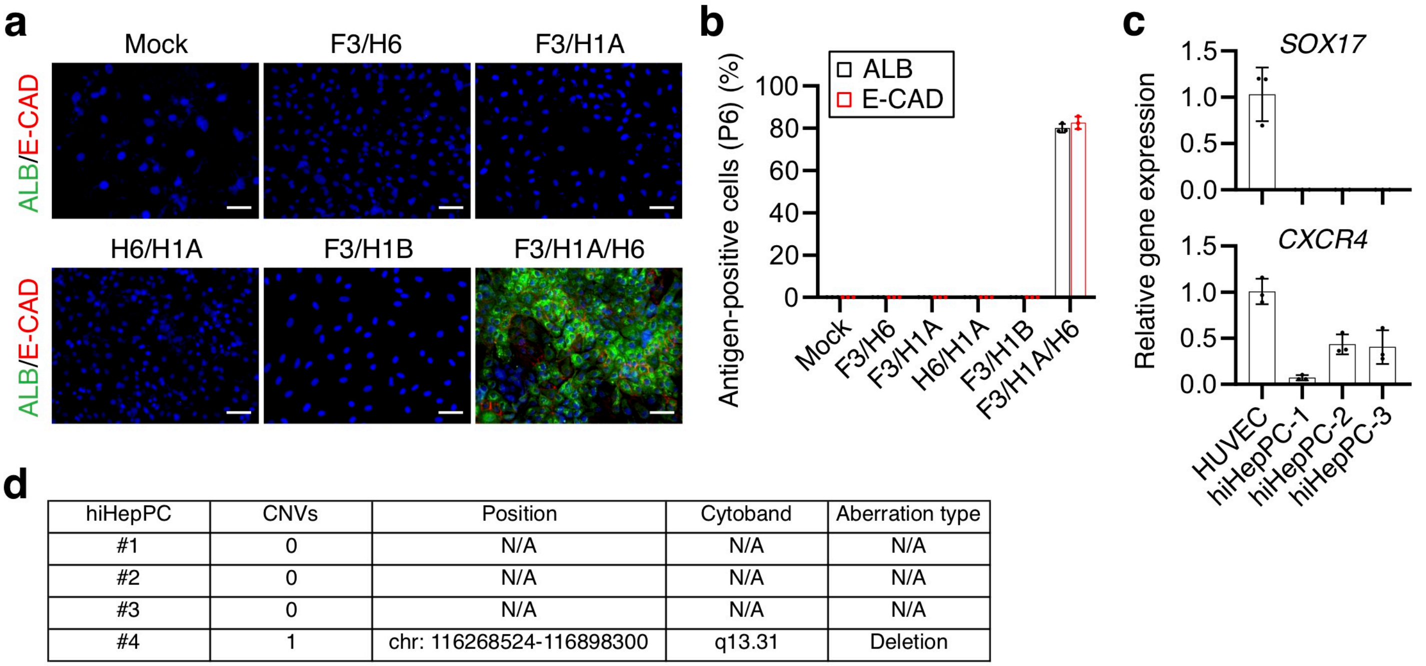

d

hiHepPC	CNVs	Position	Cytoband	Aberration type
#1	0	N/A	N/A	N/A
#2	0	N/A	N/A	N/A
#3	0	N/A	N/A	N/A
#4	1	chr: 116268524-116898300	q13.31	Deletion

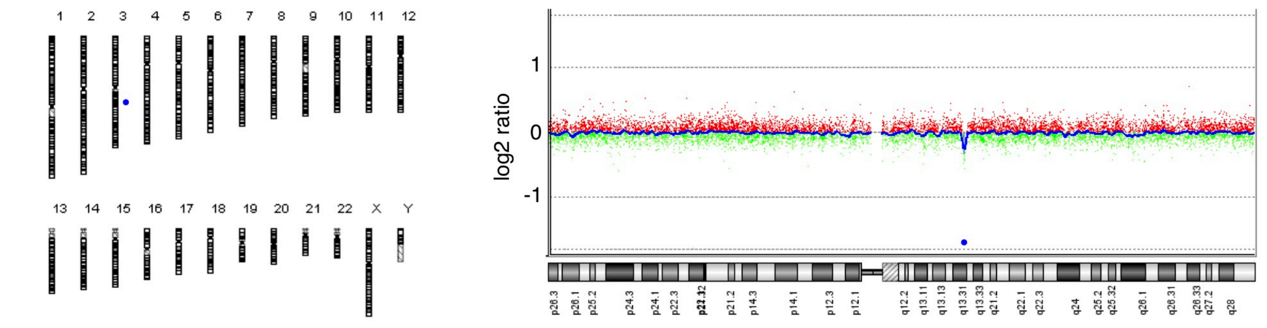

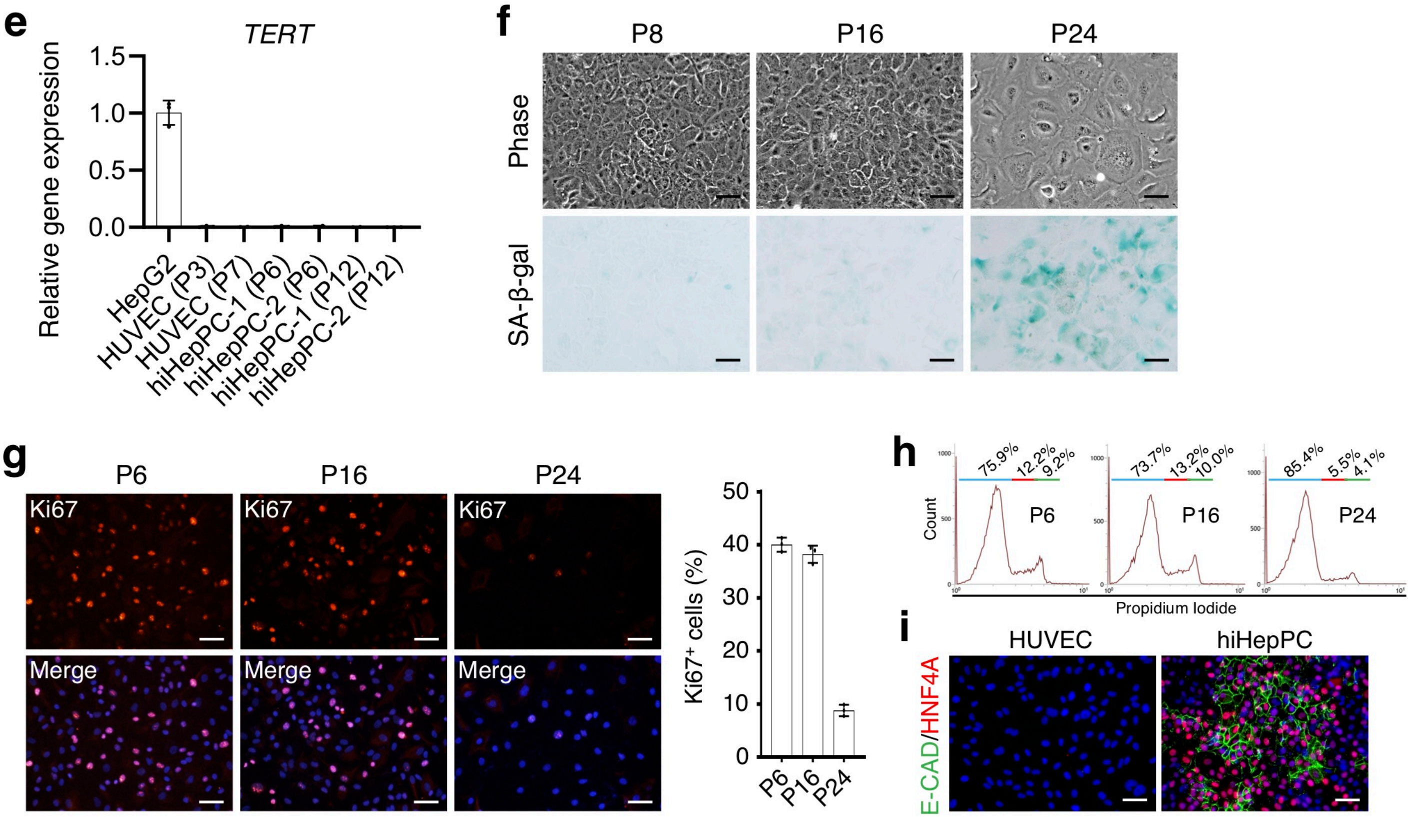

Supplementary Figure 1. Additional analyses of hepatic reprogramming factors and further characterization of hiHepPCs. (**a**) Co-immunofluorescence staining of ALB with E-CAD was conducted for mock-infected HUVECs and HUVECs transduced with the indicated factors at passage 6. FOXA3, HNF1A, HNF6, and HNF1B are abbreviated as F3, H1A, H6, and H1B, respectively. (**b**) The percentages of cells immunoreactive for ALB or E-CAD among mock-infected HUVECs and HUVECs transduced with the indicated factors at passage (P) 6. Data represent the mean ± SD (*n* = 3 independent experiments). (**c**) qPCR analyses of *SOX17* and *CXCR4* were performed on total RNA obtained from HUVECs at passage (P) 2 and three different hiHepPCs at P8 in monolayer culture. All data were normalized with the values for HUVECs, and the fold differences are shown. Data represent the mean ± SD (*n* = 3 independent assays). (**d**) CGH analyses were performed on genomic DNA extracted from four different hiHepPCs at passage 12 in monolayer culture and each parental HUVEC. As shown by blue dots, only a deletion was found on chromosome 3 in one of the four hiHepPCs compared with the parental HUVECs. CNV, copy number variation. (**e**) qPCR analyses of *TERT* were performed on total RNA obtained from the human hepatocellular carcinoma cell line HepG2, HUVECs at passage (P) 3 and P7, and two different hiHepPCs at P6 and P12 in monolayer culture. All data were normalized with the values for HepG2, and the fold differences are shown. Data represent the mean ± SD (*n* = 3 independent assays). (**f**) Representative morphologies of hiHepPCs and representative SA-β-gal activities in hiHepPCs at passage (P) 8, P16, and P24 in monolayer culture. (**g**) Immunofluorescence staining of Ki67 was conducted for hiHepPCs at passage (P) 6, P16, and P24 in monolayer culture. The graph shows the percentages of $Ki67^+$ cells in hiHepPC monolayer cultures at the indicated passage numbers. Data represent the mean ± SD (*n* = 3 independent experiments). (**h**) Cell cycle analyses of hiHepPCs at passage (P) 6, P16, and P24 in monolayer culture were performed using flow cytometry. Blue, red, and green lines show cells in G0/G1, S, and G2/M phases, respectively. The experiments were repeated three times, and representative histograms are shown. (**i**) Co-immunofluorescence staining of E-CAD with HNF4A was conducted for mock-infected HUVECs and HUVEC-derived hiHepPCs. DNA was stained with DAPI. Scale bars, 50 μm. Source data are provided as a Source Data file.

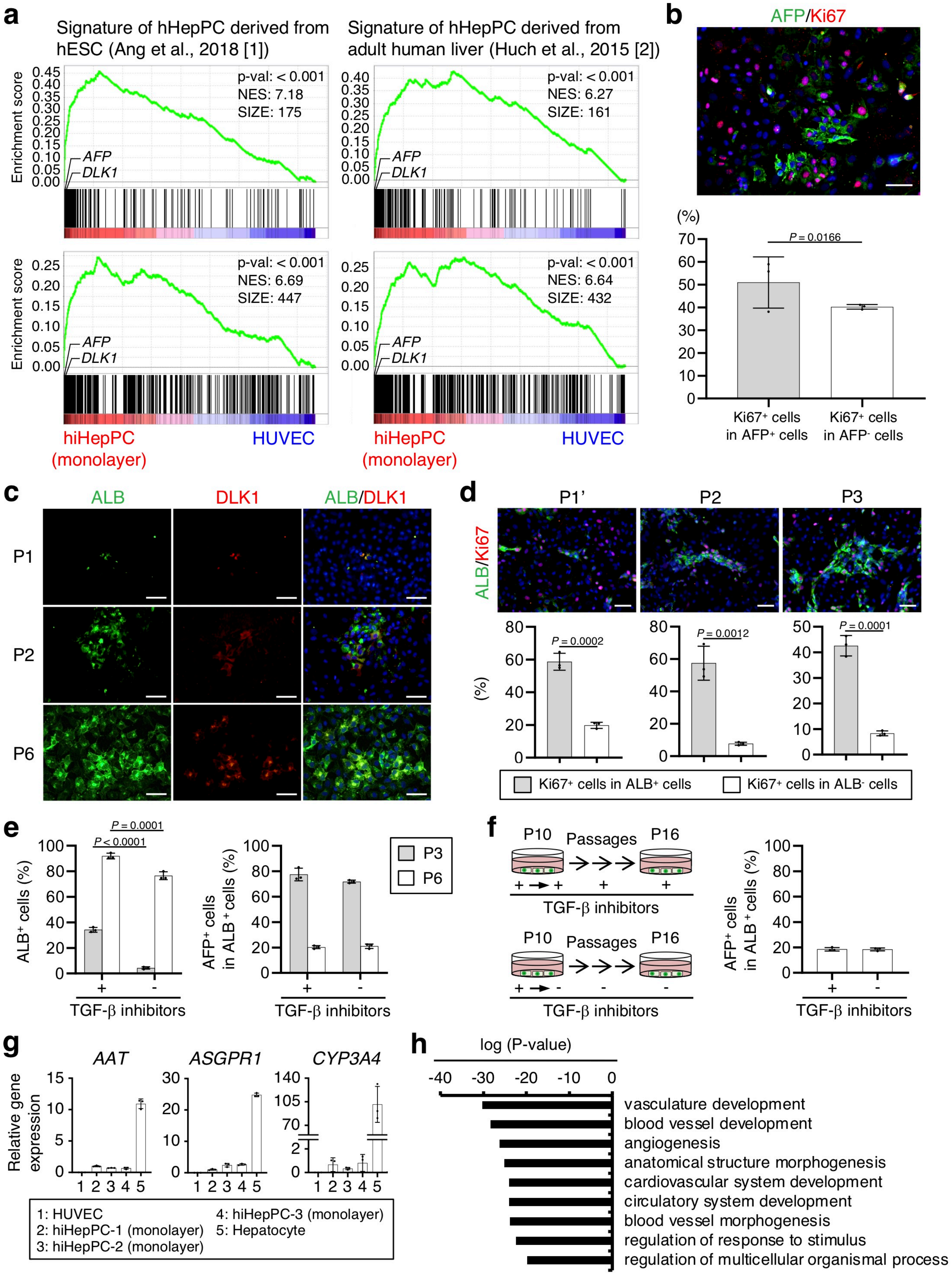
a
Signature of hHepPC derived from hESC (Ang et al., 2018 [1])
Signature of hHepPC derived from adult human liver (Huch et al., 2015 [2])
Enrichment score
p-val: < 0.001
NES: 7.18
SIZE: 175
NES: 6.27
SIZE: 161
NES: 6.69
SIZE: 447
NES: 6.64
SIZE: 432
AFP
DLK1
hiHepPC (monolayer)
HUVEC
b
AFP/Ki67
P = 0.0166
Ki67+ cells in AFP+ cells
Ki67+ cells in AFP- cells
c
ALB
DLK1
ALB/DLK1
P1
P2
P6
d
P1'
P2
P3
ALB/Ki67
P = 0.0002
P = 0.0012
P = 0.0001
Ki67+ cells in ALB+ cells
Ki67+ cells in ALB- cells
e
P = 0.0001
P < 0.0001
ALB+ cells (%)
AFP+ cells in ALB+ cells (%)
P3
P6
TGF-β inhibitors
f
P10
Passages
P16
TGF-β inhibitors
AFP+ cells in ALB+ cells (%)
g
AAT
ASGPR1
CYP3A4
Relative gene expression
1: HUVEC
2: hiHepPC-1 (monolayer)
3: hiHepPC-2 (monolayer)
4: hiHepPC-3 (monolayer)
5: Hepatocyte
h
log (P-value)
vasculature development
blood vessel development
angiogenesis
anatomical structure morphogenesis
cardiovascular system development
circulatory system development
blood vessel morphogenesis
regulation of response to stimulus
regulation of multicellular organismal process

Supplementary Figure 2. Immature phenotype and proliferative activity of hiHepPCs. (**a**) GSEA of CEL-seq2 data for hiHepPC monolayer cultures and HUVECs was performed using the set of top 200 (upper panels) and 500 (lower panels) genes specifically upregulated in hHepPCs derived from hESCs[1] or adult human livers[2]. (**b**) Co-immunofluorescence staining of AFP with Ki67 was conducted for hiHepPCs at passage 6 in monolayer culture. The graph shows the percentages of $Ki67^+$ cells in AFP^+ or AFP^- cells, which were observed in individual cultures of hiHepPCs. Data represent the mean ± SD (n = 3 independent experiments). (**c**) Co-immunofluorescence staining of ALB with DLK1 was conducted for hiHepPCs induced from HUVECs at the indicated passage numbers (P). P1 designates day 4 after the initial passage of transduced HUVECs. (**d**) Co-immunofluorescence staining of ALB with Ki67 was conducted for hiHepPCs induced from HUVECs at the indicated passage numbers (P). P1' designates day 7 after the initial passage of transduced HUVECs. The graphs show the percentages of $Ki67^+$ cells in ALB^+ or ALB^- cells, which were observed in individual cultures of hiHepPCs. Data represent the mean ± SD (n = 3 independent experiments). (**e**) hiHepPCs were induced from HUVECs in the presence (+) or absence (-) of TGF-β inhibitors (A8301 and SB431542). Co-immunofluorescence staining of ALB with AFP was conducted for hiHepPCs at the indicated passage numbers (P), and the percentages of ALB^+ cells (left graph) and AFP^+ cells in ALB^+ cells (right graph), which were observed in individual cultures of hiHepPCs, are shown. Data represent the mean ± SD (n = 3 independent experiments). (**f**) TGF-β inhibitors (A8301 and SB431542) were withdrawn from the culture medium at passage (P) 10, and hiHepPCs were cultured in the absence (-) of TGF-β inhibitors from P10 to P16. hiHepPCs were also cultured in the presence (+) of TGF-β inhibitors as a control. Co-immunofluorescence staining of ALB with AFP was conducted for hiHepPCs at P16, and the percentage of AFP^+ cells in ALB^+ cells, which were observed in individual cultures of hiHepPCs, is shown. Data represent the mean ± SD (n = 3 independent experiments). (**g**) qPCR analyses of *AAT*, *ASGPR1*, and *CYP3A4* were performed on total RNA obtained from HUVECs at passage (P) 2, three different hiHepPCs at P8 in monolayer culture, and human hepatocytes. All data were normalized with the values for hiHepPC-1 (monolayer), and the fold differences are shown. Data represent the mean ± SD (n = 3 independent assays). (**h**) GOEA was performed for genes with expression levels higher in HUVECs than in hiHepPC monolayer cultures. DNA was stained with DAPI. Scale bar, 50 μm. Statistical difference was determined by two-sided Student's *t*-test (**b**,**d**) or one-way analysis of variance followed by Tukey–Kramer test (**e**). Source data are provided as a Source Data file.

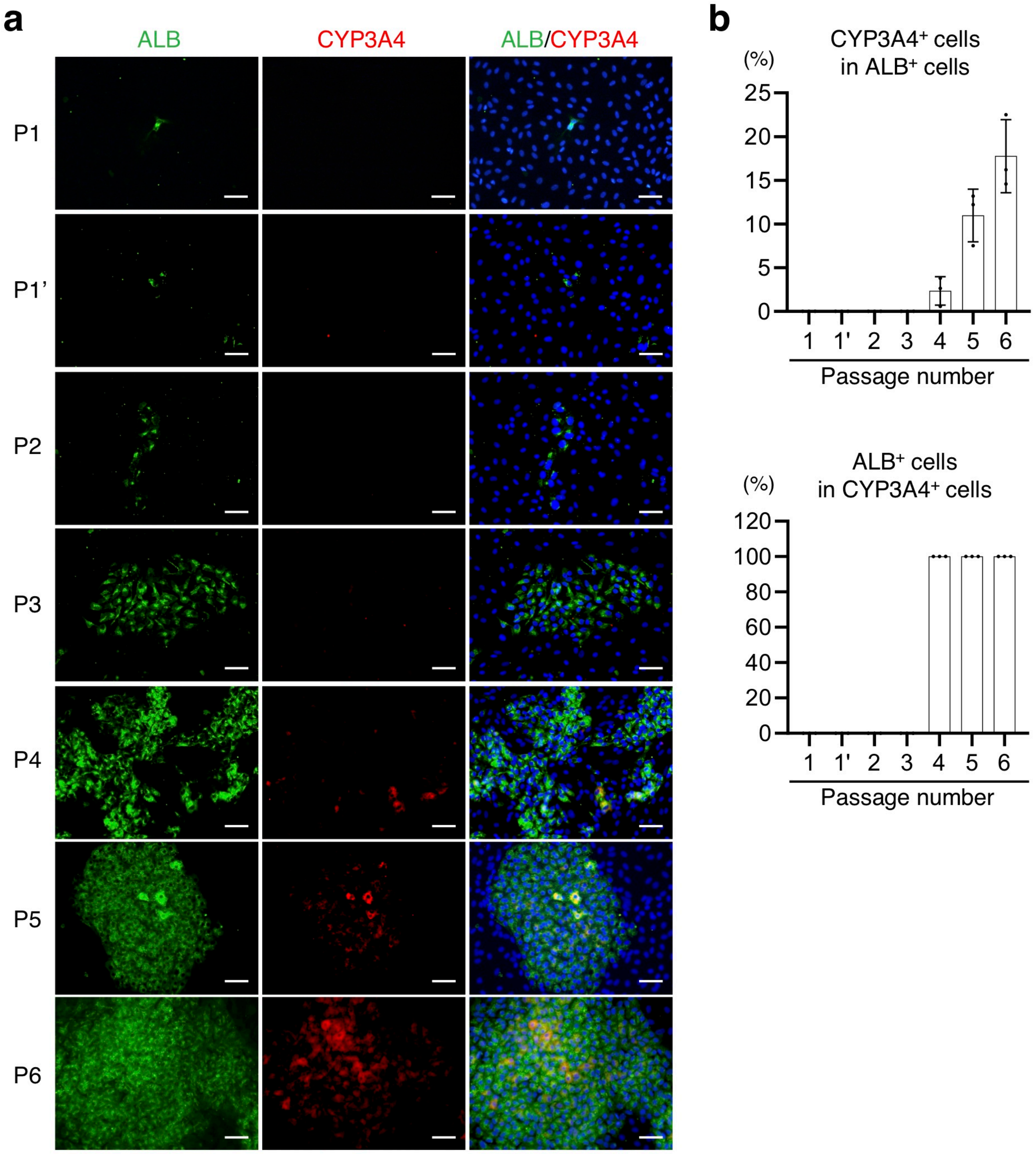

Supplementary Figure 3. ALB$^+$ CYP3A4$^+$ hepatocytes appear in hiHepPC monolayer cultures during passaging. (**a**) Co-immunofluorescence staining of ALB with CYP3A4 was conducted for hiHepPCs induced from HUVECs at the indicated passage numbers (P). P1 and P1' designate day 4 and 7, respectively, after the initial passage of transduced HUVECs. DNA was stained with DAPI. Scale bars, 50 μm. (**b**) The upper and lower graphs show the percentages of CYP3A4$^+$ cells in ALB$^+$ cells and ALB$^+$ cells in CYP3A4$^+$ cells, respectively, which were observed in individual cultures of hiHepPCs during passaging. Data represent the mean ± SD (n = 3 independent experiments). Source data are provided as a Source Data file.

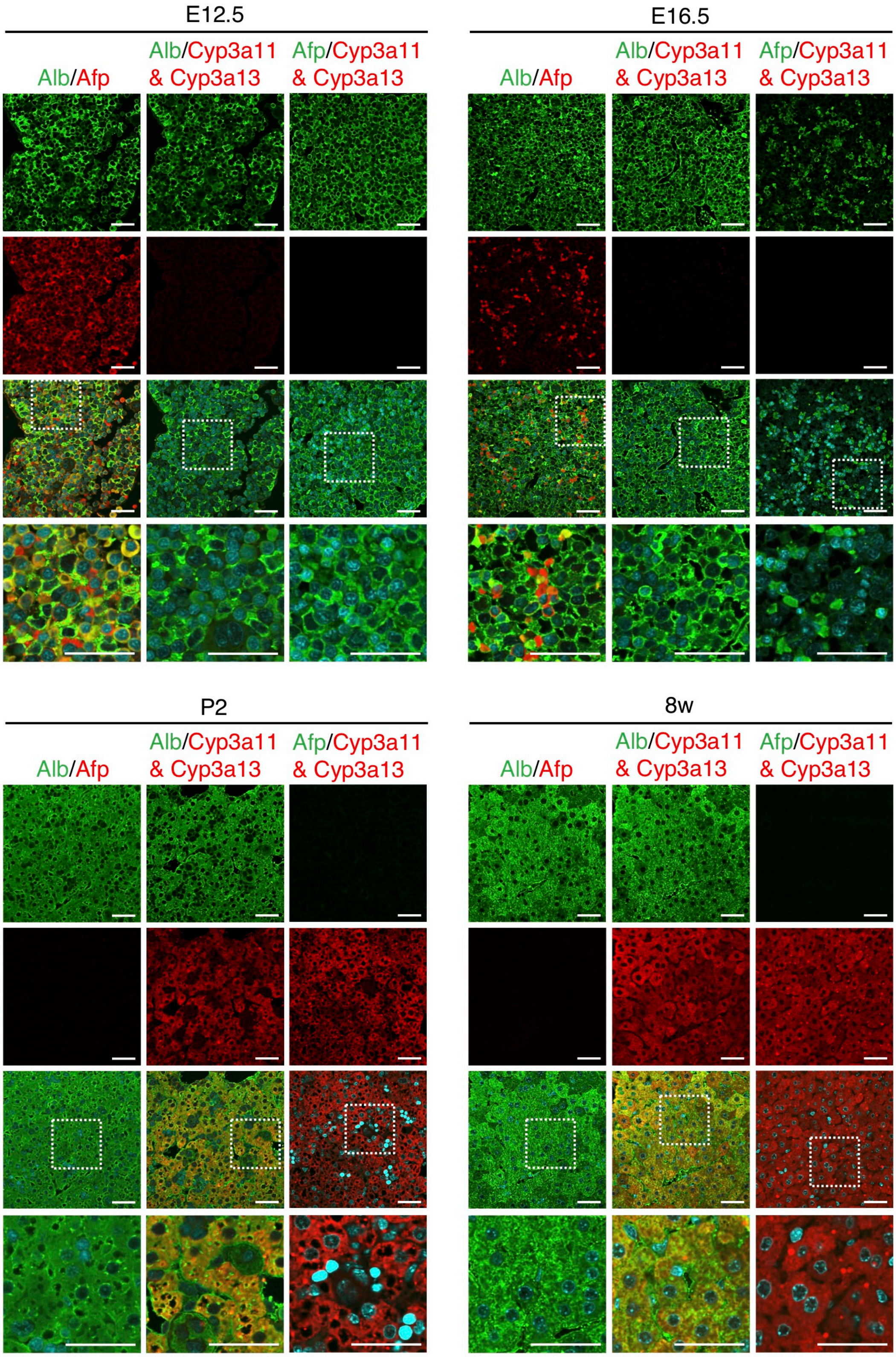

Supplementary Figure 4. Differentiation of mouse hepatoblasts into hepatocytes during liver development. Co-immunofluorescence staining of Alb with Afp or Cyp3a11/Cyp3a13 (mouse homologues to human CYP3A4) and Afp with Cyp3a11/Cyp3a13 were conducted for fetal livers at embryonic day (E) 12.5 and E16.5, postnatal livers at 2 days old (P2), and adult livers at 8 weeks old (8w). These livers were obtained from C57BL/6 wild-type mice. We used an anti-CYP3A4 antibody (ab3572), which is known as an antibody that has cross-reactivity among human, mouse, and rat. White broken lines surround magnified images shown in the bottom panels. Afp^{-} Alb^{+} cells that do not express Cyp3a11 and Cyp3a13 are observed in E16.5 and P2 mouse livers. DNA was stained with DAPI. Scale bars, 25 μm.

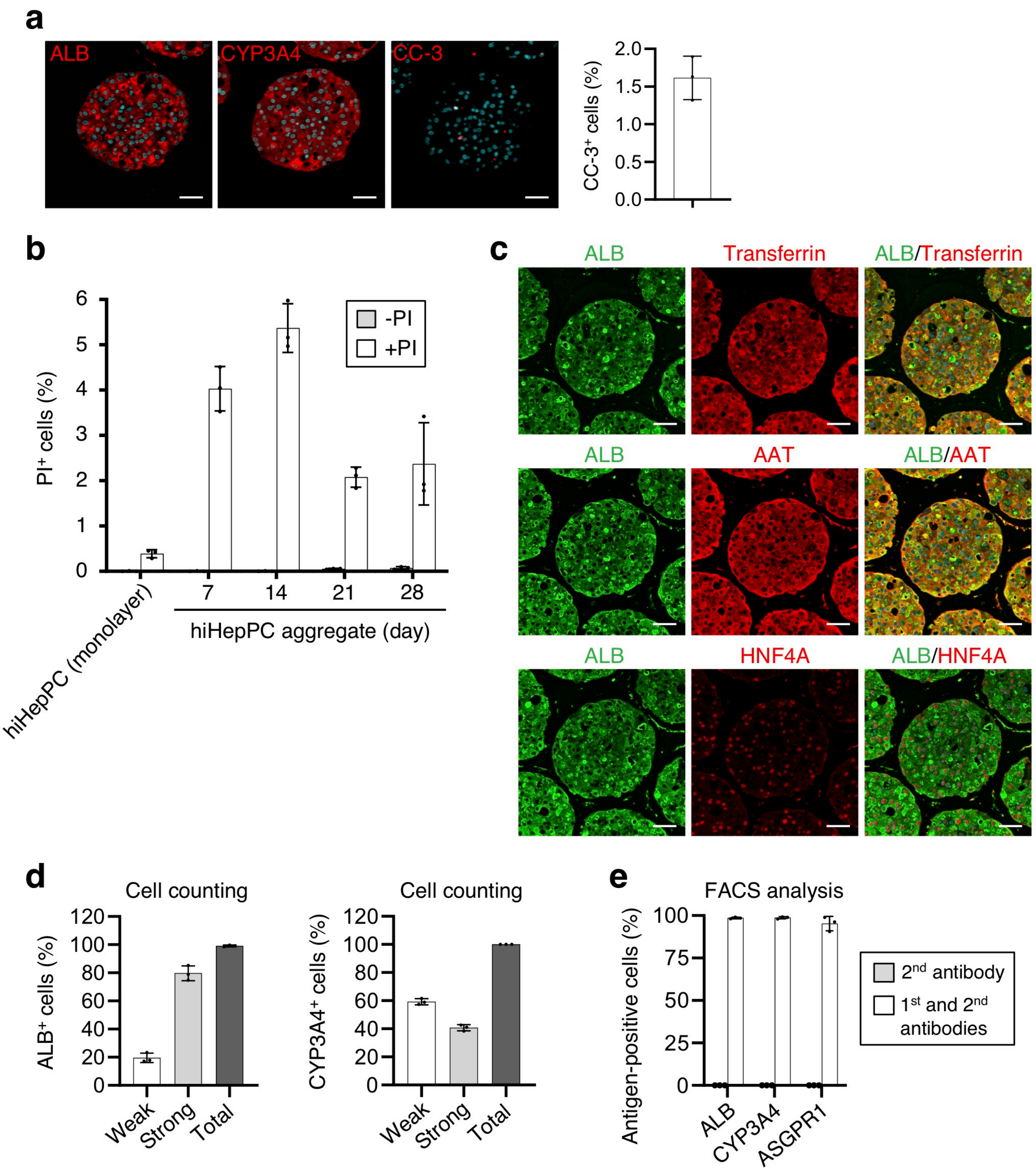

Supplementary Figure 5. Further characterization of hiHepPC aggregates. (**a**) Immunofluorescence staining of ALB, CYP3A4, or cleaved caspase 3 (CC-3) was conducted for hiHepPC aggregates at day 28 after initiation of 3D culture. The percentage of CC-3+ cells observed in hiHepPC aggregates was calculated after counting cells in 10 aggregates. (**b**) The percentage of propidium iodide+ (PI+) dead cells contained in hiHepPC monolayer cultures and hiHepPC aggregates at days 7, 14, 21, and 28 after initiation of 3D culture was evaluated by flow cytometry. (**c**) Co-immunofluorescence staining of ALB with transferrin, AAT, or HNF4A were conducted for hiHepPC aggregates at day 7 after initiation of 3D culture. (**d**) The percentages of cells that were weakly or strongly positive for immunofluorescence staining of ALB or CYP3A4 among cells composing hiHepPC aggregates at day 21 after initiation of 3D culture were calculated after counting cells in 10 aggregates. (**e**) The percentages of cells immunoreactive for ALB, CYP3A4, or ASGPR1 among cells composing hiHepPC aggregates at day 7 after initiation of 3D culture were evaluated by flow cytometry. Data represent the mean ± SD (*n* = 3 independent experiments). DNA was stained with DAPI. Scale bars, 50 μm. Source data are provided as a Source Data file.

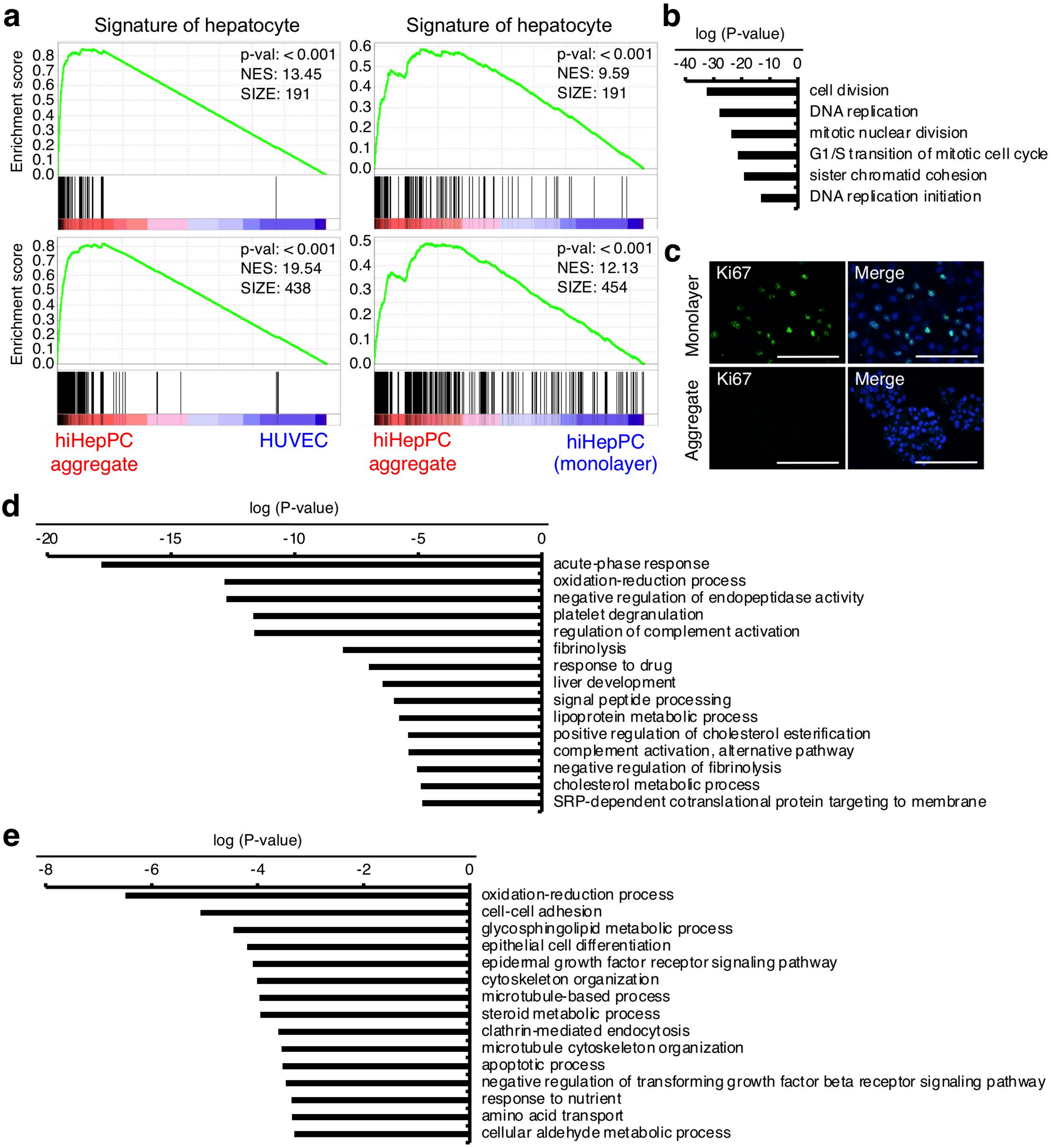

Supplementary Figure 6. Comparative analyses using the global gene expression profiles of HUVECs, hiHepPC monolayer cultures, hiHepPC aggregates, and hepatocytes. (**a**) GSEA of CEL-seq2 data for hiHepPC aggregates and HUVECs and of those for hiHepPC aggregates and hiHepPC monolayer cultures were performed using the set of top 200 (upper panels) and 500 (lower panels) genes specifically upregulated in human hepatocytes. (**b**) GOEA was performed for genes with expression levels higher in hiHepPC monolayer cultures than in hiHepPC aggregates. (**c**) Immunofluorescence staining of Ki67 was conducted for hiHepPCs in monolayer culture and cell aggregates derived from hiHepPCs at day 7 after initiation of 3D culture. DNA was stained with DAPI. Scale bars, 100 μm. (**d**) GOEA was performed for genes with expression levels higher in human hepatocytes than in hiHepPC aggregates. (**e**) GOEA was performed for genes with expression levels higher in hiHepPC aggregates than in human hepatocytes. Source data are provided as a Source Data file.

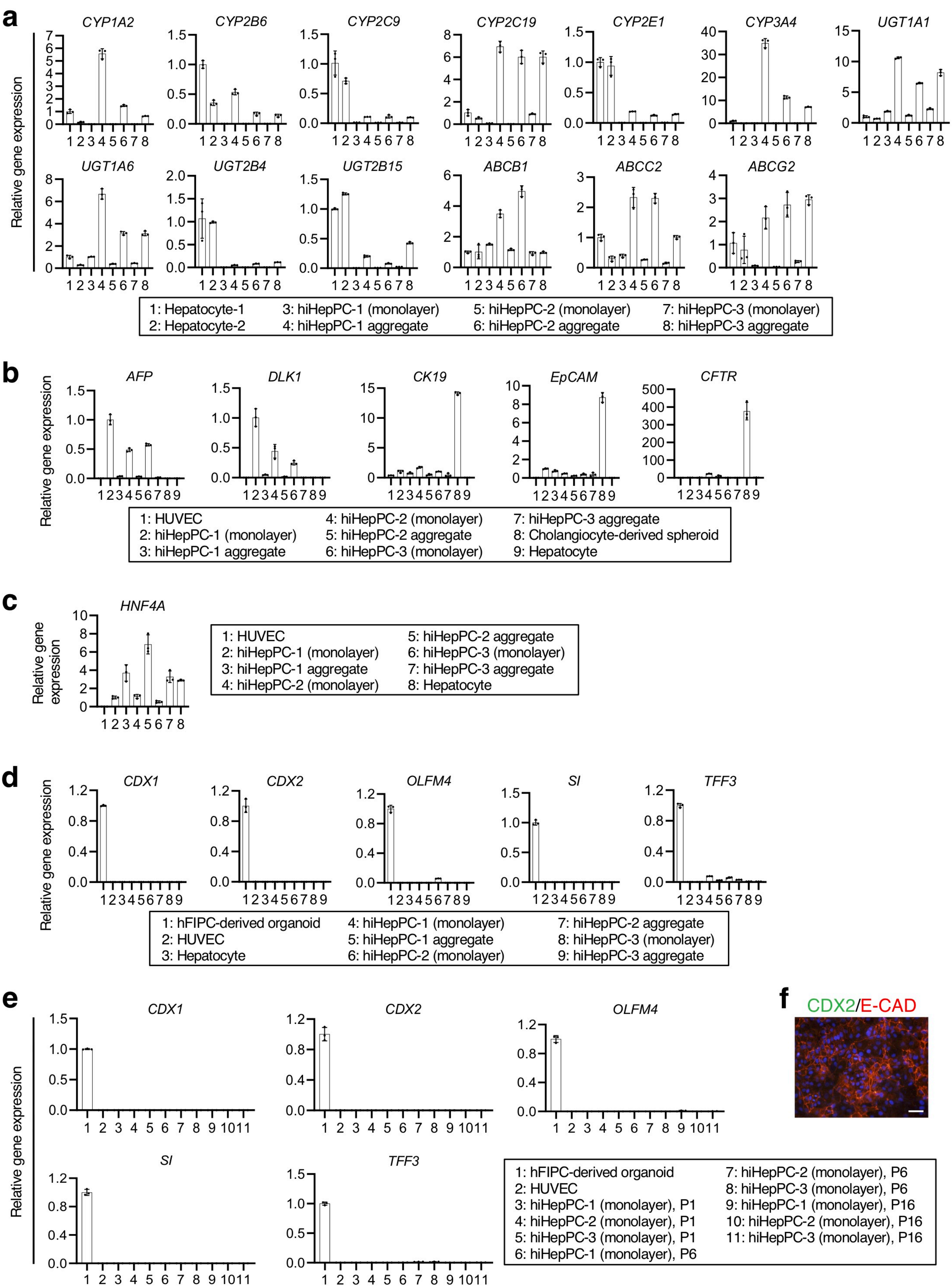
a
CYP1A2
CYP2B6
CYP2C9
CYP2C19
CYP2E1
CYP3A4
UGT1A1
UGT1A6
UGT2B4
UGT2B15
ABCB1
ABCC2
ABCG2
Relative gene expression
1: Hepatocyte-1
2: Hepatocyte-2
3: hiHepPC-1 (monolayer)
4: hiHepPC-1 aggregate
5: hiHepPC-2 (monolayer)
6: hiHepPC-2 aggregate
7: hiHepPC-3 (monolayer)
8: hiHepPC-3 aggregate
b
AFP
DLK1
CK19
EpCAM
CFTR
Relative gene expression
1: HUVEC
2: hiHepPC-1 (monolayer)
3: hiHepPC-1 aggregate
4: hiHepPC-2 (monolayer)
5: hiHepPC-2 aggregate
6: hiHepPC-3 (monolayer)
7: hiHepPC-3 aggregate
8: Cholangiocyte-derived spheroid
9: Hepatocyte
c
HNF4A
Relative gene expression
1: HUVEC
2: hiHepPC-1 (monolayer)
3: hiHepPC-1 aggregate
4: hiHepPC-2 (monolayer)
5: hiHepPC-2 aggregate
6: hiHepPC-3 (monolayer)
7: hiHepPC-3 aggregate
8: Hepatocyte
d
CDX1
CDX2
OLFM4
SI
TFF3
Relative gene expression
1: hFIPC-derived organoid
2: HUVEC
3: Hepatocyte
4: hiHepPC-1 (monolayer)
5: hiHepPC-1 aggregate
6: hiHepPC-2 (monolayer)
7: hiHepPC-2 aggregate
8: hiHepPC-3 (monolayer)
9: hiHepPC-3 aggregate
e
CDX1
CDX2
OLFM4
SI
TFF3
Relative gene expression
1: hFIPC-derived organoid
2: HUVEC
3: hiHepPC-1 (monolayer), P1
4: hiHepPC-2 (monolayer), P1
5: hiHepPC-3 (monolayer), P1
6: hiHepPC-1 (monolayer), P6
7: hiHepPC-2 (monolayer), P6
8: hiHepPC-3 (monolayer), P6
9: hiHepPC-1 (monolayer), P16
10: hiHepPC-2 (monolayer), P16
11: hiHepPC-3 (monolayer), P16
f
CDX2/E-CAD

Supplementary Figure 7. Gene expression analyses of hiHepPCs in monolayer culture and cell aggregates derived from hiHepPCs. (**a**) qPCR analyses of genes encoding liver enzymes were performed on total RNA obtained from human hepatocytes derived from two different donors, three different hiHepPCs in monolayer culture, and cell aggregates derived from these hiHepPCs at day 21 after initiation of 3D culture. All data were normalized with the values for hepatocytes in the left-most lane in each graph, and the fold differences are shown. (**b**) qPCR analyses of hHepPC marker genes (*AFP* and *DLK1*) and cholangiocyte marker genes (*CK19*, *EpCAM*, and *CFTR*) were performed on total RNA obtained from HUVECs, three different hiHepPCs in monolayer culture, cell aggregates derived from these hiHepPCs at day 21 after initiation of 3D culture, human fetal cholangiocyte-derived spheroids at passage 3 after initiation of 3D culture, and human hepatocytes. All data were normalized with the values for hiHepPC-1 (monolayer), and the fold differences are shown. (**c**) qPCR analyses of *HNF4A* were performed on total RNA obtained from HUVECs, three different hiHepPCs in monolayer culture, cell aggregates derived from these hiHepPCs at day 21 after initiation of 3D culture, and human hepatocytes. All data were normalized with the values for hiHepPC-1 (monolayer), and the fold differences are shown. (**d**) qPCR analyses of intestinal epithelial cell marker genes were performed on total RNA obtained from spherical organoids derived from human fetal intestinal progenitor cells (hFIPCs)[5], HUVECs, human hepatocytes, three different hiHepPCs in monolayer culture, and cell aggregates derived from these hiHepPCs at day 21 after initiation of 3D culture. All data were normalized with the values for hFIPC-derived organoids, and the fold differences are shown. (**e**) qPCR analyses of intestinal epithelial cell marker genes were performed on total RNA obtained from spherical organoids derived from human fetal intestinal progenitor cells (hFIPCs)[5], HUVECs, and three different hiHepPCs at passage (P) 1, P6, and P16 in monolayer culture. All data were normalized with the values for hFIPC-derived organoids, and the fold differences are shown. (**f**) Co-immunofluorescence staining of CDX2 with E-CAD was conducted for hiHepPCs at passage 6 in monolayer culture. DNA was stained with DAPI. Scale bar, 50 μm. Data represent the mean ± SD (*n* = 3 independent assays). Source data are provided as a Source Data file.

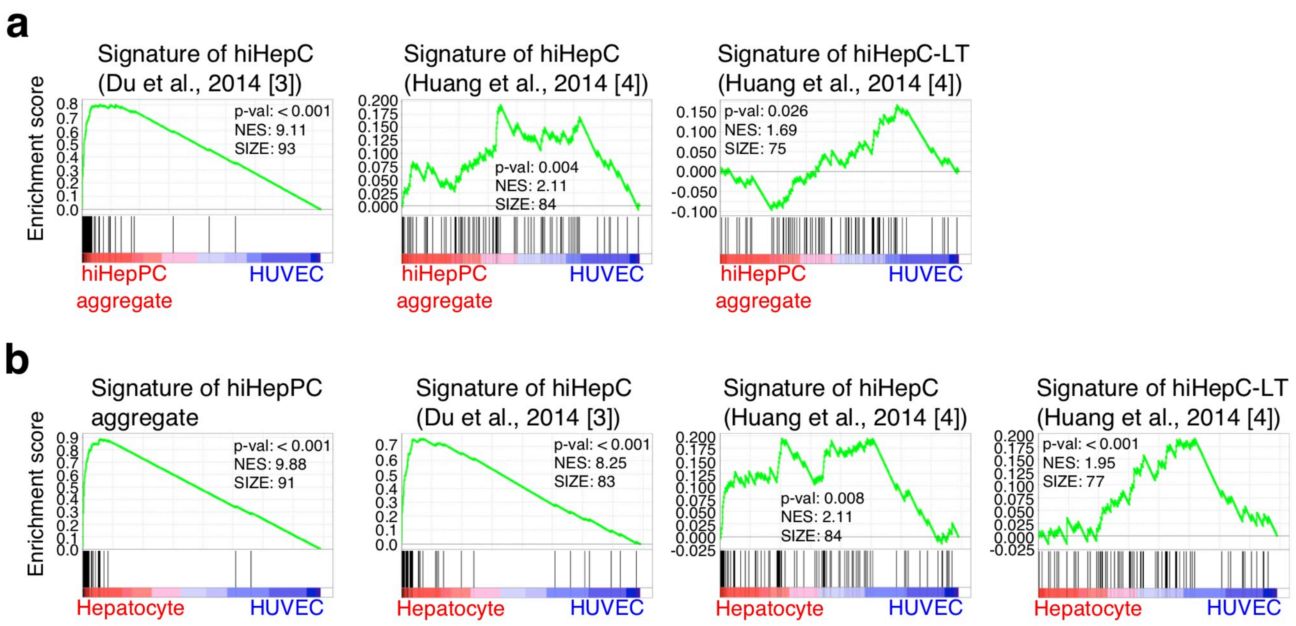

Supplementary Figure 8. The gene expression signature of hiHepPC aggregates is similar to those of hiHepC-Du and human hepatocytes. (**a**,**b**) GSEA of CEL-seq2 data for hiHepPC aggregates and HUVECs (**a**) and of those for human hepatocytes and HUVECs (**b**) were performed using the set of top 100 genes specifically upregulated in hiHepC-Du, hiHepC-Huang, hiHepC-LT-Huang, or hiHepPC aggregates.

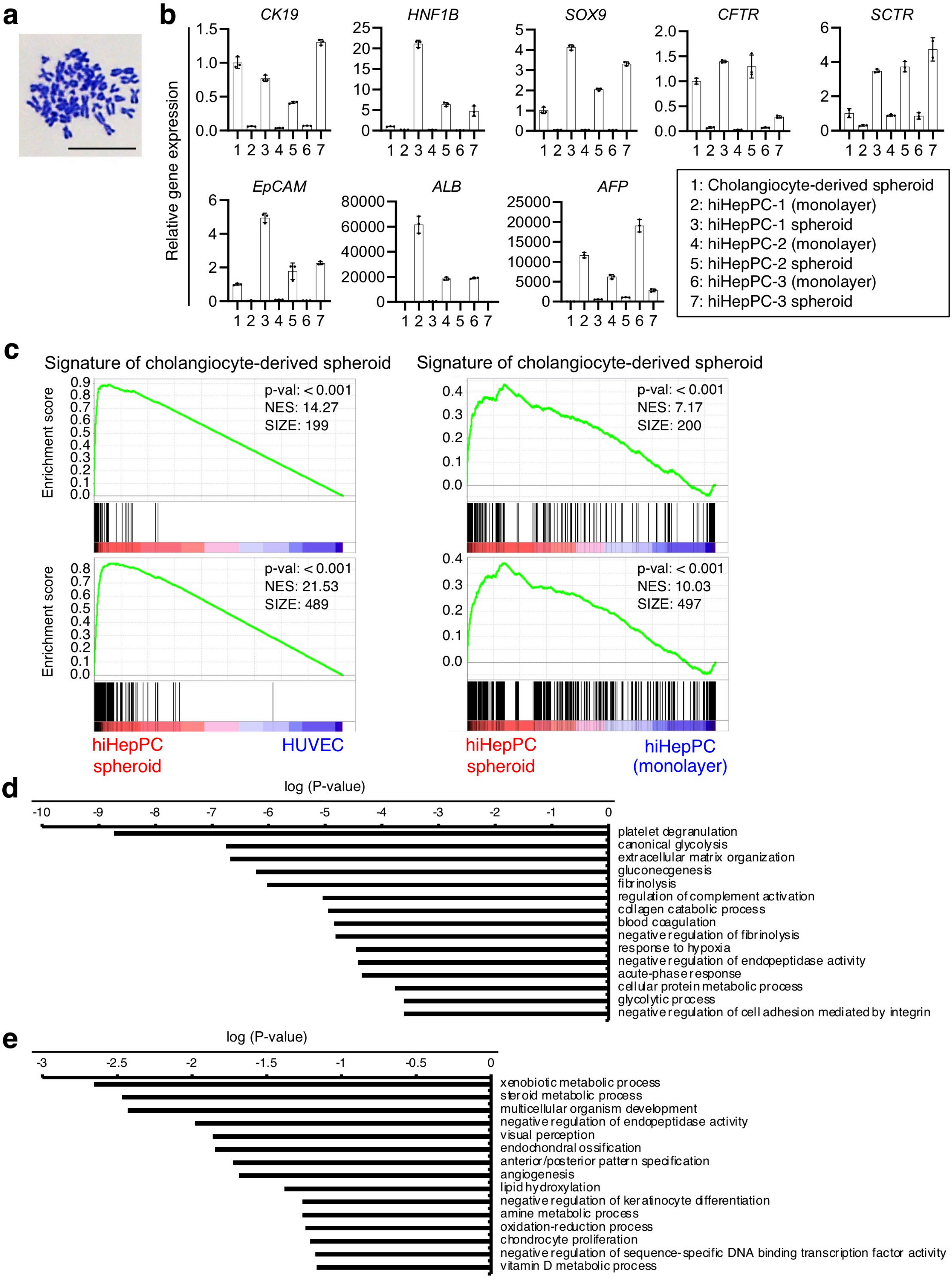

a
b
Relative gene expression
CK19
HNF1B
SOX9
CFTR
SCTR
EpCAM
ALB
AFP
1: Cholangiocyte-derived spheroid
2: hiHepPC-1 (monolayer)
3: hiHepPC-1 spheroid
4: hiHepPC-2 (monolayer)
5: hiHepPC-2 spheroid
6: hiHepPC-3 (monolayer)
7: hiHepPC-3 spheroid
c
Signature of cholangiocyte-derived spheroid
Enrichment score
p-val: < 0.001
NES: 14.27
SIZE: 199
p-val: < 0.001
NES: 21.53
SIZE: 489
hiHepPC spheroid
HUVEC
Signature of cholangiocyte-derived spheroid
p-val: < 0.001
NES: 7.17
SIZE: 200
p-val: < 0.001
NES: 10.03
SIZE: 497
hiHepPC spheroid
hiHepPC (monolayer)
d
log (P-value)
platelet degranulation
canonical glycolysis
extracellular matrix organization
gluconeogenesis
fibrinolysis
regulation of complement activation
collagen catabolic process
blood coagulation
negative regulation of fibrinolysis
response to hypoxia
negative regulation of endopeptidase activity
acute-phase response
cellular protein metabolic process
glycolytic process
negative regulation of cell adhesion mediated by integrin
e
log (P-value)
xenobiotic metabolic process
steroid metabolic process
multicellular organism development
negative regulation of endopeptidase activity
visual perception
endochondral ossification
anterior/posterior pattern specification
angiogenesis
lipid hydroxylation
negative regulation of keratinocyte differentiation
amine metabolic process
oxidation-reduction process
chondrocyte proliferation
negative regulation of sequence-specific DNA binding transcription factor activity
vitamin D metabolic process

Supplementary Figure 9. Differentiation of hiHepPCs into genome-stable cholangiocytes. (**a**) A representative image of a karyotype of a hiHepPC-derived cholangiocyte constituting a spheroid at passage 8 after initiation of 3D culture with Matrigel. Note that the numbers of chromosomes in all 20 hiHepPC-derived cholangiocytes analyzed in this study were normal. Scale bar, 10 μm. (**b**) qPCR analyses of cholangiocyte marker genes were performed on total RNA obtained from human fetal cholangiocyte-derived spheroids, three different hiHepPCs in monolayer culture, and spheroids derived from these hiHepPCs. The spheroids derived from cholangiocytes and hiHepPCs were used at passage 2 and 9, respectively, after initiation of 3D culture with Matrigel. All data were normalized with the values for cholangiocyte-derived spheroids, and the fold differences are shown. Data represent the mean $\pm$ SD (n = 3 independent assays). (**c**) GSEA of CEL-seq2 data for hiHepPC spheroids and HUVECs and of those for hiHepPC spheroids and hiHepPC monolayer cultures were performed using the set of top 200 (upper panels) and 500 (lower panels) genes specifically upregulated in human fetal cholangiocyte-derived spheroids. (**d**) GOEA was performed for genes with expression levels higher in human fetal cholangiocyte-derived spheroids than in hiHepPC spheroids. (**e**) GOEA was performed for genes with expression levels higher in hiHepPC spheroids than in human fetal cholangiocyte-derived spheroids. Source data are provided as a Source Data file.

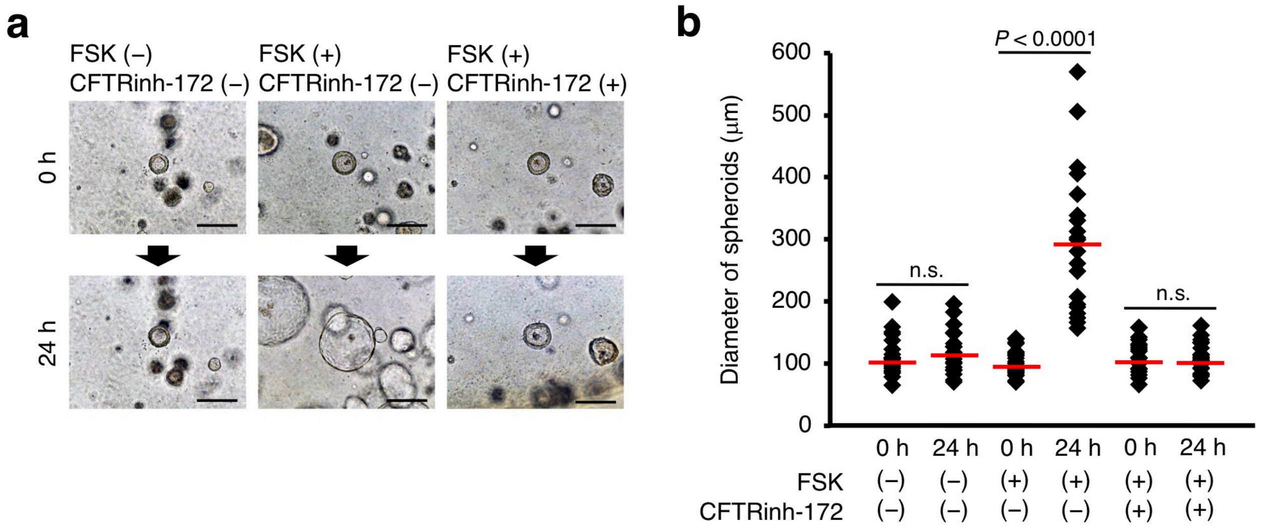

Supplementary Figure 10. hiHepPC spheroids are composed of functional cholangiocytes. (**a**) Representative morphologies of hiHepPC spheroids before and after culture with or without FSK and/or CFTRinh-172 for 24 h. Scale bars, 100 μm. (**b**) The diameter of 20 spheroids in each assay point of the indicated culture conditions was measured. Means of the data are shown with red lines (n = 20 spheroids). Statistical difference was determined by one-way analysis of variance followed by Tukey–Kramer test. n.s., not significant. Source data are provided as a Source Data file.

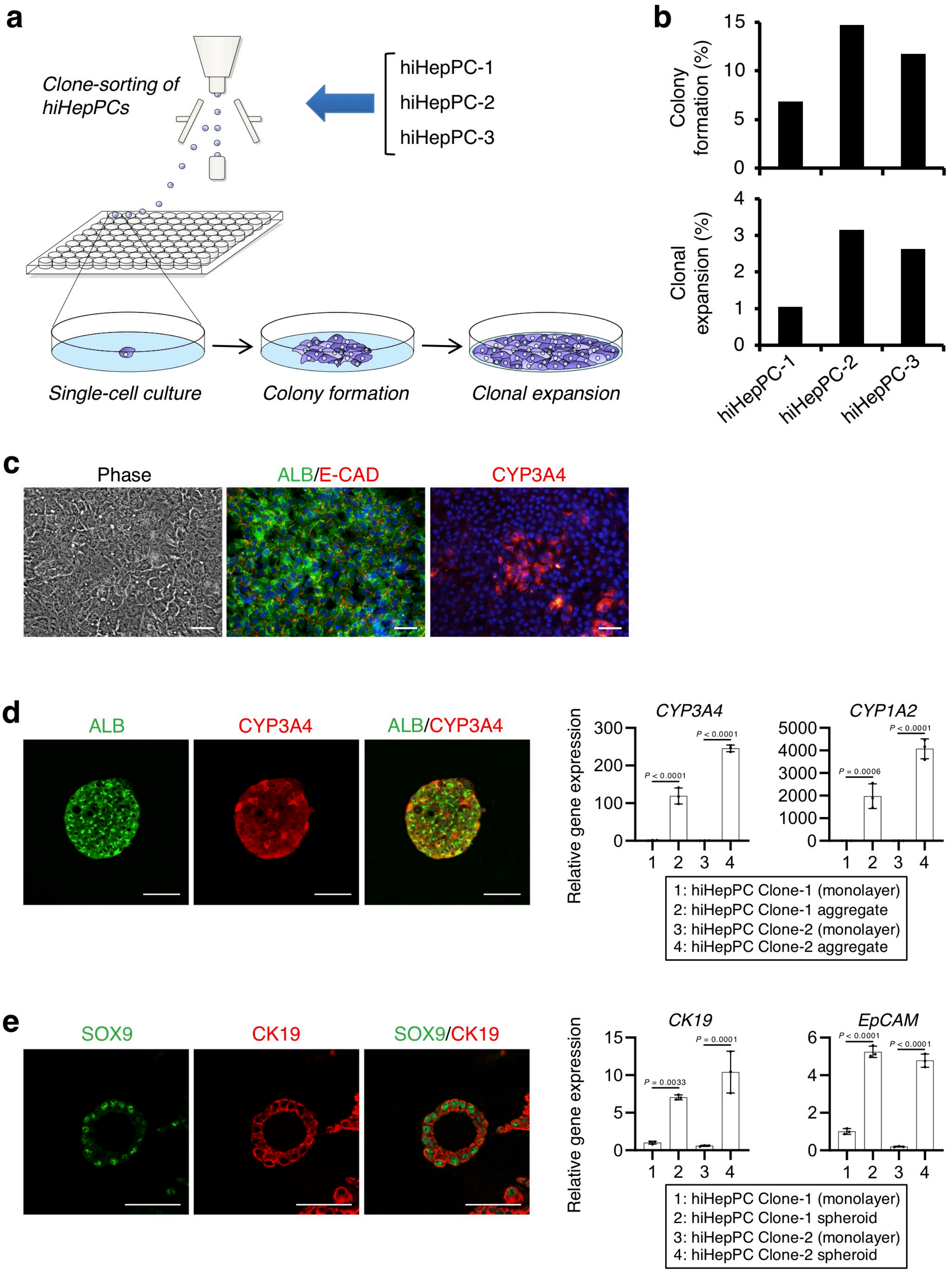

a
Clone-sorting of hiHepPCs
hiHepPC-1
hiHepPC-2
hiHepPC-3
Single-cell culture
Colony formation
Clonal expansion
b
Colony formation (%)
Clonal expansion (%)
hiHepPC-1
hiHepPC-2
hiHepPC-3
c
Phase
ALB/E-CAD
CYP3A4
d
ALB
CYP3A4
ALB/CYP3A4
Relative gene expression
CYP3A4
CYP1A2
P < 0.0001
P = 0.0006
1: hiHepPC Clone-1 (monolayer)
2: hiHepPC Clone-1 aggregate
3: hiHepPC Clone-2 (monolayer)
4: hiHepPC Clone-2 aggregate
e
SOX9
CK19
SOX9/CK19
Relative gene expression
CK19
EpCAM
P = 0.0033
P = 0.0001
P < 0.0001
1: hiHepPC Clone-1 (monolayer)
2: hiHepPC Clone-1 spheroid
3: hiHepPC Clone-2 (monolayer)
4: hiHepPC Clone-2 spheroid

Supplementary Figure 11. Clonal analysis of HUVEC-derived hiHepPCs. (**a**) Experimental procedure for the clonal analyses of three different hiHepPCs. Cells in each hiHepPC monolayer culture at passages 10–13 underwent clone sorting by flow cytometry and were clonally cultured in 96-well plates. A portion of hiHepPCs formed colonies and expanded in clonal culture. (**b**) The percentages of hiHepPC clones that formed colonies in the wells of 96-well plates (upper graph) and propagated over a prolonged culture period (lower graph) at day 21 and passage 4, respectively, after clone sorting of three different hiHepPCs. (**c**) Co-immunofluorescence staining of ALB with E-CAD and immunofluorescence staining of CYP3A4 were conducted for a hiHepPC clone propagating in monolayer culture. Representative morphology and fluorescence image of the hiHepPC clone are shown. (**d,e**) Co-immunofluorescence staining of ALB with CYP3A4 (**d**) and of SOX9 with CK19 (**e**) were conducted for hiHepPC aggregates and spheroids, respectively, that were derived from a representative hiHepPC clone. Also, qPCR analyses of hepatocyte marker genes (*CYP3A4* and *CYP1A2*) (**d**) and cholangiocyte marker genes (*CK19* and *EpCAM*) (**e**) were performed on total RNA obtained from two different hiHepPC clones in monolayer culture and cell aggregates/spheroids derived from these hiHepPC clones. All data were normalized with the values for hiHepPC Clone-1 (monolayer), and the fold differences are shown. Statistical difference was determined by one-way analysis of variance followed by Tukey–Kramer test. Data represent the mean ± SD (n = 3 independent assays). DNA was stained with DAPI. Scale bars, 50 μm. Source data are provided as a Source Data file.

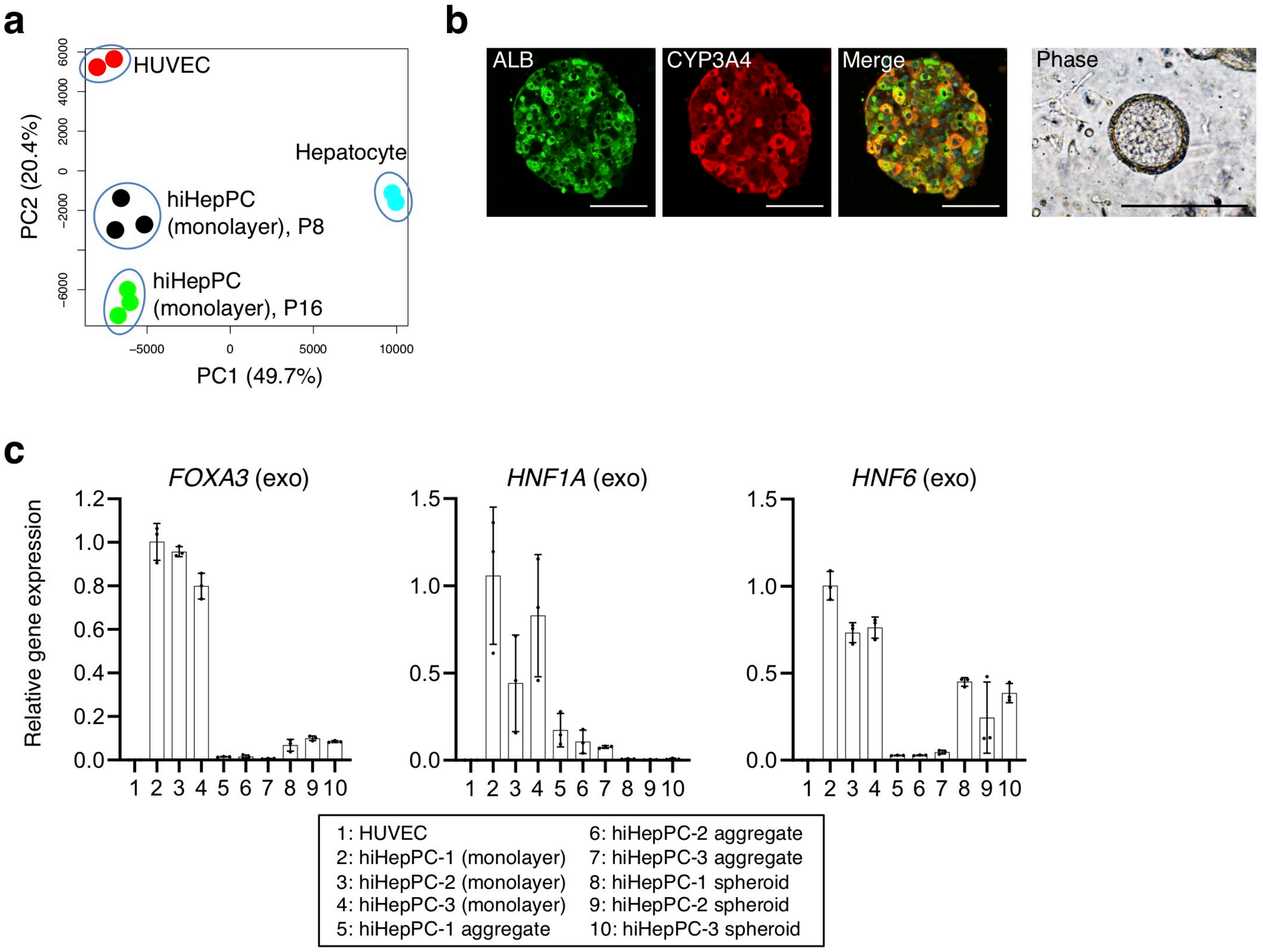

Supplementary Figure 12. Stable expansion of hiHepPCs in monolayer culture and retroviral gene silencing in hiHepPC aggregates and spheroids under 3D culture conditions. (**a**) PCA was performed using CEL-seq2 data for HUVECs, hiHepPCs at passage (P) 8 and P16 in monolayer culture, and human hepatocytes. (**b**) Representative fluorescence images and morphology of a cell aggregate composed of cells immunoreactive for ALB with CYP3A4 and a cystic spheroid, respectively, both of which were formed from hiHepPCs at passage 16 in monolayer culture. DNA was stained with DAPI. Scale bars, 50 μm. (**c**) qPCR analyses of retroviral exogenous gene expression of *FOXA3*, *HNF1A*, and *HNF6* were performed on total RNA obtained from HUVECs, three different hiHepPCs at passage (P) 8 in monolayer culture, cell aggregates derived from these hiHepPCs at day 21 after initiation of 3D culture, and spheroids derived from these hiHepPCs at P7 after initiation of 3D culture. All data were normalized with the values for hiHepPC-1 (monolayer), and the fold differences are shown. Data represent the mean ± SD (n = 3 independent assays). Source data are provided as a Source Data file.

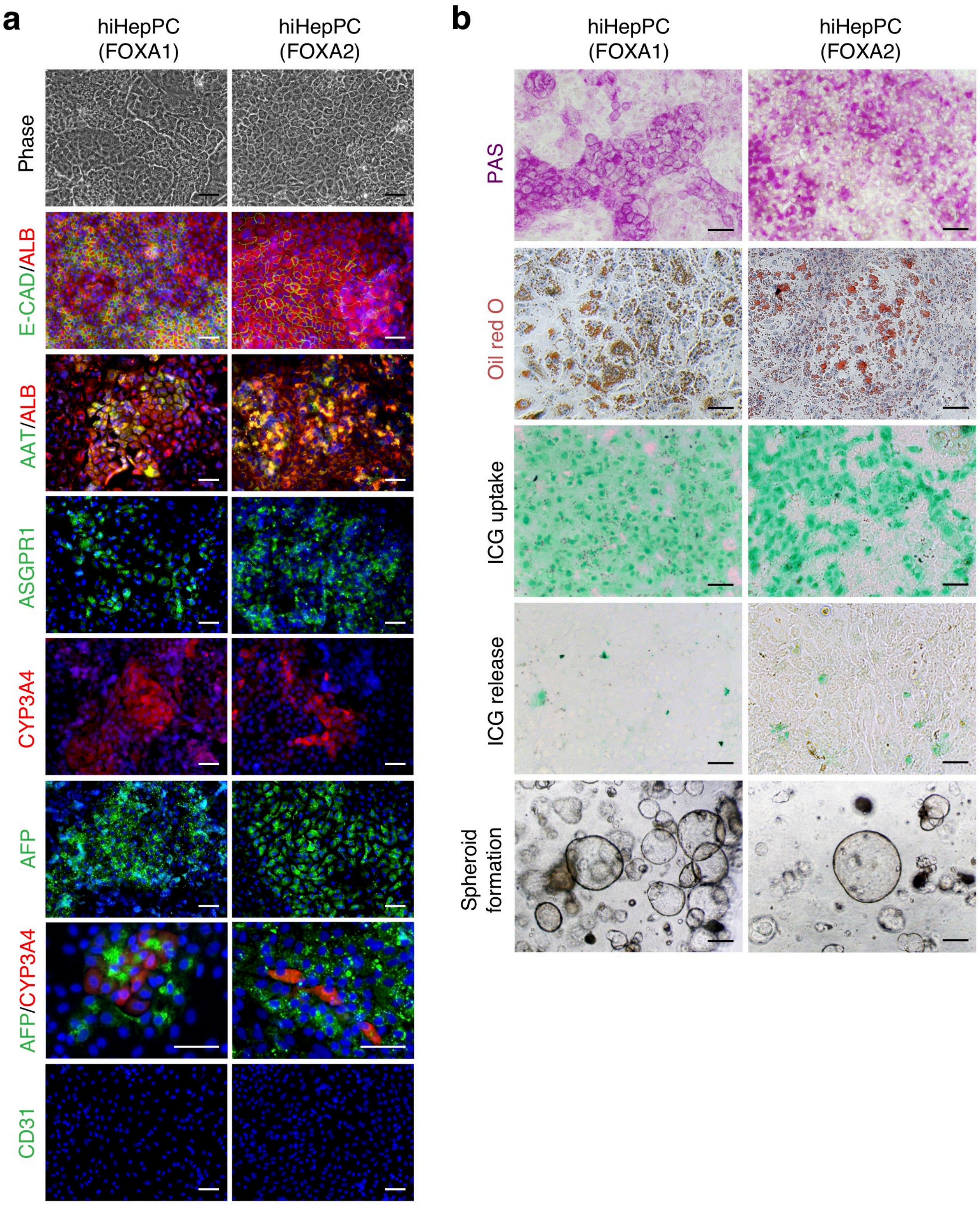

Supplementary Figure 13. Redundant function of FOXA transcription factors in the generation of hiHepPCs. (**a**) Co-immunofluorescence staining of ALB with E-CAD or AAT and of AFP with CYP3A4 and immunofluorescence staining of ASGPR1, CYP3A4, AFP, and CD31 were conducted for hiHepPCs induced by the forced expression of *HNF1A* and *HNF6* with *FOXA1* or *FOXA2* in HUVECs, which were designated hiHepPC (FOXA1) and hiHepPC (FOXA2), respectively. Representative morphologies and fluorescence images of these two types of hiHepPCs are shown. DNA was stained with DAPI. (**b**) PAS staining and oil red O staining were conducted for hiHepPC (FOXA1) and hiHepPC (FOXA2). These hiHepPCs incorporated and excreted ICG and formed cystic epithelial spheroids in 3D culture with Matrigel. Scale bars, 50 μm.

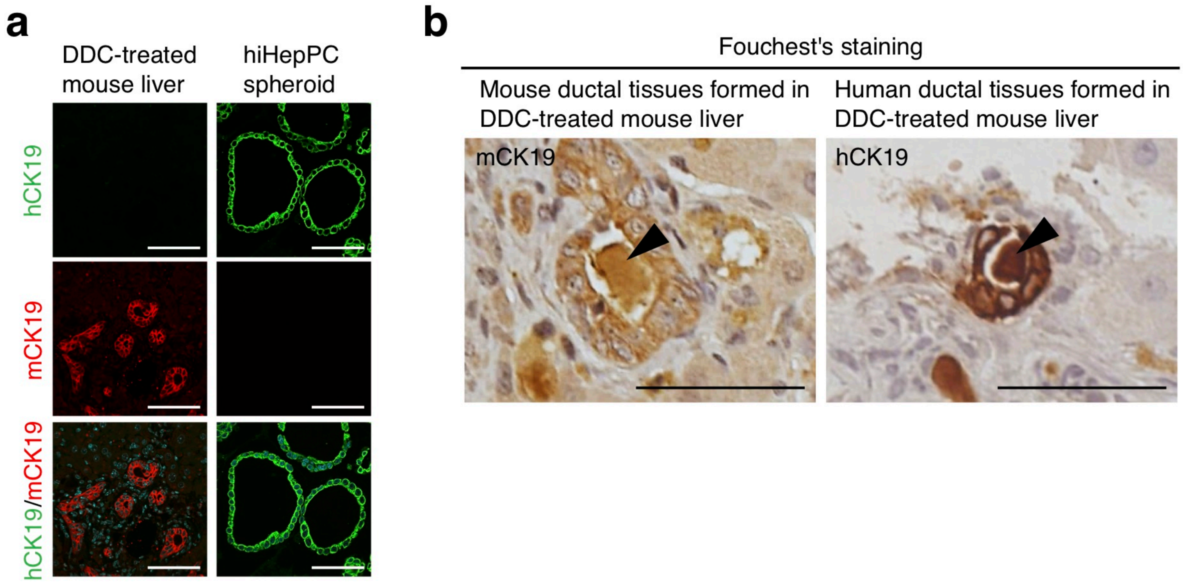

Supplementary Figure 14. hiHepPC-derived cholangiocytes form functional biliary ductal tissues after transplantation into the injured mouse liver. (**a**) Co-immunofluorescence staining of hCK19 with mCK19 was conducted for DDC-treated mouse livers and hiHepPC spheroids. Note that no cross-reactivity was observed. DNA was stained with DAPI. Scale bars, 50 μm. (**b**) Fouchest's staining was conducted with immunohistochemical staining of mCK19 or hCK19 for DDC-treated mouse livers (left image) or DDC-treated mouse livers 4 weeks after the last injection of cells dissociated from hiHepPC spheroids (right image), respectively. Arrowheads indicate accumulation of the bile pigment bilirubin in the mouse (left image) and human (right image) ductal tissues formed in the DDC-treated mouse livers. Nuclei were stained with hematoxylin. Scale bars, 25 μm.

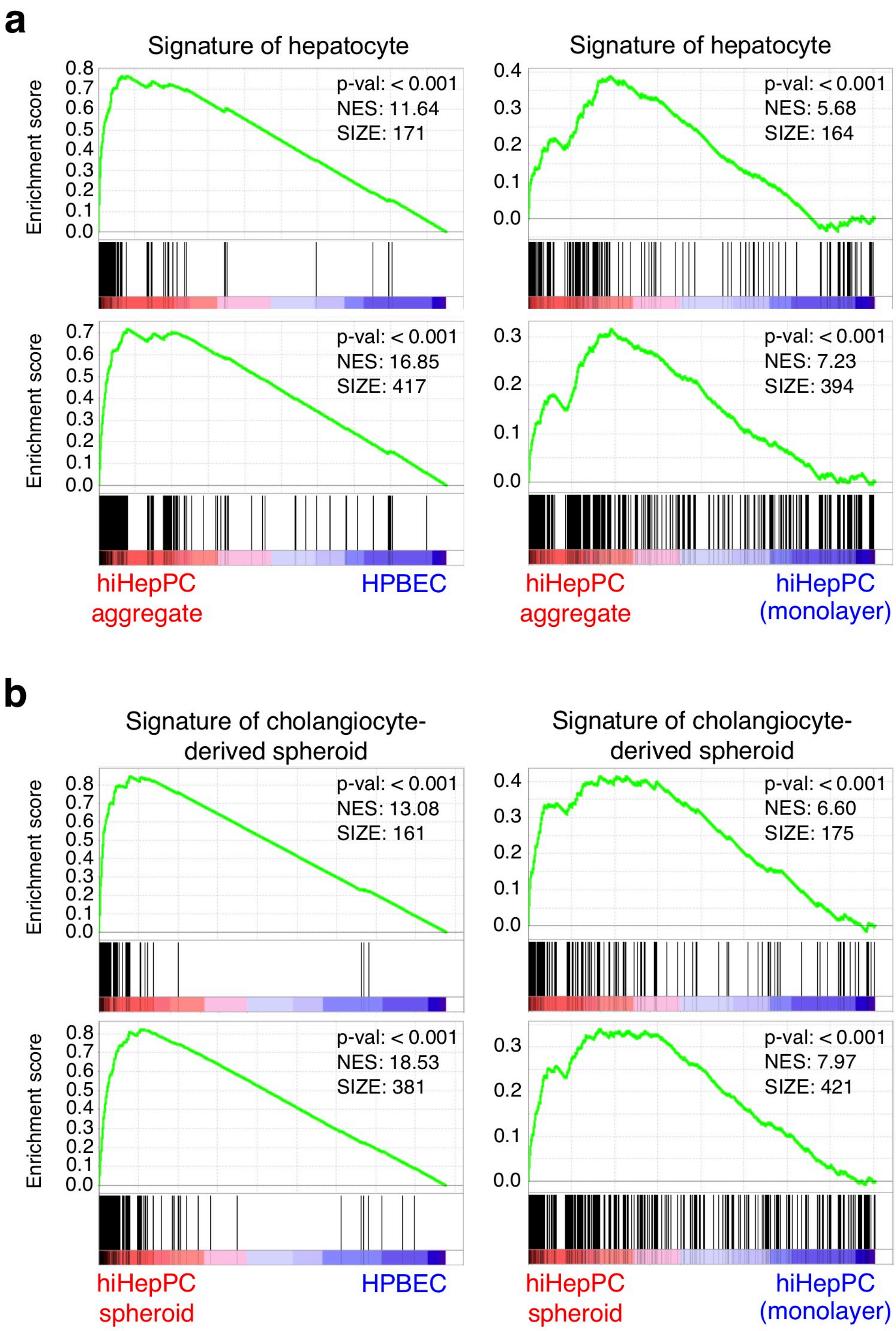

Supplementary Figure 15. Upregulation of human hepatocyte- and cholangiocyte-specific gene expression in HPBEC-derived hiHepPC aggregates and spheroids, respectively. (**a**) GSEA of CEL-seq2 data for hiHepPC aggregates and HPBECs and of those for hiHepPC aggregates and hiHepPC monolayer cultures were performed using the set of top 200 (upper panels) and 500 (lower panels) genes specifically upregulated in human hepatocytes. hiHepPCs used in these analyses were derived from HPBECs. (**b**) GSEA of CEL-seq2 data for hiHepPC spheroids and HPBECs and of those for hiHepPC spheroids and hiHepPC monolayer cultures were performed using the set of top 200 (upper panels) and 500 (lower panels) genes specifically upregulated in human fetal cholangiocyte-derived spheroids. hiHepPCs used in these analyses were derived from HPBECs.

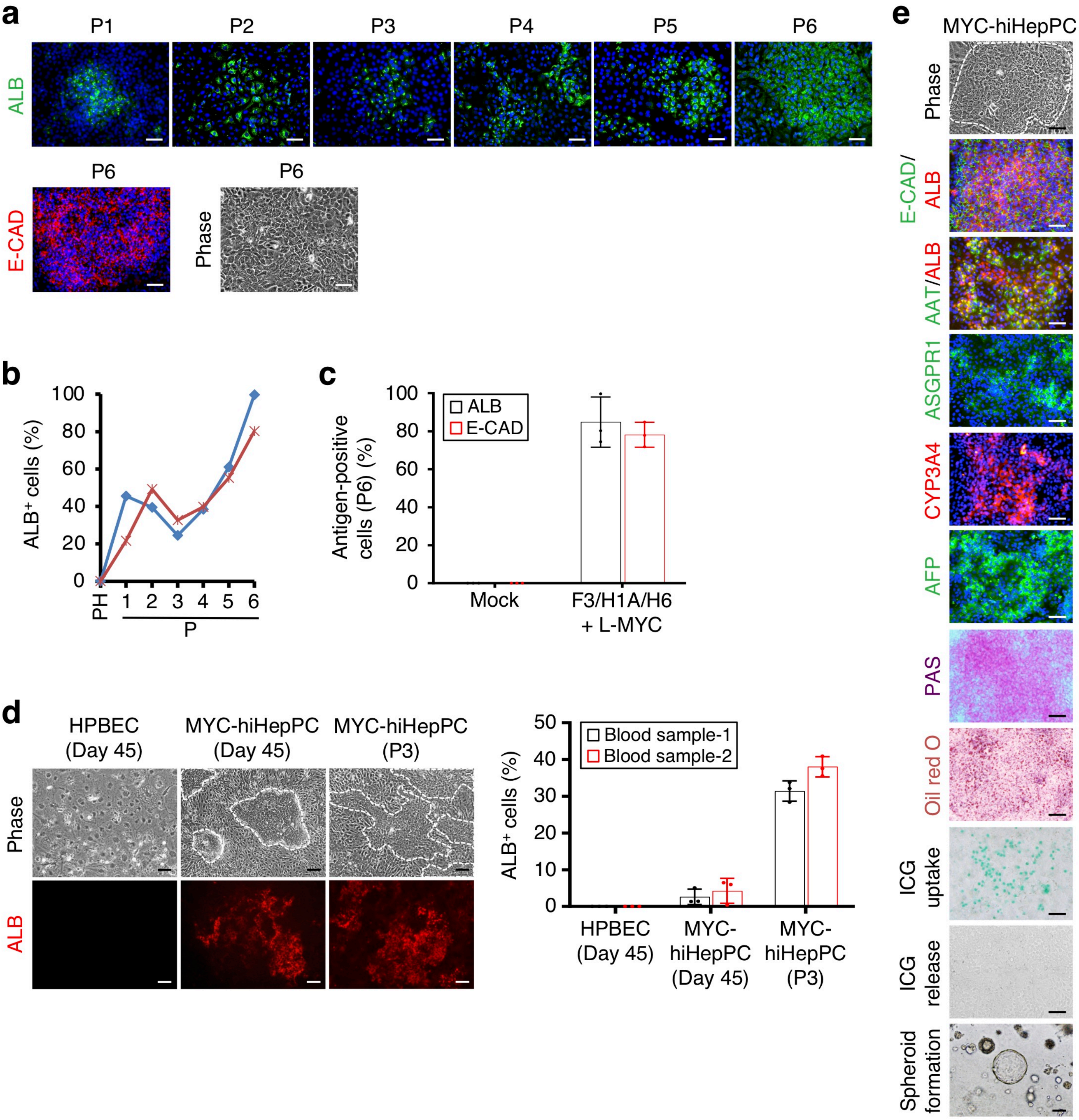
a
P1
P2
P3
P4
P5
P6
ALB
P6
E-CAD
P6
Phase
b
ALB+ cells (%)
100
80
60
40
20
0
PH
1
2
3
4
5
6
P
c
Antigen-positive cells (P6) (%)
ALB
E-CAD
Mock
F3/H1A/H6 + L-MYC
d
HPBEC (Day 45)
MYC-hiHepPC (Day 45)
MYC-hiHepPC (P3)
Phase
ALB
ALB+ cells (%)
50
40
30
20
10
0
Blood sample-1
Blood sample-2
HPBEC (Day 45)
MYC-hiHepPC (Day 45)
MYC-hiHepPC (P3)
e
MYC-hiHepPC
Phase
E-CAD/ALB
AAT/ALB
ASGPR1
CYP3A4
AFP
PAS
Oil red O
ICG uptake
ICG release
Spheroid formation

Supplementary Figure 16. L-MYC effectively promotes the induction of hiHepPCs from HUVECs and HPBECs. (**a**) Immunofluorescence staining of ALB or E-CAD was conducted for HUVECs transduced with *FOXA3*, *HNF1A*, *HNF6*, and *L-MYC* at the indicated passage numbers (P). Representative fluorescence images and morphologies of transduced HUVECs are shown. DNA was stained with DAPI. Scale bars, 50 μm. (**b**) The graph shows the percentages of ALB^+ cells observed in culture of HUVECs transduced with *FOXA3*, *HNF1A*, *HNF6*, and *L-MYC* at the indicated passage numbers (P). The data obtained from two independent experiments are shown by red and blue lines in the graph, respectively. PH, parental HUVEC. (**c**) The percentages of cells immunoreactive for ALB or E-CAD among mock-infected HUVECs and HUVECs transduced with *FOXA3*, *HNF1A*, *HNF6*, and *L-MYC* at passage (P) 6. FOXA3, HNF1A, and HNF6 are abbreviated as F3, H1A, and H6, respectively. Data represent the mean ± SD (n = 3 independent experiments). (**d**) Immunofluorescence staining of ALB was conducted for mock-infected HPBECs at day 45 and HPBEC-derived MYC-hiHepPCs at day 45 and passage (P) 3 after retrovirus infection. Representative morphologies and fluorescence images of these two types of cells are shown. White broken lines surround MYC-hiHepPC colonies. Scale bars, 100 μm. The graph shows the percentages of ALB^+ cells observed in individual cultures of HPBECs at day 45 and MYC-hiHepPCs at day 45 and P3 after retrovirus infection. The data obtained from two biologically independent experiments using different blood samples are shown in the graph. Data represent the mean ± SD (n = 3 independent assays). (**e**) Co-immunofluorescence staining of ALB with E-CAD or AAT; immunofluorescence staining of ASGPR1, CYP3A4, and AFP; PAS staining; and oil red O staining were conducted for HPBEC-derived MYC-hiHepPCs. Representative morphologies of these cells are also shown. White broken line surrounds a MYC-hiHepPC colony. In addition, HPBEC-derived MYC-hiHepPCs incorporated and excreted ICG and formed cystic epithelial spheroids in 3D culture with Matrigel. DNA was stained with DAPI. Scale bars, 50 μm. Source data are provided as a Source Data file.

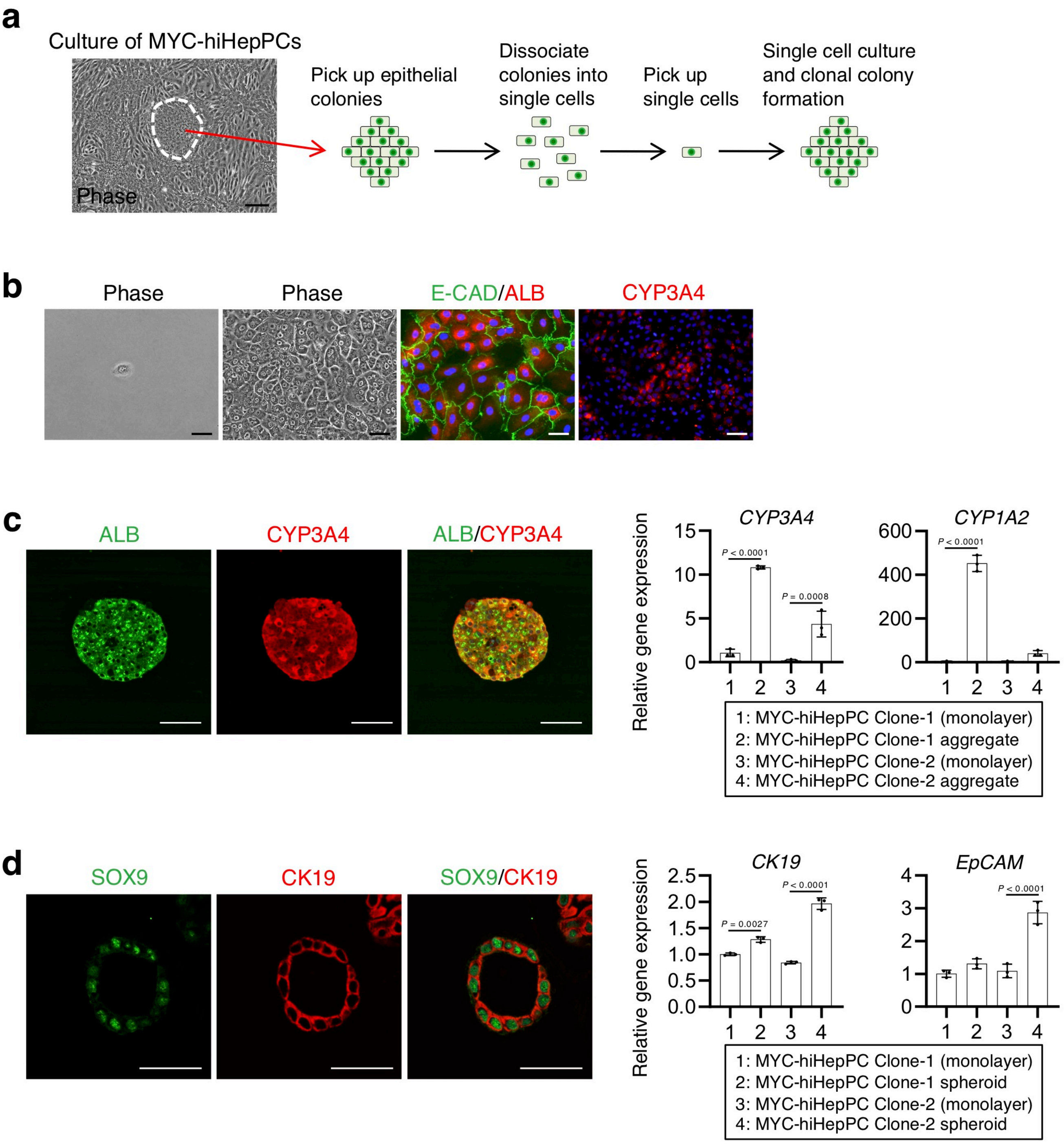

Supplementary Figure 17. Clonal analysis of HPBEC-derived MYC-hiHepPCs. (**a**) Schematic diagram of the experimental procedure. White broken line surrounds a HPBEC-derived MYC-hiHepPC colony. Scale bar, 100 μm. (**b**) Representative morphology of a single cell picked from dissociated colony cells and cultured in an individual well of 96-well plates and of a MYC-hiHepPC clone propagating in culture. Also, co-immunofluorescence staining of E-CAD with ALB and immunofluorescence staining of CYP3A4 were conducted for a MYC-hiHepPC clone propagating in culture. DNA was stained with DAPI. Scale bars, 50 μm. (**c,d**) Co-immunofluorescence staining of ALB with CYP3A4 (**c**) and of SOX9 with CK19 (**d**) were conducted for MYC-hiHepPC aggregates and spheroids, respectively, that were derived from a representative MYC-hiHepPC clone. DNA was stained with DAPI. Scale bars, 50 μm. Also, qPCR analyses of hepatocyte marker genes (*CYP3A4* and *CYP1A2*) (**c**) and cholangiocyte marker genes (*CK19* and *EpCAM*) (**d**) were performed on total RNA obtained from two different MYC-hiHepPC clones in monolayer culture and cell aggregates/spheroids derived from these MYC-hiHepPC clones. All data were normalized with the values for MYC-hiHepPC Clone-1 (monolayer), and the fold differences are shown. Statistical difference was determined by one-way analysis of variance followed by Tukey–Kramer test. Data represent the mean ± SD (n = 3 independent assays). Source data are provided as a Source Data file.

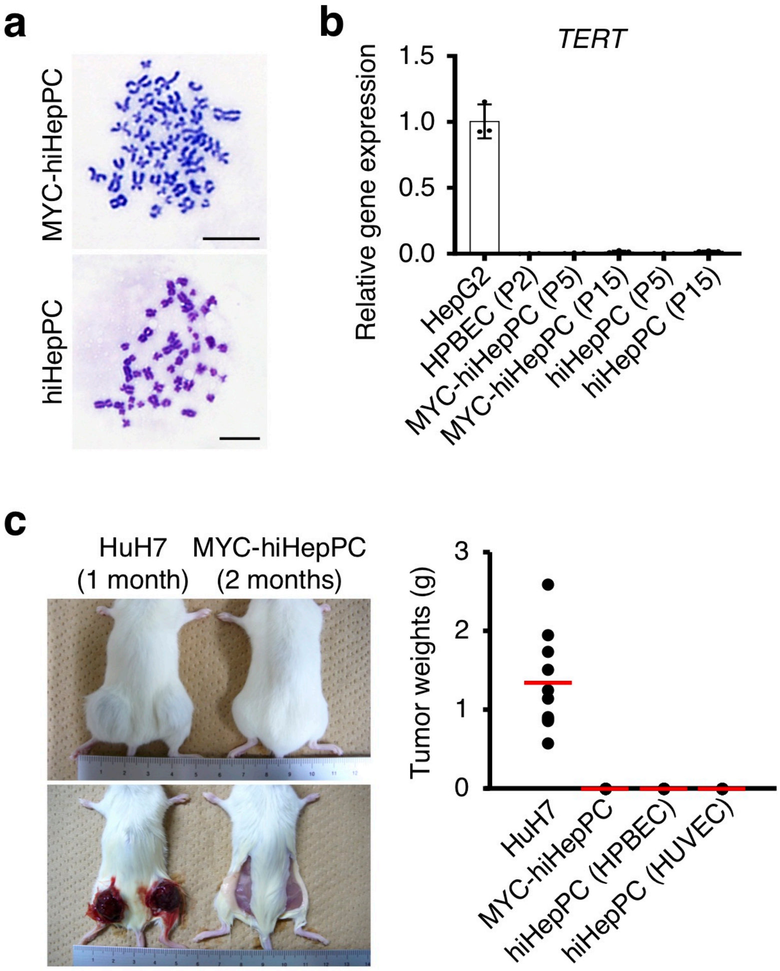

Supplementary Figure 18. MYC-hiHepPCs have normal karyotypes and are not tumorigenic *in vivo*. (**a**) Representative images of karyotypes of a HPBEC-derived MYC-hiHepPC and hiHepPC at passage 14 in monolayer culture. Note that the numbers of chromosomes in all 20 MYC-hiHepPCs and 20 hiHepPCs analyzed in this study were normal. Scale bar, 10 μm. (**b**) qPCR analyses of *TERT* were performed on total RNA obtained from HepG2, HPBECs at passage (P) 2, and HPBEC-derived MYC-hiHepPCs and hiHepPCs at P5 and P15 in monolayer culture. All data were normalized with the values for HepG2, and the fold differences are shown. Data represent the mean ± SD (n = 3 independent assays). (**c**) Representative images of immunodeficient NOD/SCID mice 1 month and 2 months after subcutaneous injection of the human hepatocellular carcinoma cell line HuH7 and HPBEC-derived MYC-hiHepPCs, respectively (n = 5 mice were used for the injection of each cell type). Note that tumors were formed from only HuH7. The graph shows the weights of tumors formed from HuH7 1 month after injection and HPBEC-derived MYC-hiHepPCs and hiHepPCs and HUVEC-derived hiHepPCs 2 months after injection. Means of the data are shown with red lines (n = 10 tumors). Any tumors were not formed from both MYC-hiHepPCs and hiHepPCs. Source data are provided as a Source Data file.

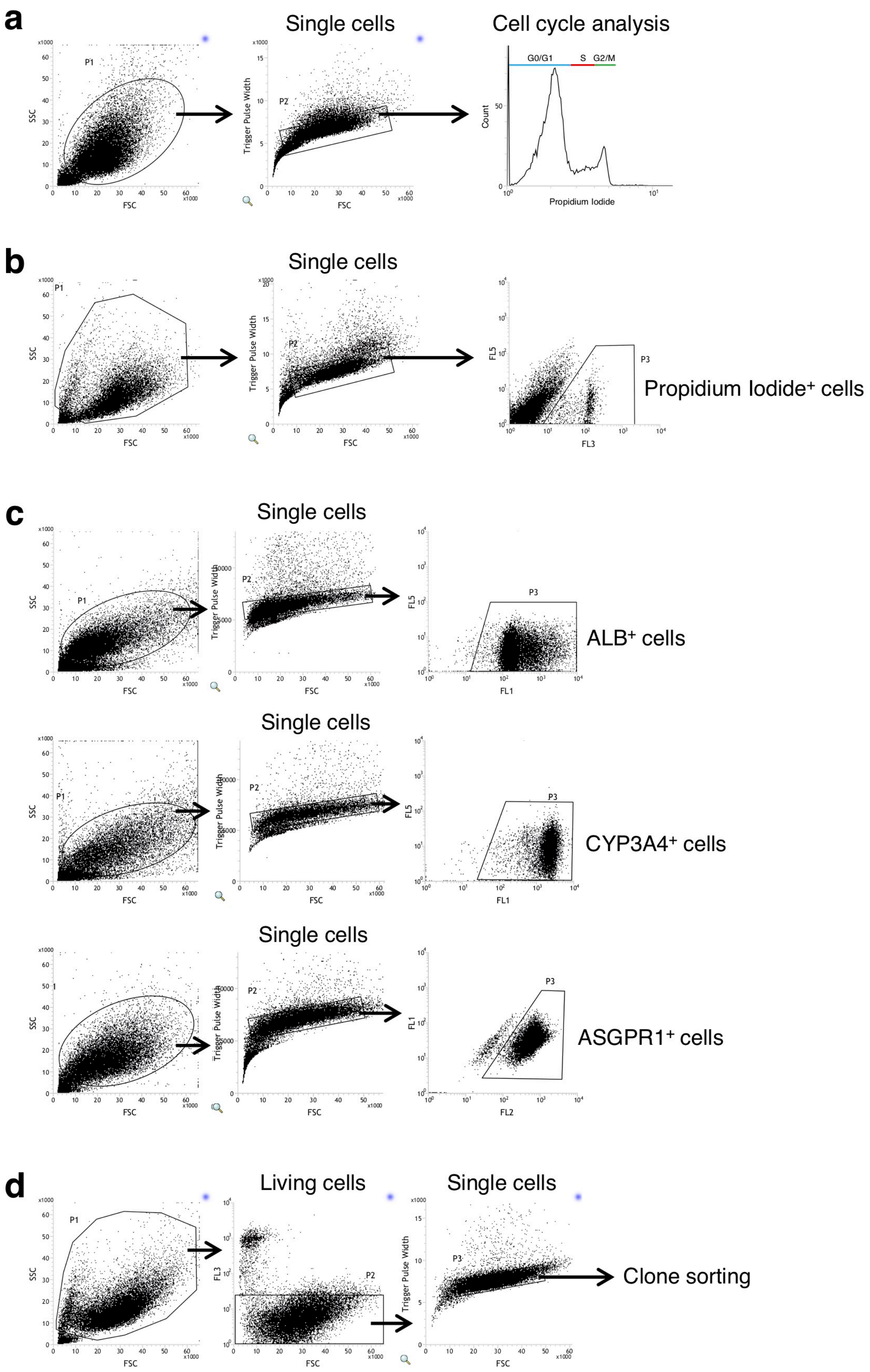

Supplementary Figure 19. Gating strategies used for cell analysis and sorting. (**a–c**) Gating strategies to analyze the cell cycle of hiHepPCs in monolayer cultures (**a**), the percentages of propidium iodide^{+} dead cells contained in hiHepPC monolayer cultures and hiHepPC aggregates (**b**), and the percentages of cells immunoreactive for ALB, CYP3A4, or ASGPR1 among cells composing hiHepPC aggregates (**c**). (**d**) Gating strategy to conduct clone sorting of hiHepPCs for single-cell culture analyses.

Supplementary Table 1. The list of qPCR primers.

	Gene	Forward 5'-3'	Reverse 5'-3'
**qPCR primers**	*AFP*	CCTGAAGGTCTATCTCCAAATCTAAACA	GAATTACTGAGACAGCAAGCTGAGGA
	DLK1	TGCAACCCCCAAAATGGA	GGTTCTCCACAGAGTCCGTGA
	SOX17	AGCAGAATCCAGACCTGCAC	TCTGCCTCCTCCACGAAG
	CXCR4	TGAGAAGCATGACGGACAAG	GACGCCAACATAGACCACCT
	TERT	[6]	[6]
	CK19	AGCCGGACTGAAGAATTGAA	TCTTCCAAGGCAGCTTTCAT
	HNF1B	CACCATAATCCCCAGCAATC	GCCACACTGTTGATGACAGG
	SOX9	GCCAATAAGTGCCCGAGCA	AGCAAGTGGAATGTCTTGAAGGTTA
	CFTR	AGCATTTGCTGATTGCACAG	ACTGCCGCACTTTGTTCTCT
	SCTR	GGCACTCCTACCTGCTGAAG	GTAGTTGCGAGTGCAGTGGA
	EpCAM	GCTGGTGTGTGAACACTGCT	ACGCGTTGTGATCTCCTTCT
	ALB	TCATTAGCTGCTGATTTTGTTGAAAGTA	GGATGCCTTCTTGCATATTCATACA
	AAT	GGGAAACTACAGCACCTGGA	CCCCATTGCTGAAGACCTTA
	HNF4A	GCTCGGAGCCACCAAGAGA	CTCGTCAAGGATGCGTATGGA
	CDX1	TCGGACCAAGGACAAGTACC	TGTTGCTGCTGCTGTTTCTT
	CDX2	AGTCCCTCGGCAGCCAAGTG	TGGTGATGTAGCGACTGTAGTG
	OLFM4	[5]	[5]
	SI	GGTAAGGAGAAACCGGGAAG	GCACGTCGACCTATGGAAAT
	TFF3	[5]	[5]
	FOXA3 (exo)	AGGTGGGGAGCCTGGAGTCTACTA	AATCTTTTATTTTATCGATGCGGC
	HNF1A (exo)	ACTCCAGCAATGGCCAGA	AATCTTTTATTTTATCGTCG
	HNF6 (exo)	GAGTCTGGACAAGTGGCAGGA	AATCTTTTATTTTATCGATGCGGC
	GAPDH	GCACCGTCAAGGCTGAGAAC	TGGTGAAGACGCCAGTGGA

Supplementary Table 2. The list of antibodies.

	Antibody	Company	Catalog number	Dilution
**Primary antibodies**	Goat anti-Human ALB	Bethyl	A80-229A	1:500
	Goat anti-Human ALB (HRP-conjugated)	Bethyl	A80-229P	1:1000
	Goat anti-Human ALB (FITC-conjugated)	Bethyl	A80-229F	1:1000
	Rabbit anti-Mouse ALB	Bethyl	A90-135	1:500
	Goat anti-Mouse ALB	Bethyl	A90-134	1:500
	Rabbit anti-Human AAT	Neomarkers	Rb-367-A1	1:1000
	Mouse anti-ASGPR1	Santa Cruz Biotechnology	sc52623	1:100
	Mouse anti-ASGPR1 (PE-conjugated)	BD Biosciences	563655	1:100
	Rabbit anti-CYP3A4	Abcam	ab3572	1:1000
	Rabbit anti-Transferrin	Santa Cruz Biotechnology	sc-21011	1:500
	Goat anti-CD31	Santa Cruz Biotechnology	sc1506	1:1000
	Mouse anti-AFP	Santa Cruz Biotechnology	sc8399	1:100
	Mouse anti-AFP	R&D Systems	MAB1368	1:100
	Rabbit anti-AFP	MP Biomedicals	688031	1:2000
	Rabbit anti-DLK1	Abcam	ab21682	1:100
	Mouse anti-HNF4A	R&D Systems	PP-K9218-00	1:200
	Mouse anti-HNF4A	PPMX	PP-H1415-00	1:500
	Rat anti-E-CAD (ECCD2)	a gift from M. Takeichi		1:1000
	Rabbit anti-E-CAD	Cell Signaling Technology	3195S	1:250
	Mouse anti-EZRIN	Abcam	ab4069	1:500
	Mouse anti-CFTR	Millipore	MAB3484	1:500
	Mouse anti-Human CK19	Cell Signaling Technology	4558	1:500
	Rabbit anti-Mouse CK19	[7]		1:1000
	Rabbit anti-SOX9	Millipore	AB5535	1:500
	Rabbit anti-HNF1B	Santa Cruz Biotechnology	sc22840	1:500
	Mouse anti-α-TUBULIN	Sigma-Aldrich	T6743	1:500
	Rabbit anti-EpCAM	Abcam	ab71916	1:1000
	Rabbit anti-ZO-1	Zymed	40-2200	1:500
	Mouse anti-Human CK8/18	Leica	NCL-5D3	1:500
	Mouse anti-MRP2	Abcam	ab3373	1:200
	Rabbit anti-MRP2	Sigma-Aldrich	M-8316	1:500
	Mouse anti-Human CDX2	MBL	MU392AUC	1:500
	Rabbit anti-Cleaved caspase-3	Cell Signaling Technology	D175	1:100
	Rabbit anti-Ki67	Abcam	ab833	1:500
	Mouse anti-BrdU	BD Biosciences	347580	1:100
**Secondary antibodies**	Alexa 488-conjugated donkey anti-rabbit IgG	Molecular Probes	A21206	1:2000
	Alexa 488-conjugated donkey anti-goat IgG	Molecular Probes	A11055	1:2000
	Alexa 488-conjugated donkey anti-mouse IgG	Molecular Probes	A21202	1:2000
	Alexa 488-conjugated donkey anti-rat IgG	Molecular Probes	A21208	1:2000
	Alexa 555-conjugated donkey anti-rabbit IgG	Molecular Probes	A31572	1:2000
	Alexa 555-conjugated donkey anti-goat IgG	Molecular Probes	A21432	1:2000
	Alexa 555-conjugated donkey anti-mouse IgG	Molecular Probes	A31570	1:2000
	Alexa 594-conjugated donkey anti-rat IgG	Molecular Probes	A21209	1:2000
	Alexa 647-conjugated donkey anti-goat IgG	Molecular Probes	A21447	1:1000
	Alexa 647-conjugated donkey anti-rabbit IgG	Molecular Probes	A31573	1:1000

Supplementary References

1. Ang, L. T. et al. A roadmap for human liver differentiation from pluripotent stem cells. *Cell Rep.* **22**, 2190–2205 (2018).
2. Huch, M. et al. Long-term culture of genome-stable bipotent stem cells from adult human liver. *Cell* **160**, 299–312 (2015).
3. Du, Y. et al. Human hepatocytes with drug metabolic function induced from fibroblasts by lineage reprogramming. *Cell Stem Cell* **14**, 394–403 (2014).
4. Huang, P. et al. Direct reprogramming of human fibroblasts to functional and expandable hepatocytes. *Cell Stem Cell* **14**, 370–384 (2014).
5. Miura, S. & Suzuki, A. Generation of mouse and human organoid-forming intestinal progenitor cells by direct lineage reprogramming. *Cell Stem Cell* **21**, 456–471 (2017).
6. Fujiki, T. et al. TAK1 represses transcription of the human telomerase reverse transcriptase gene. *Oncogene* **26**, 5258–5266 (2007).
7. Sekiya, S. & Suzuki, A. Intrahepatic cholangiocarcinoma can arise from Notch-mediated conversion of hepatocytes. *J. Clin. Invest.* **122**, 3914–3918 (2012).
