# Supplementary material for: Direct reprogramming of human umbilical vein- and peripheral blood-derived endothelial cells into hepatic progenitor cells
Source: Nat Commun. 2020 Oct 21;11:5292. doi: 10.1038/s41467-020-19041-z (PMC7578104; doi:10.1038/s41467-020-19041-z)
Supplement: Supplementary file 2 — Reporting Summary [file 41467_2020_19041_MOESM2_ESM.pdf]

nature research

Corresponding author(s): Atsushi Suzuki

Last updated by author(s): Aug 11, 2020

Reporting Summary

Nature Research wishes to improve the reproducibility of the work that we publish. This form provides structure for consistency and transparency in reporting. For further information on Nature Research policies, see our Editorial Policies and the Editorial Policy Checklist.

Statistics

For all statistical analyses, confirm that the following items are present in the figure legend, table legend, main text, or Methods section.

n/a	Confirmed	
☐	☒	The exact sample size (*n*) for each experimental group/condition, given as a discrete number and unit of measurement
☐	☒	A statement on whether measurements were taken from distinct samples or whether the same sample was measured repeatedly
☐	☒	The statistical test(s) used AND whether they are one- or two-sided<br>*Only common tests should be described solely by name; describe more complex techniques in the Methods section.*
☐	☒	A description of all covariates tested
☐	☒	A description of any assumptions or corrections, such as tests of normality and adjustment for multiple comparisons
☐	☒	A full description of the statistical parameters including central tendency (e.g. means) or other basic estimates (e.g. regression coefficient) AND variation (e.g. standard deviation) or associated estimates of uncertainty (e.g. confidence intervals)
☐	☒	For null hypothesis testing, the test statistic (e.g. *F*, *t*, *r*) with confidence intervals, effect sizes, degrees of freedom and *P* value noted<br>*Give P values as exact values whenever suitable.*
☒	☐	For Bayesian analysis, information on the choice of priors and Markov chain Monte Carlo settings
☒	☐	For hierarchical and complex designs, identification of the appropriate level for tests and full reporting of outcomes
☒	☐	Estimates of effect sizes (e.g. Cohen's *d*, Pearson's *r*), indicating how they were calculated

Our web collection on statistics for biologists contains articles on many of the points above.

Software and code

Policy information about availability of computer code

Data collection	FACS Jazz (BD Biosciences); Multiskan FC microplate reader (Thermo Fisher Scientific); Luminescencer Octa (ATTO); IX71 and IX73 fluorescence microscopes (Olympus); FLUOVIEW FV10i confocal microscope (Olympus); 7300 Real Time PCR System (Applied Biosystems); HiSeq 1500 system (Illumina)
Data analysis	BD FACS Sortware sorter software; TCC (v1.12.0), DESeq2 (v1.26.0), edgeR (v3.28.1), and rgl (v0.99.16) packages on R software (v3.6.0); GEO2R program in the Gene Expression Omnibus (GEO) website; GSEAPreranked program (v5) in GSEA software (v3.0); Agilent Genomic Workbench software (v7.0); Microsoft Excel software (v16.16); Database for Annotation, Visualization, and Integrated Discovery (DAVID) (v6.8); Bowtie2 (v2.3.0)

For manuscripts utilizing custom algorithms or software that are central to the research but not yet described in published literature, software must be made available to editors and reviewers. We strongly encourage code deposition in a community repository (e.g. GitHub). See the Nature Research guidelines for submitting code & software for further information.

Data

Policy information about availability of data

All manuscripts must include a data availability statement. This statement should provide the following information, where applicable:

- Accession codes, unique identifiers, or web links for publicly available datasets
- A list of figures that have associated raw data
- A description of any restrictions on data availability

All datasets were deposited in the GEO database under accession numbers GEO: GSE118910 [https://www.ncbi.nlm.nih.gov/geo/query/acc.cgi?acc=GSE118910] and GEO: GSE120732 [https://www.ncbi.nlm.nih.gov/geo/query/acc.cgi?acc=GSE120732]. The publicly available datasets used in this study are GEO: GSE42643 [https://www.ncbi.nlm.nih.gov/geo/query/acc.cgi?acc=GSE42643], GSE63859 [https://www.ncbi.nlm.nih.gov/geo/query/acc.cgi?acc=GSE63859], GSE98324

[https://www.ncbi.nlm.nih.gov/geo/query/acc.cgi?acc=GSE98324], and GEO: GSE54066 [https://www.ncbi.nlm.nih.gov/geo/query/acc.cgi?acc=GSE54066].

Field-specific reporting

Please select the one below that is the best fit for your research. If you are not sure, read the appropriate sections before making your selection.

☒ Life sciences ☐ Behavioural & social sciences ☐ Ecological, evolutionary & environmental sciences

For a reference copy of the document with all sections, see nature.com/documents/nr-reporting-summary-flat.pdf

Life sciences study design

All studies must disclose on these points even when the disclosure is negative.

Sample size	For in vitro and in vivo experiments, sample size for each experiment has been calculated based on previous data collection published in Sekiya and Suzuki Nature 2011 and Miura and Suzuki Cell Stem Cell 2017.
Data exclusions	No data were excluded.
Replication	All experiments were conducted at least three times independently, and all attempts at replication were successful.
Randomization	All samples (cells, mice, etc.) used in the experiments were randomly allocated into different experimental groups.
Blinding	Experimenters were blinded to group allocation for transcriptome analysis and CGH analysis. Blinding was also used for the donors of peripheral blood. All other experiments were conducted in a non-blinded manner, because the research design was complicated, the researchers were restricted, and blinding feasibility was poor.

Reporting for specific materials, systems and methods

We require information from authors about some types of materials, experimental systems and methods used in many studies. Here, indicate whether each material, system or method listed is relevant to your study. If you are not sure if a list item applies to your research, read the appropriate section before selecting a response.

Materials & experimental systems

n/a	Involved in the study	
☐	☒	Antibodies
☐	☒	Eukaryotic cell lines
☒	☐	Palaeontology and archaeology
☐	☒	Animals and other organisms
☐	☒	Human research participants
☒	☐	Clinical data
☒	☐	Dual use research of concern

Methods

n/a	Involved in the study	
☒	☐	ChIP-seq
☐	☒	Flow cytometry
☒	☐	MRI-based neuroimaging

Antibodies

Antibodies used	All the primary and secondary antibodies used in this study were described in Supplementary Table 2. Primary antibodies: Goat anti-Human ALB (Bethyl, A80-229A), Goat anti-Human ALB (HRP-conjugated) (Bethyl, A80-229P), Goat anti-Human ALB (FITC-conjugated) (Bethyl, A80-229F), Rabbit anti-Mouse ALB (Bethyl, A90-135), Goat anti-Mouse ALB (Bethyl, A90-134), Rabbit anti-Human AAT (Neomarkers, Rb-367-A1), Mouse anti-ASGPR1 (Santa Cruz Biotechnology, sc52623), Mouse anti-ASGPR1 (PE-conjugated) (BD Biosciences, 563655), Rabbit anti-CYP3A4 (Abcam, ab3572), Rabbit anti-Transferrin (Santa Cruz Biotechnology, sc-21011), Goat anti-CD31 (Santa Cruz Biotechnology, sc1506), Mouse anti-AFP (Santa Cruz Biotechnology, sc8399), Mouse anti-AFP (R&D Systems, MAB1368), Rabbit anti-AFP (MP Biomedicals, 688031), Rabbit anti-DLK1 (Abcam, ab21682), Mouse anti-HNF4A (R&D Systems, PP-K9218-00), Mouse anti-HNF4A (PPMX, PP-H1415-00), Rat anti-E-CAD (ECCD2) (made in Takeichi lab, RIKEN, Japan), Rabbit anti-E-CAD (Cell Signaling Technology, 3195S), Mouse anti-EZRIN (Abcam, ab4069), Mouse anti-CFTR (Millipore, MAB3484), Mouse anti-Human CK19 (Cell Signaling Technology, 4558), Rabbit anti-Mouse CK19 (made in Suzuki lab, Kyushu University, Japan), Rabbit anti-SOX9 (Millipore, AB5535), Rabbit anti-HNF1B (Santa Cruz Biotechnology, sc22840), Mouse anti-α-TUBULIN (Sigma-Aldrich, T6743), Rabbit anti-EpCAM (Abcam, ab71916), Rabbit anti-ZO-1 (Zymed, 40-2200), Mouse anti-Human CK8/18 (Leica, NCL-5D3), Mouse anti-MRP2 (Abcam, ab3373), Rabbit anti-MRP2 (Sigma-Aldrich, M-8316), Mouse anti-Human CDX2 (MBL, MU392AUC), Rabbit anti-Cleaved caspase-3 (Cell Signaling Technology, D175), Rabbit anti-Ki67 (Abcam, ab833), and Mouse anti-BrdU (BD Biosciences, 347580). Secondary antibodies: Alexa 488-conjugated donkey anti-rabbit IgG (Molecular Probes, A21206), Alexa 488-conjugated donkey anti-goat IgG (Molecular Probes, A11055), Alexa 488-conjugated donkey anti-mouse IgG (Molecular Probes, A21202), Alexa 488-conjugated donkey anti-rat IgG (Molecular Probes, A21208), Alexa 555-conjugated donkey anti-rabbit IgG (Molecular Probes, A31572), Alexa 555-conjugated donkey anti-goat IgG (Molecular Probes, A21432), Alexa 555-conjugated donkey anti-mouse IgG (Molecular Probes, A31570), Alexa 594-conjugated donkey anti-rat IgG (Molecular Probes, A21209), Alexa 647-conjugated donkey anti-goat IgG (Molecular Probes, A21447), and Alexa 647-conjugated donkey anti-rabbit IgG (Molecular Probes, A31573).

Validation	All commercial antibodies were validated by the vendors and documented by corresponding data sheets, and the others were validated in the study reported previously. Primary antibodies: Goat anti-Human ALB (Bethyl, A80-229A), human, IF and IHC and FACS, (https://www.bethyl.com/product/A80-229A); Goat anti-Human ALB (HRP-conjugated) (Bethyl, A80-229P), human, IF, (https://www.bethyl.com/product/A80-229P); Goat anti-Human ALB (FITC-conjugated) (Bethyl, A80-229F), human, IF, (https://www.bethyl.com/product/A80-229F?target=&referrer=search); Goat anti-Mouse ALB (Bethyl, A90-134), mouse, IF, (https://www.bethyl.com/product/A90-135); Goat anti-Mouse ALB (Bethyl, A90-134), mouse, IHC, (https://www.bethyl.com/product/A90-134A/); Rabbit anti-Human AAT (Neomarkers, Rb-367-A1), human, IF and IHC, (https://assets.thermofisher.com/TFS-Assets/APD/Specification-Sheets/D11808~.pdf); Mouse anti-ASGPR1 (Santa Cruz Biotechnology, sc52623), human and mouse, IF, (https://datasheets.scbt.com/sc-52623.pdf); Mouse anti-ASGPR1 (PE-conjugated) (BD Biosciences, 563655), human and rat, FACS, (https://www.bdbiosciences.com/eu/applications/research/stem-cell-research/endoderm-markers/human/pe-mouse-anti-asgpr-1-8d7/p/563655); Rabbit anti-CYP3A4 (Abcam, ab3572), human and mouse, IF and FACS (https://www.abcam.co.jp/cytochrome-p450-3a4cyp3a4-antibody-ab3572.html); Rabbit anti-Transferrin (Santa Cruz Biotechnology, sc-21011), human, IF (https://www.antibodypedia.com/gene/873/TF/antibody/12340/sc-21011); Goat anti-CD31 (Santa Cruz Biotechnology, sc1506), human and mouse, IF (https://www.scbt.com/p/pecam-1-antibody-m-20/); Mouse anti-AFP (R&D Systems, MAB1368), human and mouse, IF and IHC (https://www.scbt.com/p/afp-antibody-c3/); Rabbit anti-AFP (MP Biomedicals, 688031), human and mouse, IF and IHC, (https://www.rndsystems.com/products/human-mouse-alpha-fetoprotein-afp-antibody-189502_mab1368); Mouse anti-AFP (Santa Cruz Biotechnology, sc8399), human , IF, (https://www.mpbio.com/us/08688031-rabbit-anti-alpha-fetoprotein-afp-polyclonal); Rabbit anti-AFP (MP Biomedicals, 688031), human and mouse, IF, (https://www.abcam.co.jp/dlk-1-antibody-ab21682.html); Mouse anti-HNF4A (R&D Systems, PP-K9218-00), human, IF, (https://www.rndsystems.com/products/human-hnf-4-alpha-nr2a1-antibody-k9218_pp-k9218-00); Mouse anti-HNF4A (PPMX, PP-H1415-00), human, IF and IHC, (https://www.ppmx.com/img/SDS_E_H1415-00_HNF4a.pdf); Rat anti-E-CAD (ECCD2) (made in Takeichi lab, RIKEN, Japan), human, IF, (https://www.jstage.jst.go.jp/article/csf1975/11/3/11_3_245/_article); Rabbit anti-E-CAD (Cell Signaling Technology, 3195S), human and mouse, IF, (https://www.biocompare.com/9776-Antibodies/1543011-ECadherin-24E10-Rabbit-mAb/); Mouse anti-EZRIN (Abcam, ab4069), human and mouse, IF, (https://www.abcam.co.jp/ezrin-antibody-3c12-ab4069.html); Mouse anti-CFTR (Millipore, MAB3484), human and mouse, IF (https://www.merckmillipore.com/JP/ja/product/Anti-Cystic-Fibrosis-Transmembrane-Conductance-Regulator-Antibody-a.a.-386-412-clone-L12B4,MM_NF-MAB3484); Mouse anti-Human CK19 (Cell Signaling Technology, 4558), human, IF and IHC, (https://www.citeab.com/antibodies/124510-4558-keratin-19-ba17-mouse-mab); Rabbit anti-Mouse CK19 (made in Suzuki lab, Kyushu University, Japan), mouse, IF and IHC, (https://www.jci.org/articles/view/63065); Rabbit anti-SOX9 (Millipore, AB5535), human and mouse, IF and IHC, (https://www.merckmillipore.com/JP/ja/product/Anti-Sox9-Antibody,MM_NF-AB5535); Rabbit anti-HNF1B (Santa Cruz Biotechnology, sc22840), human , IF, (,https://www.scbt.com/p/hnf-1beta-antibody-h-85?requestFrom=search); Rabbit anti-EpCAM (Abcam, ab71916), human and mouse , IF, (https://www.abcam.co.jp/EpCAM-antibody-ab71916.html); Rabbit anti-ZO-1 (Zymed, 40-2200), human and mouse, IF, (https://www.thermofisher.com/antibody/product/ZO-1-Antibody-Polyclonal/40-2200); Mouse anti-Human CK8/18 (Leica, NCL-5D3), human, IHC, (https://shop.leicabiosystems.com/us/ihc-ish/ihc-primary-antibodies/pid-cytokeratin-818); Mouse anti-MRP2 (Abcam, ab3373), human, IF, (https://www.abcam.co.jp/MRP2-antibody-M2-III-6-ab3373.html); Rabbit anti-MRP2 (Sigma-Aldrich, M-8316), human, IHC, (https://www.sigmaaldrich.com/catalog/product/sigma/m8316?lang=ja&region=JP); Mouse anti-Human CDX2 (MBL, MU392AUC), human and mouse, IF (https://www.labome.com/product/Biogenex/MU392A-UC.html); Rabbit anti-Cleaved caspase-3 (Cell Signaling Technology, D175), human and mouse, IF, (https://www.biocompare.com/Product-Reviews/40504-Cell-Signaling-Technology-s-Cleaved-Caspase-3-D175-Antibody/); Rabbit anti-Ki67 (Abcam, ab833), human, IF, (https://www.abcam.co.jp/ki67-antibody-ab833.html); Mouse anti-BrdU (BD Biosciences, 347580), mouse, IHC, (https://www.bdbiosciences.com/us/applications/research/apoptosis/purified-antibodies/purified-mouse-anti-brdu-b44/p/347580). Secondary antibodies: Alexa 488-conjugated donkey anti-rabbit IgG (Molecular Probes, A21206), IF and FACS, (https://www.thermofisher.com/antibody/product/Donkey-anti-Rabbit-IgG-H-L-Highly-Cross-Adsorbed-Secondary-Antibody-Polyclonal/A-21206); Alexa 488-conjugated donkey anti-goat IgG (Molecular Probes, A11055), IF and FACS, (https://www.thermofisher.com/antibody/product/Donkey-anti-Goat-IgG-H-L-Cross-Adsorbed-Secondary-Antibody-Polyclonal/A-11055); Alexa 488-conjugated donkey anti-mouse IgG (Molecular Probes, A21202), IF and FACS, (https://www.thermofisher.com/antibody/product/Donkey-anti-Mouse-IgG-H-L-Highly-Cross-Adsorbed-Secondary-Antibody-Polyclonal/A-21202); Alexa 555-conjugated donkey anti-rabbit IgG (Molecular Probes, A31572), IF, (https://www.thermofisher.com/antibody/product/Donkey-anti-Rabbit-IgG-H-L-Highly-Cross-Adsorbed-Secondary-Antibody-Polyclonal/A-31572); Alexa 555-conjugated donkey anti-goat IgG (Molecular Probes, A21432), IF, (https://www.thermofisher.com/antibody/product/Donkey-anti-Goat-IgG-H-L-Cross-Adsorbed-Secondary-Antibody-Polyclonal/A-21432); Alexa 555-conjugated donkey anti-mouse IgG (Molecular Probes, A31570), IF, (https://www.thermofisher.com/antibody/product/Donkey-anti-Mouse-IgG-H-L-Highly-Cross-Adsorbed-Secondary-Antibody-Polyclonal/A-31570); Alexa 594-conjugated donkey anti-rat IgG (Molecular Probes, A21209), IF, (https://www.thermofisher.com/antibody/product/Donkey-anti-Rat-IgG-H-L-Highly-Cross-Adsorbed-Secondary-Antibody-Polyclonal/A-21209); Alexa 647-conjugated donkey anti-goat IgG (Molecular Probes, A21447), IF, (https://www.thermofisher.com/antibody/product/Donkey-anti-Goat-IgG-H-L-Cross-Adsorbed-Secondary-Antibody-Polyclonal/A-21447); Alexa 647-conjugated donkey anti-rabbit IgG (Molecular Probes, A31573), IF (https://www.thermofisher.com/antibody/product/Donkey-anti-Rabbit-IgG-H-L-Highly-Cross-Adsorbed-Secondary-Antibody-Polyclonal/A-31573)

Eukaryotic cell lines

Policy information about cell lines

Cell line source(s)	Plat-GP cells (Cell Biolabs), HepG2 (RIKEN BRC, RCB1886), and HuH7 (RIKEN BRC, RCB1942).
Authentication	Cell line authentication was performed based on their morphology, growth condition, and specific properties.
Mycoplasma contamination	All the cell lines were tested negative for mycoplasma contamination.
Commonly misidentified lines (See ICLAC register)	No commonly misidentified cell lines were used in the study.

Animals and other organisms

Policy information about studies involving animals; ARRIVE guidelines recommended for reporting animal research

Laboratory animals	NSG (NOD.Cg-Prkdcscidll2rgtm1Wjl/SzJ) male mice (3 and 10 weeks old), NOD/SCID female mice (10 weeks old) (both from Charles River Laboratories), C57BL/6 male mice (8 weeks old) (Clea), C57BL/6 neonatal mice (2 days old), and C57BL/6 mouse embryos (embryonic days 12.5 and 16.5) were used in this study. Mice were housed in groups of 2-4 per cage in a 12 h light/dark cycle (08:00-20:00 light; 20:00-8:00 dark), with controlled room temperature (22 ± 4°C) and relative humidity (60%).
Wild animals	No wild animals were used in the study.
Field-collected samples	No field-collected samples were used in this study.
Ethics oversight	The experiments were approved by the Kyushu University Animal Experiment Committee, and the care of the animals was in accordance with institutional guidelines.

Note that full information on the approval of the study protocol must also be provided in the manuscript.

Human research participants

Policy information about studies involving human research participants

Population characteristics	Adult human peripheral blood was obtained from healthy donors over twenty years of age. Any donor information was not obtained in this study.
Recruitment	All the research subjects were volunteers. Volunteers were recruited from the members in the laboratory of Suzuki (corresponding author) and those of collaborators after a detailed explanation of the experimental design. There was no bias in the selection of research subjects.
Ethics oversight	The collection of adult human peripheral blood was approved by the Kyushu University Ethics Committee and performed after obtaining informed consent.

Note that full information on the approval of the study protocol must also be provided in the manuscript.

Flow Cytometry

Plots

Confirm that:

- [x] The axis labels state the marker and fluorochrome used (e.g. CD4-FITC).
- [x] The axis scales are clearly visible. Include numbers along axes only for bottom left plot of group (a 'group' is an analysis of identical markers).
- [x] All plots are contour plots with outliers or pseudocolor plots.
- [x] A numerical value for number of cells or percentage (with statistics) is provided.

Methodology

Sample preparation	For the clone-sorting of living cells and the analysis of cell-death, single cells collected from the monolayer cultures and cell-aggregation cultures were incubated with propidium iodide (PI) and washed with phosphate-buffered saline (PBS). For the analyses of protein expression and cell cycle, single cells collected from the monolayer cultures and cell-aggregation cultures were fixed and stained with antibodies or incubated with PI, and washed with PBS.
Instrument	FACS Jazz (BD Biosciences)
Software	BD FACS Sortware sorter software
Cell population abundance	For single-cell culture analyses, PI-negative living cells were isolated and cultured in individual wells of type I collagen-coated 96-well plates (1 cell/well). The purity of PI-negative cells in the isolated cell population was confirmed by flow cytometric analysis.
Gating strategy	Gating strategy is shown in Supplementary Fig. 19. Cells were initially gated on FSC/SSC to exclude debris, and then only single cells were gated. Antigen-positive and negative cells were identified based on the data of negative controls (eg. samples stained with only 2nd antibody).

- [x] Tick this box to confirm that a figure exemplifying the gating strategy is provided in the Supplementary Information.
